# PreTSA: computationally efficient modeling of temporal and spatial gene expression patterns

**DOI:** 10.1186/s13059-026-03994-3

**Published:** 2026-02-12

**Authors:** Haotian Zhuang, Zhicheng Ji

**Affiliations:** Department of Biostatistics and Bioinformatics, Duke University School of Medicine, Durham, NC USA

## Abstract

Modeling temporal and spatial gene expression patterns in large-scale single-cell and spatial transcriptomics data is a computationally intensive task. We present PreTSA, a method that offers computational efficiency in modeling these patterns and is applicable to single-cell and spatial transcriptomics data comprising millions of cells. PreTSA consistently matches the results of state-of-the-art methods while significantly reducing computational time. PreTSA provides a unique solution for studying gene expression patterns in extremely large datasets.

**Supplementary Information:**

The online version contains supplementary material available at 10.1186/s13059-026-03994-3.

## Background

Single-cell RNA-sequencing (scRNA-seq) and spatial transcriptomics (ST) technologies offer unprecedented opportunities to study the temporal and spatial dynamics of gene expression with single-cell or near single-cell resolution. While scRNA-seq does not directly retain or measure the order of cells in continuous biological processes such as cell development and differentiation, such ordering information can be computationally inferred using pseudotime analysis [1–6]. This allows for the examination of how a gene’s expression level changes temporally along a continuous process [7–13]. ST, on the other hand, measures both the gene expression and spatial locations of single cells or small clusters of cells, enabling the direct interrogation of spatial gene expression patterns from the observed data [14–19].

A smooth function, such as that obtained by the generalized additive model (GAM), is often used to characterize the temporal and spatial patterns of gene expression. Smoothing helps to recover the real patterns of gene expression from noisy data and mitigates the visualization biases associated with plotting numerous data points [20]. Furthermore, a statistical model can be built upon the smooth function to identify temporally variable genes (TVGs) and spatially variable genes (SVGs) with statistical significance. GAM has been extensively used in pseudotime methods such as Monocle [1, 3], TSCAN [2], and Slingshot [4]. PseudotimeDE [21] further applies subsampling and permutation approaches to account for the uncertainty in pseudotime inference, thereby generating well-calibrated *p*-values for the identification of TVGs. While most existing methods for analyzing ST data, such as SpatialDE [22], SPARK [23], SPARK-X [24], and nnSVG [25], focus on identifying SVGs, GAM can also be extended to fit and visualize spatial gene expression patterns principally.

The number of cells included in scRNA-seq and ST datasets has been increasing exponentially in recent years, leading to datasets that often contain millions of cells [5, 26–30]. Most existing methods, however, were developed and tested on much smaller datasets, typically comprising only thousands of cells. Consequently, they may not be computationally feasible for handling such large datasets. For example, GAM involves complex computations such as penalized regression splines and must be fitted for each gene individually, which could be computationally intensive for large datasets with thousands of genes. The repeated subsampling and permutation employed by PseudotimeDE further exacerbates this issue. Additionally, nnSVG, a method for analyzing ST data, can take more than a minute to analyze a single gene and up to a week to analyze thousands of genes, despite its ability to scale linearly.

## Results

**Fig. 1:**
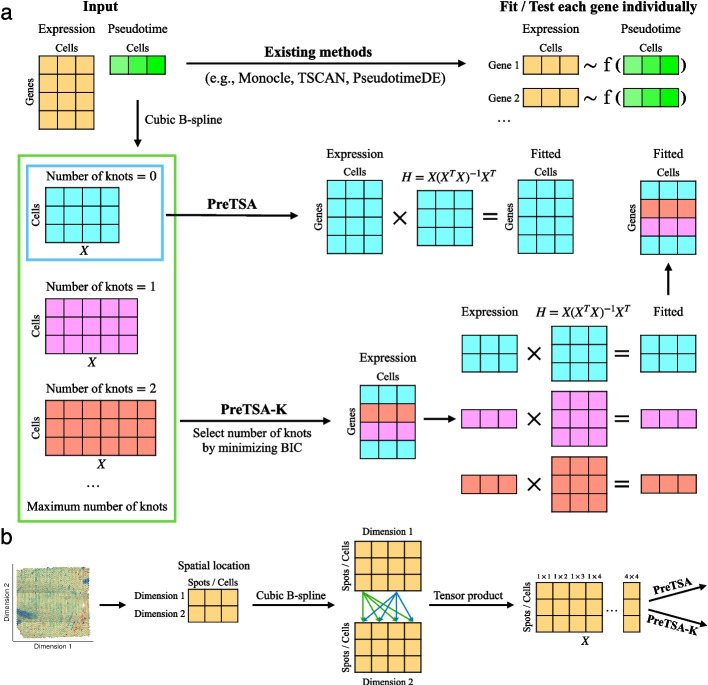
**a** A schematic view of PreTSA for modeling temporal gene expression patterns. **b** A schematic view of PreTSA for modeling spatial gene expression patterns

To address this issue, we developed PreTSA (Pattern recognition in Temporal and Spatial Analyses), a computationally efficient method designed to handle extremely large datasets. For each gene, PreTSA fits a regression model using B-splines, obtaining a smoothed curve that represents the relationship between gene expression values and pseudotime. Given the thousands of genes, this approach results in thousands of regression models. These models share the same design matrix, as the pseudotime values, which are specific to cells, remain consistent across different genes. Thus, PreTSA performs all computations related to the design matrix once, unlike existing methods such as GAM, which repeat these computations for each gene (Fig. 1a). This simplification substantially reduces the computational burden, particularly for large datasets. In addition, PreTSA leverages efficient matrix operations in R to further enhance computational efficiency. By default, PreTSA employs the simplest B-spline basis without internal knots to achieve optimal computational speed. Users have the option to select a flexible mode, PreTSA-K, which automatically determines the number of knots in the B-spline basis (Methods). Additional file 1: Figs. S1-2 show how different choices for the number of knots affect gene expression fitting. Additional file 1: Fig. S3 shows the number of cases in which PreTSA-K selects different values of K across all datasets in this study. PreTSA is recommended for maximum computational efficiency, while PreTSA-K should be used when balancing efficiency with fitting accuracy. PreTSA utilizes the PseudotimeDE framework for identifying TVGs through a subsampling and permutation procedure. To further improve computational efficiency, PreTSA also provides an option to identify TVGs using an F-test, which produces results similar to the permutation procedure (Additional file 1: Fig. S4). However, the *p*-values from the F-test are not as statistically rigorous as those obtained from permutation. In addition to serving as a standalone pipeline, PreTSA can also be integrated into other frameworks to replace GAM and increase computational efficiency.

**Fig. 2:**
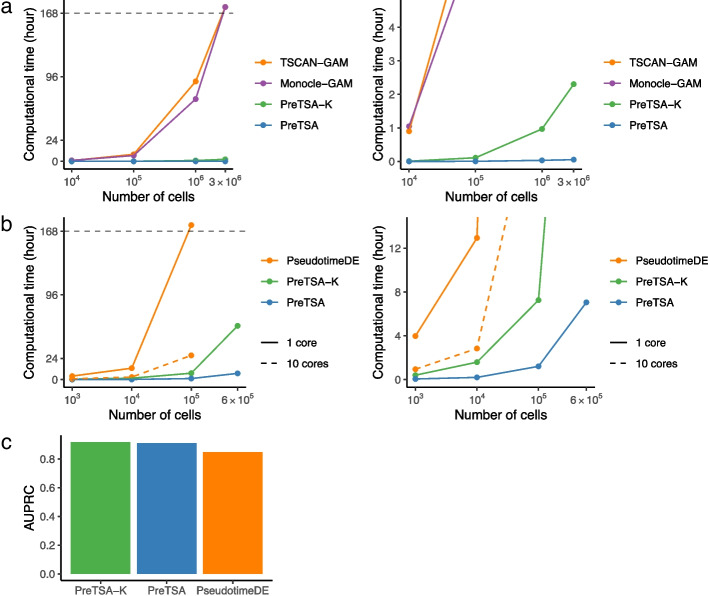
Simulation studies using the human PBMC scRNA-seq dataset. **a**,** b** Computational time (y-axes) of different methods for fitting the temporal pattern (**a**) and testing TVGs (**b**) in simulated data generated by sampling from the human PBMC scRNA-seq dataset with different numbers of cells (x-axes, log10 scale). Computational time more than one week (168 hours) is marked as timeout. For scenarios in which a method exceeded the 400 GB memory limit, its computational time could not be obtained and is therefore not shown in the plot. The right panels are zoom in views of the left panels. **c** Area under the precision-recall curve (AUPRC) for the simulation dataset generated by scDesign3, with methods ranked in decreasing order

We first compared the computational efficiency of PreTSA and GAM in modeling the temporal pattern of gene expression. We included two different implementations of GAM, as used in Monocle and TSCAN, in the comparison. We applied all methods to a simulated dataset derived from a real scRNA-seq dataset of peripheral blood mononuclear cells (PBMCs, Methods). The simulated dataset comprises 10,509 genes and ranges from thousands to millions of cells. PreTSA substantially outperformed GAM in computational efficiency (Fig. 2a). For analyzing one million cells, both versions of GAM required more than 70 hours, while PreTSA required only 2 minutes. For three million cells, GAM failed to complete the computation within a week (168 hours), whereas PreTSA took merely 3 minutes. PreTSA-K, which involves additional steps to select the number of knots, displayed computational efficiency comparable to PreTSA and was still much faster than GAM. PreTSA and PreTSA-K also required less memory than the GAM implementations (Additional file 1: Fig. S5). A similar disparity in computational efficiency was observed between PreTSA and PseudotimeDE in identifying TVGs, with both methods relying on permutation procedures involving 100 repetitions of fitting on subsampled data (Fig. 2b, Additional file 1: Fig. S6). For analyzing one hundred thousand cells, PseudotimeDE with a single core did not finish the computation within a week, while PreTSA took 1.2 hours. While PseudotimeDE with 10 cores substantially reduced computation time, it also required much larger memory and failed to process a dataset of 600,000 cells using 400 GB of memory. These results suggest that PreTSA is the only method capable of completing computations within a reasonable timeframe for large datasets. PreTSA also demonstrated improved accuracy in identifying TVGs, achieving a higher area under the precision-recall curve (AUPRC) on a simulation dataset generated by scDesign3 [31] with ground truth TVGs (Fig. 2c). Moreover, when applied to a null simulation dataset with permuted pseudotime values, PreTSA identified almost no TVGs, indicating its ability to effectively control false positives (Additional file 1: Fig. S7).

**Fig. 3:**
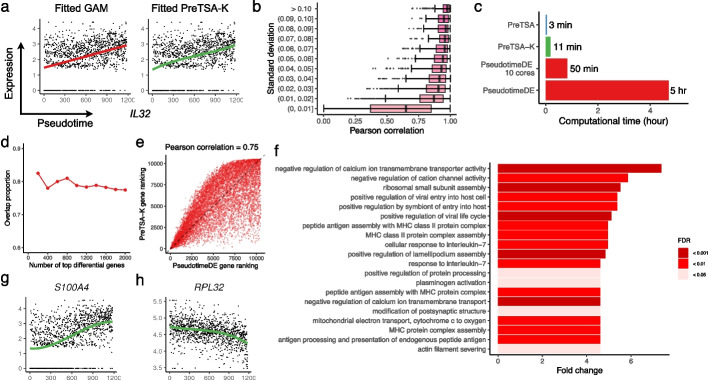
Analysis of the original human PBMC scRNA-seq dataset. **a** Scatterplots showing the expression of *IL32* (y-axis) and the pseudotime (x-axis). The curve indicates the fitted curve by TSCAN-GAM (left) or by PreTSA-K (right). **b** Pearson correlations between fitted values by TSCAN-GAM and by PreTSA-K (x-axis), grouped by the standard deviation of fitted values by TSCAN-GAM (y-axis). **c** Computational time of different methods for testing TVGs. **d** Overlap proportion for different numbers of top differential genes using PreTSA-K and PseudotimeDE. **e** Gene rankings by PreTSA-K (y-axis) and by PseudotimeDE (x-axis). **f** Top GO terms enriched among TVGs identified by PreTSA-K. **g**,** h** Expression (y-axis) of *S100A4* (**g**) and *RPL32* (**h**) along pseudotime (x-axis). Dots represent single-cell gene expression values, and curves indicate the fitted trajectories obtained by PreTSA-K

We next compared the temporal gene expression patterns modeled by PreTSA and GAM in the original PBMC dataset. Figure 3a displays the expression and fitted curves for the example gene, *IL32*, known to be associated with the T cell activation process [32]. The curves fitted by PreTSA-K and TSCAN-GAM are highly similar, both indicating linearly increasing trends. Figure 3b presents the Pearson correlation coefficients (PCCs) between the curves fitted by PreTSA-K and TSCAN-GAM for each gene, where the median PCC exceeds 0.9 for genes with standard deviations of GAM-fitted curves larger than 0.01. PreTSA and Monocle-GAM demonstrate highly similar findings (Additional file 1: Fig. S8a-d). Further, we compared the results of PreTSA and PseudotimeDE in identifying TVGs. Notably, in this smaller dataset comprising thousands of cells, PseudotimeDE with a single core required five hours to complete, whereas PreTSA took only three minutes and required less memory (Fig. 3c, Additional file 1: Fig. S9). Genes were ordered increasingly by the *p*-values from PreTSA-K and PseudotimeDE for identifying TVGs, respectively. The overlap in the top-ranked genes identified by both methods is approximately 80% (Fig. 3 d). The Pearson correlation coefficient for the overall gene rankings by both methods is as high as 0.75 (Fig. 3e). PreTSA yielded highly similar results (Additional file 1: Fig. S8e-f). These findings indicate that PreTSA produces results highly consistent with those of existing methods while substantially increasing computational efficiency.

The top TVGs identified by PreTSA-K capture key biological functions underlying the cell development process. For instance, many enriched GO terms are associated with memory T cell activation, such as MHC protein complex assembly and response to interleukin-7 (Fig. 3f). Among the top 20 TVGs (Additional file 1: Fig. S10), *S100A4* exhibits an increasing temporal trend, consistent with its role in modulating T cell migration [33] (Fig. 3 g). In contrast, the ribosomal gene *RPL32* shows a decreasing trend, reflecting reduced ribosome biogenesis during the transition to a quiescent memory state [34] (Fig. 3 h).

**Fig. 4:**
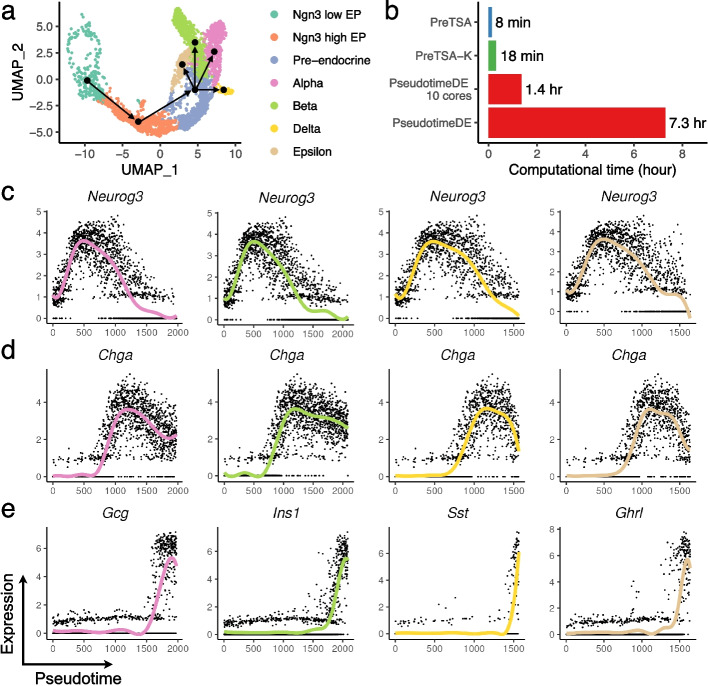
Analysis of the mouse pancreas scRNA-seq dataset. **a** UMAP plot colored by cell types. Lines indicate schematic directions of pseudotime trajectories for visualization. **b** Computational time of different methods for testing TVGs in the beta branch. **c-e** Expression (y-axis) of *Neurog3* (**c**), *Chga* (**d**), and branch-specific marker genes (**e**) along pseudotime (x-axis). Dots represent single-cell gene expression values, and curves indicate the fitted trajectories obtained by PreTSA-K. Curve colors correspond to the cell-type colors in panel **a**

To demonstrate that PreTSA can be applied to complex topologies, including branching structures, we applied it to study endocrine development in the mouse pancreas [35]. The dataset contains four endocrine lineages, alpha, beta, delta, and epsilon cell differentiation (Fig. 4a). In the beta cell lineage, which contains the largest number of cells among the four, PreTSA and PreTSA-K completed the analysis in 8 and 18 minutes, respectively, whereas PseudotimeDE required 7.3 hours on a single core and 1.4 hours on 10 cores, while also using more memory (Fig. 4b, Additional file 1: Fig. S11). Consistent with known endocrine development, GO terms enriched among the TVGs identified by PreTSA-K include insulin metabolic process and signal peptide processing (Additional file 1: Fig. S12-15). The top TVGs identified by PreTSA-K include both shared genes and branch-specific markers (Fig. 4c-e, Additional file 1: Fig. S16). For example, *Neurog3* initially increases as the master transcription factor initiating endocrine commitment, but subsequently decreases once its transient role is fulfilled as cells progress into the pre-endocrine state [35, 36] (Fig. 4c). *Chga*, which is involved in hormone processing, increases during endocrine differentiation and then slightly decreases as endocrine cells mature [35] (Fig. 4 d). In addition, canonical markers for each endocrine cell type exhibit increasing trends within their respective branches [37] (Fig. 4e).

**Fig. 5:**
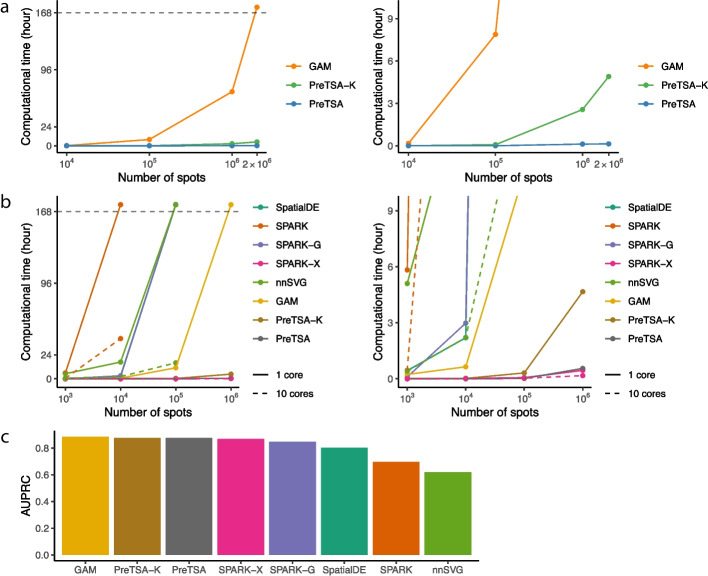
Simulation studies using the Visium human heart dataset. **a**,** b** Computational time (y-axes) of different methods for fitting the spatial pattern (**a**) and testing SVGs (**b**) in simulated data generated by sampling from the Visium human heart dataset with different numbers of spots (x-axes, log10 scale). Computational time more than one week (168 hours) is marked as timeout. For scenarios in which a method exceeded the 400 GB memory limit, its computational time could not be obtained and is therefore not shown in the plot. The right panels are zoom in views of the left panels. **c** Area under the precision-recall curve (AUPRC) for the simulation dataset generated by scDesign3, with methods ranked in decreasing order

PreTSA can also be applied to ST data by extending the one-dimensional B-spline bases to two dimensions using tensor products (Fig. 1b). Since the spatial locations of cells are directly measured, PreTSA uses an F-test to identify SVGs, without accounting for additional variance as in the case of identifying TVGs. Similarly, GAM can be extended with tensor products (Methods). Using a real 10x Visium dataset of the human heart, we constructed a simulated dataset with 11,953 genes and thousands to millions of spatial spots. Note that although the number of spots in 10x Visium datasets is relatively small, the latest ST technologies, such as 10x Visium HD, 10x Xenium, high-definition spatial transcriptomics (HDST) [38], MERSCOPE, and NanoString CosMx, can easily produce a much larger number of spots or cells. Thus, the simulated dataset reflects the different scenarios one may encounter in practice. PreTSA again demonstrates superior computational efficiency over GAM (Fig. 5a). For analyzing one million spots, GAM took 68.3 hours, while PreTSA required only 7 minutes and less memory (Additional file 1: Fig. S17). For analyzing two million spots, GAM failed to complete the computation within a week, while PreTSA took just 8 minutes. For identifying SVGs, PreTSA and SPARK-X are the only methods that can handle a million spots within an hour (Fig. 5b). Methods such as SpatialDE, SPARK, SPARK-G, and nnSVG failed to complete the computation within a week even for a hundred thousand spots, and GAM was unable to handle a million spots within a week. Note that SPARK, SPARK-X, and nnSVG provide parallelization options. Although parallelization substantially reduces computation time, SPARK and nnSVG still require considerably more time than PreTSA and PreTSA-K. Moreover, their memory usage increases markedly with parallelization, preventing them from handling datasets with 100,000 and 1,000,000 spots, respectively, under a 400 GB memory constraint (Additional file 1: Fig. S18). Similar to the case of TVG identification, PreTSA achieved one of the highest AUPRCs for SVG identification on an scDesign3 simulation dataset (Fig. 5c) and detected almost no SVGs on a null simulation dataset (Additional file 1: Fig. S19).

**Fig. 6:**
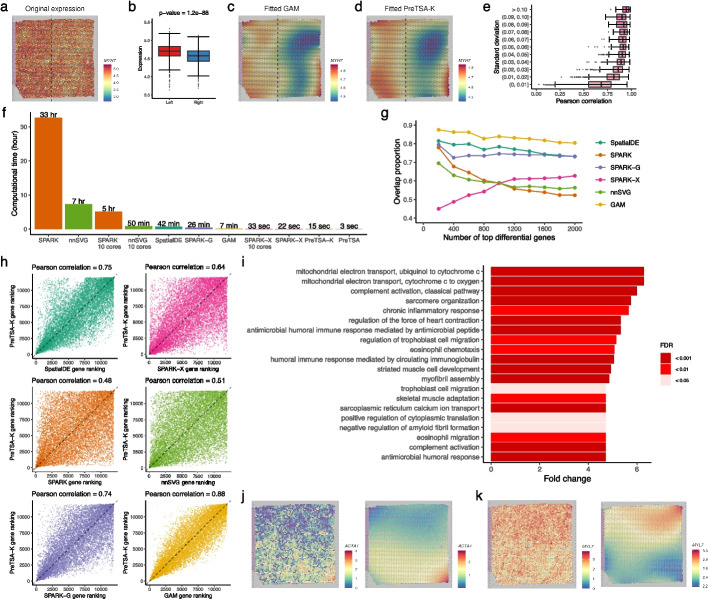
Analysis of the original Visium human heart dataset. **a** Original expression of *MYH7*. **b** Boxplot showing the distribution of *MYH7* expression in the left and right spatial regions, separated by the dotted vertical line in **a**. *p*-value is obtained by Wilcoxon rank-sum test. **c**,** d** Spatial expression pattern of *MYH7* fitted by GAM (**c**) and by PreTSA-K (**d**). **e** Pearson correlations between fitted values by GAM and by PreTSA-K (x-axis), grouped by the standard deviation of fitted values by GAM (y-axis). **f** Computational time of different methods for testing SVGs. **g** Overlap proportion for different numbers of top differential genes using PreTSA-K and other methods. **h** Gene rankings by PreTSA-K (y-axis) and by other methods (x-axis). **i** Top GO terms enriched among SVGs identified by PreTSA-K. **j**,** k** Original expression (left) and PreTSA-K-fitted expression (right) of *ACTA1* (**j**) and *MYL7* (**k**)

While PreTSA and SPARK-X have comparable computational efficiency, SPARK-X lacks the ability to fit the spatial pattern of gene expression, which is crucial for understanding complex ST data. Figure 6a shows the expression of the example gene *MYH7* in the original Visium heart dataset. *MYH7* plays a critical role in the functionality of cardiac muscle fibers in the human heart [39]. Although it is challenging to visually discern the spatial pattern of the gene, the expression level of *MYH7* is significantly higher in the left part of the tissue compared to the right (Fig. 6b). This distinction can only be elucidated by the spatial gene expression patterns fitted by GAM and PreTSA-K, which are highly similar (Fig. 6c, d). The median PCC between surfaces fitted by PreTSA-K and GAM exceeds 0.85 for genes with standard deviations of GAM-fitted surfaces larger than 0.01 (Fig. 6e). PreTSA demonstrates highly similar findings (Additional file 1: Fig. S20a-b). These results indicate that PreTSA is the only method capable of efficiently fitting spatial gene expression patterns in large datasets.

We further compared the results of identifying SVGs using PreTSA and other existing methods in the original Visium heart dataset. Both PreTSA and SPARK-X completed computations within a minute, whereas methods such as SPARK and nnSVG required several hours (Fig. 6f, Additional file 1: Fig. S21). Genes were ordered increasingly by the *p*-values from different methods for identifying SVGs. The top-ranked genes identified by PreTSA-K show the most similarity to those identified by GAM, SpatialDE, and SPARK-G, with an overlap proportion of around 80% (Fig. 6 g). The Pearson correlation coefficients for the overall gene rankings also reached at least 0.74 (Fig. 6 h). The results from PreTSA-K are less consistent with those from SPARK, SPARK-X, and nnSVG. However, SPARK, SPARK-X, and nnSVG are more distinct from any other existing methods, whereas GAM, SpatialDE, and SPARK-G are more similar to each other (Additional file 1: Fig. S20c). PreTSA demonstrated highly similar results (Additional file 1: Fig. S20d-e). While it is hard to assess the quality of identified SVGs due to the lack of a gold standard of SVGs in real datasets [40, 41], these results suggest that PreTSA is consistent with a group of existing methods for identifying SVGs. Consistent with cardiac function, GO terms enriched among the SVGs identified by PreTSA-K include sarcomere organization, regulation of cardiac contraction force, and myofibril assembly (Fig. 6i). Notably, *ACTA1* shows elevated expression in the bottom-right region of the tissue, suggesting cardiac remodeling [42] (Fig. 6j), whereas *MYL7* is enriched in the top-right region, likely corresponding to atrial cardiomyocytes [43] (Fig. 6k, Additional file 1: Figs. S22-23).

**Fig. 7:**
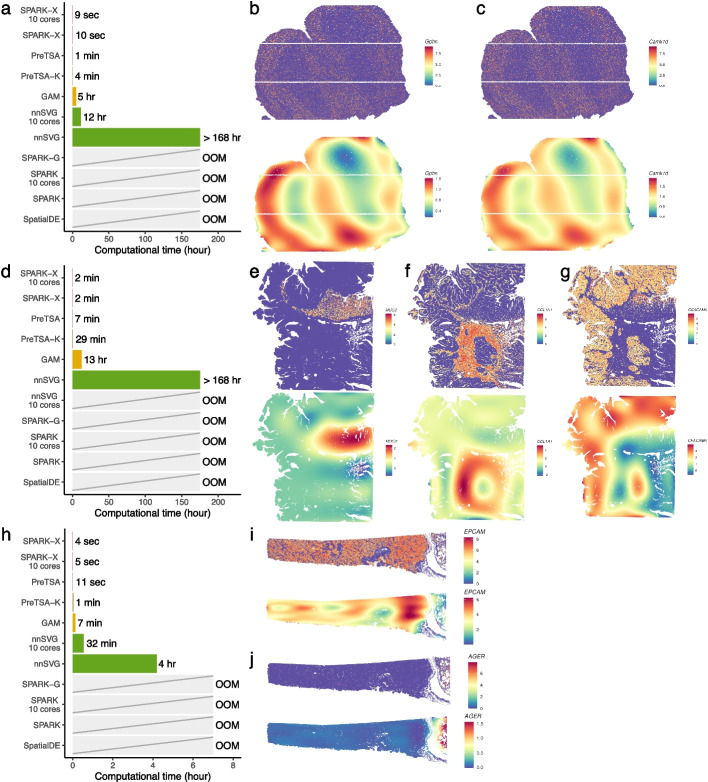
**a** Computational time of different methods for testing SVGs in the HDST mouse olfactory bulb dataset. **b-c** Original expression (top) and PreTSA-K-fitted expression (bottom) of *Gphn* (**b**) and *Camk1d* (**c**) in the HDST mouse olfactory bulb dataset. **d** Computational time of different methods for testing SVGs in the Visium HD human colon cancer dataset. **e-g** Original expression (top) and PreTSA-K-fitted expression (bottom) of *MUC2* (**e**), *COL1A1* (**f**), and *CEACAM6* (**g**) in the Visium HD human colon cancer dataset. **h** Computational time of different methods for testing SVGs in the Xenium human lung cancer dataset. **i**,** j** Original expression (top) and PreTSA-K-fitted expression (bottom) of *EPCAM* (**i**) and *AGER* (**j**) in the Xenium human lung cancer dataset

To further demonstrate the generality of PreTSA across different ST technologies, we applied it to a mouse olfactory bulb dataset generated by HDST [38]. After filtering, the dataset contained 10,729 genes and 181,135 spots. PreTSA, PreTSA-K, and SPARK-X each completed the analysis within 4 minutes, whereas other methods either required much longer runtimes or failed due to excessive memory usage (Fig. 7a, Additional file 1: Fig. S24). The SVGs identified by PreTSA-K were enriched in key biological processes of the olfactory bulb, including ionotropic glutamate receptor signaling and SNARE complex assembly (Additional file 1: Fig. S25). The fitted spatial expression patterns of top-ranked SVGs showed pronounced spatial organization consistent with known biology (Additional file 1: Figs. S26-27). For example, *Gphn* and *Camk1d*, which are critical for inhibitory synapse organization and calcium signaling, respectively, showed elevated expression in the external plexiform layer and glomerular layer [44, 45] (Fig. 7b, c).

Next, we applied PreTSA to identify SVGs in a human colorectal cancer (CRC) sample generated by the newly developed 10x Visium HD platform [46]. After filtering, the dataset contained 9,621 genes and 419,309 spots. PreTSA, PreTSA-K, and SPARK-X each completed the analysis within 30 minutes, whereas other methods either required substantially longer runtimes or failed due to excessive memory usage (Fig. 7 d, Additional file 1: Fig. S28). The SVGs identified by PreTSA-K were enriched in pathways such as antimicrobial humoral response and collagen fibril organization, reflecting the distinct epithelial and stromal programs characteristic of colon cancer tissue (Additional file 1: Fig. S29). Consistent with these pathways, many of the top SVGs are well-established tumor-associated genes (Additional file 1: Figs. S30-31). For example, *MUC2*, which contributes to mucus barrier formation, showed higher expression in the epithelial goblet cell compartment [47] (Fig. 7e); *COL1A1*, encoding fibrillar collagens secreted by cancer-associated fibroblasts, was upregulated in the stromal compartment [48] (Fig. 7f); and *CEACAM6*, a tumor-associated adhesion molecule, was enriched in malignant glands [49] (Fig. 7 g).

Finally, we applied PreTSA to identify SVGs in a human lung cancer sample generated using 10x Xenium. Unlike the previous examples based on spot-based platforms, 10x Xenium represents an image-based platform. After filtering, the dataset contained 377 genes and 151,677 cells. PreTSA, PreTSA-K, and SPARK-X each completed the analysis within one minute, whereas other methods either required substantially longer runtimes or failed due to excessive memory usage (Fig. 7 h, Additional file 1: Fig. S32). Notably, many top-ranked SVGs identified by PreTSA-K showed clear spatial localization patterns consistent with known lung cancer tissue architecture (Additional file 1: Figs. S33-34). For example, *EPCAM*, which encodes the epithelial cell adhesion molecule, exhibited higher expression in malignant epithelial regions [50] (Fig. 7i). In contrast, *AGER*, which plays a critical role in pulmonary regulation, was predominantly expressed in the alveolar epithelium [51] (Fig. 7j).

## Discussion

The computational efficiency of trajectory inference methods, which provide the cell ordering required for TVG analysis, can vary greatly [6]. Although beyond the scope of this study, this variability should be considered when evaluating the total time needed to characterize gene expression dynamics from a raw expression matrix.

Spatial data can be analyzed at multiple scales, and PreTSA is agnostic to absolute units, operating over a user defined analysis domain. When applied once to an entire sample, PreTSA primarily captures tissue scale trends, which can attenuate or obscure fine scale variation at micrometer to tens of micrometers. Although the method does not assume a specific tissue size, cell number, or spatial transcriptomics resolution, detection of features at scales much smaller than the analysis domain is beyond the scope of a single global fit. Subsetting the tissue and applying PreTSA within a localized region might mitigate global artifacts and enable focused analyses, however this approach does not fully eliminate the inherent limitation regarding very small scale features.

In modeling spatial gene expression, PreTSA employs a tensor product of B-spline bases rather than the full polynomial basis, which may introduce an artifact whereby not all directions are treated equivalently. To assess the impact of this limitation, we rotated the spatial coordinates of spots in each spatial data by various angles and applied PreTSA. The comparison of fitted gene expression values and gene rankings between the original and rotated data showed generally high consistency (Additional file 1: Fig. S35), suggesting that this artifact has a mild impact on gene expression fitting and SVG identification.

Although the normality assumption in PreTSA deviates from the empirical distribution of log-transformed gene expression data due to dropouts [52], the method nonetheless achieves high accuracy in identifying TVGs and SVGs while effectively controlling false positives. Incorporating more complex distributions, such as zero-inflated models, could improve statistical rigor but would substantially increase computational complexity by eliminating the closed-form solution. Future research may focus on developing approaches that better balance distributional fidelity with computational efficiency.

PreTSA, like other existing approaches for identifying SVGs, is grounded in statistical modeling. In parallel, a number of deep learning-based methods have been developed for analyzing ST data, but their primary focus has been on tasks such as spatial domain detection rather than SVG identification. Examples include SpaGCN [53], SpaSEG [54], DeepGFT [55], and SPACEL [56], which are effective for uncovering tissue architecture and domain-specific expression patterns but are not designed for detecting SVGs. While deep learning methods are often praised for their scalability and efficiency in handling large-scale data, it remains unknown whether such approaches can be successfully adapted to the task of SVG identification. Exploring this possibility represents an interesting avenue for future research.

In this study, PreTSA was tested using a standard installation of R. For users with suitable hardware and technical expertise, non-standard R installations may further increase the computational efficiency of PreTSA. For example, using a threaded MKL-based R installation, such as one installed via a package manager like conda, may reduce computation time by more efficiently parallelizing matrix operations. Moreover, although PreTSA was implemented in R for compatibility with existing packages for single-cell and spatial analysis such as Seurat [57], Monocle [1], and TSCAN [2], Python and other programming languages may be more suitable for parallelization or achieving maximum computational efficiency. A Python implementation of PreTSA, which shows slightly reduced running time compared to the R version (Additional file 1: Fig. S36), is also provided in the PreTSA software package. Finally, PreTSA provides an optional binning strategy that averages gene expression values across cells within small spatial and temporal bins before fitting the model (Methods). This approach further reduces running time and memory usage (Additional file 1: Fig. S37) while producing fitted gene rankings similar to those obtained without binning (Additional file 1: Fig. S38), although it may reduce the spatial resolution of the original data. Thus, we recommend its use primarily when runtime or memory demands on the original data become prohibitive.

## Conclusions

In summary, we have demonstrated that PreTSA substantially improves computational efficiency in modeling temporal and spatial gene expression patterns, while producing results that are consistent with state-of-the-art methods, such as GAM, PseudotimeDE, and SpatialDE. PreTSA offers a unique solution for understanding the temporal and spatial dynamics of gene expression in large single-cell and ST datasets.

## Methods

### PreTSA model for temporal gene expression patterns

#### Inputs

PreTSA requires as input a numeric matrix of library-size-normalized and log-transformed gene expression values, along with a numeric vector of pseudotime values for single cells. The gene expression matrix can be derived from any standard processing pipeline for scRNA-seq data [57–59]. Pseudotime values can be obtained from any pseudotime inference method [1–5]. Note that for trajectories with multiple branches, PreTSA needs to be applied multiple times, each time to a single branch separately. Users also have the option to replace the pseudotime values with any other one-dimensional numeric values of interest.

#### Fitting temporal gene expression patterns

Denote $\textbf{Y}$ as the $m \times n$ gene expression matrix, with *m* genes and *n* cells, and $y_{ij}$ as the expression level of gene *i* in cell *j*. Denote $t_j$ as the pseudotime value for cell *j*. For each gene *i*, we model its expression values along pseudotime as a functional curve:

$$\begin{aligned} y_{ij} = \beta _{i0} + \sum \limits _{k=1}^{K+3} b_k(t_j)\beta _{ik} + \epsilon _{ij}\quad \quad \quad \epsilon _{ij} {\mathop {\sim }\limits ^{\text {iid}}} N\left( 0, \sigma _i^2\right) . \end{aligned}$$

Here, $b_1(t), \ldots , b_{K+3}(t)$ represent the $K+3$ cubic B-spline basis functions, where *K* is the number of equidistant internal knots used to define the cubic B-spline bases. The method for selecting *K* will be discussed later. The parameters $\beta _{i0}, \ldots , \beta _{i(K+3)}$ and $\sigma _i^2$ are all unknown and will be estimated using the least squares method.

For gene *i*, the regression model can be written in matrix form as follows: $\textbf{Y}_i = \textbf{X}\boldsymbol{\beta }_i + \boldsymbol{\epsilon }_i$, where

$$\begin{aligned} \textbf{X} =\left[ \begin{array}{cccc} 1 & b_1(t_1) & \cdots & b_{K+3}(t_1) \\ 1 & b_1(t_2) & \cdots & b_{K+3}(t_2) \\ \vdots & \vdots & \vdots & \vdots \\ 1 & b_1(t_n) & \cdots & b_{K+3}(t_n) \end{array}\right] . \end{aligned}$$

Note that the hat matrix $\textbf{H} = \textbf{X}(\textbf{X}^T \textbf{X})^{-1}\textbf{X}^T$ is shared across all genes. Consequently, we can apply the hat matrix directly to the original gene expression matrix to obtain the fitted gene expression matrix $\hat{\textbf{Y}}$ as follows:

$$\begin{aligned} \hat{\textbf{Y}} = \textbf{Y} \textbf{H} \end{aligned}$$

Here $\hat{y}_{ij}$ represents the fitted gene expression value of gene *i* in cell *j*.

PreTSA sets $K=0$ for all genes. PreTSA-K, an extended model of PreTSA, enables the automatic selection of *K* for each gene by minimizing the Bayesian Information Criterion (BIC). For a given *K* and a given gene *i*, $\text {BIC} = n\log (\frac{1}{n} \sum _{j=1}^n (y_{ij} - \hat{y}_{ij})^2) + (K+5)\log (n) + n + n\log (2\pi )$.

By default, PreTSA-K chooses *K* from the set of integers ranging from 0 to 10.

#### Testing for temporally variable genes (TVGs)

To test whether the expression level of gene *i* is temporally variable, or equivalently, cannot be represented by a horizontal line along the pseudotime axis, we define the following null and alternative hypotheses:

$$\begin{aligned} H_0: \beta _{ik}= & 0, \; \text {for all} \; k = 1, \ldots , K+3\\ H_1: \beta _{ik}\ne & 0, \; \text {for at least one} \; k = 1, \ldots , K+3 \end{aligned}$$

PreTSA uses the F-statistics:

$$\begin{aligned} F_i = \frac{\sum _{j=1}^n (\hat{y}_{ij} - \bar{y}_{i})^2 / (K+3) }{\sum _{j=1}^n (y_{ij} - \hat{y}_{ij})^2 / (n-K-4)} \end{aligned}$$

Here $$\bar{y}_{i} = \frac{1}{n} \sum _{j=1}^n y_{ij}$$.

To account for the uncertainty inherent in computationally inferring pseudotime, we apply the same strategy used in PseudotimeDE [21]. We first subsample 80% of the cells, a proportion suggested by PseudotimeDE [21], and then apply the same pseudotime inference method that was used for the original dataset to this subsampled dataset to obtain the pseudotime values. These values are subsequently randomly permuted. Following this, the fitting approach described earlier is applied to the subsampled and permuted dataset to obtain null F-statistics. This entire process is repeated 100 times to generate 100 null F-statistics. To enhance numerical accuracy, a Gamma distribution is fitted to these 100 null F-statistics using the R package fitdistrplus (version 1.1.11). The *p*-value is calculated as the probability of the tail of the fitted Gamma distribution being greater than $F_i$. All *p*-values are then adjusted for multiple testing using the Benjamini-Hochberg (BH) procedure [60].

PreTSA can also identify TVGs using the F-test to further improve computational efficiency, although statistical rigor is not guaranteed. Assuming fixed pseudotime values, the F-statistic introduced above follows an exact F-distribution with $(K+3, n-K-4)$ degrees of freedom under $H_0$.

### PreTSA model for spatial gene expression patterns

#### Inputs

PreTSA requires two inputs: a numeric matrix of library-size-normalized and log-transformed gene expression values, and a numeric matrix of 2-dimensional spatial locations for cells or spatial spots. The gene expression matrix can be obtained through any standard processing pipeline for ST data [57, 61, 62]. The 2-dimensional spatial location information is usually directly available from the ST dataset. Users can subset the ST data to focus on specific tissue regions, enable multiscale spatial analysis, or mitigate technical issues such as imaging artifacts.

#### Fitting spatial gene expression patterns

Denote $\textbf{Y}$ as the $m \times n$ gene expression matrix, with *m* representing number of genes and *n* representing number of cells or spots, and $y_{ij}$ as the expression level of gene *i* in cell or spot *j*. Denote $\textbf{S}_j = (s_{j1}, s_{j2})$ as the 2-dimensional spatial location of cell or spot *j*.

For each gene *i*, we define a smooth surface over spatial locations using a tensor product of univariate B-spline basis functions along each spatial axis:

$$\begin{aligned} & y_{ij} = f_i(s_{j1}, s_{j2}) + \epsilon _{ij}, \quad \epsilon _{ij} \overset{\text {iid}}{\sim } N\left(0, \sigma _i^2\right),\\ & f_i(s_{j1}, s_{j2}) = \sum \limits _{k_1=1}^{K+3} \sum \limits _{k_2=1}^{K+3} \beta _{i,k_1,k_2} \, b_{1,k_1}(s_{j1}) \, b_{2,k_2}(s_{j2}) + \sum \limits _{k_1=1}^{K+3} \beta _{i,k_1,0} \, b_{1,k_1}(s_{j1}) + \sum \limits _{k_2=1}^{K+3} \beta _{i,0,k_2} \, b_{2,k_2}(s_{j2}) + \beta _{i0}. \end{aligned}$$

In this model, $b_{1,k}(s)$ and $b_{2,k}(s)$ denote the *k*th cubic B-spline basis functions for the first and second spatial dimensions, respectively. Each dimension includes $K+3$ basis functions, assuming *K* internal knots. The term $b_{1,k_1}(s_{j1}) \, b_{2,k_2}(s_{j2})$ defines the tensor product basis, capturing interactions between the two spatial axes. The additive terms $\beta _{i,k_1,0} \, b_{1,k_1}(s_{j1})$ and $\beta _{i,0,k_2} \, b_{2,k_2}(s_{j2})$ represent marginal effects along each axis, and the scalar $\beta _{i0}$ serves as the intercept. The full set of parameters, including the interaction coefficients $\beta _{i,k_1,k_2}$, the marginal coefficients $\beta _{i,k_1,0}$ and $\beta _{i,0,k_2}$, the intercept $\beta _{i0}$, and the variance $\sigma _i^2$, are specific to each gene and estimated via least squares. This formulation yields a flexible and smooth spatial surface for gene expression, capturing both individual axis effects and their interactions across the two-dimensional tissue landscape. Note that this formulation breaks the rotational symmetry of the 2D space into a discrete subgroup, meaning that not all directions are treated equivalently and the model cannot guarantee identical fits for data rotated by, for example, $45^\circ$. See the Discussion section for a more detailed discussion.

For gene *i*, the regression model can be written in matrix form as follows: $\textbf{Y}_i = \textbf{X}\boldsymbol{\beta }_i + \boldsymbol{\epsilon }_i$, where

$$\begin{aligned} \textbf{X} = \left[ \begin{array}{cccccccccc} 1 & b_{1, 1}(s_{11}) & \cdots & b_{1, K+3}(s_{11}) & b_{2, 1}(s_{12}) & \cdots & b_{2, K+3}(s_{12}) & b_{1, 1}(s_{11}) b_{2, 1}(s_{12}) & \cdots & b_{1, K+3}(s_{11}) b_{2, K+3}(s_{12}) \\ 1 & b_{1, 1}(s_{21}) & \cdots & b_{1, K+3}(s_{21}) & b_{2, 1}(s_{22}) & \cdots & b_{2, K+3}(s_{22}) & b_{1, 1}(s_{21}) b_{2, 1}(s_{22}) & \cdots & b_{1, K+3}(s_{21}) b_{2, K+3}(s_{22}) \\ \vdots & \vdots & \vdots & \vdots \\ 1 & b_{1, 1}(s_{n1}) & \cdots & b_{1, K+3}(s_{n1}) & b_{2, 1}(s_{n2}) & \cdots & b_{2, K+3}(s_{n2}) & b_{1, 1}(s_{n1}) b_{2, 1}(s_{n2}) & \cdots & b_{1, K+3}(s_{n1}) b_{2, K+3}(s_{n2}) \end{array}\right] . \end{aligned}$$

Similar to the case of modeling the temporal gene expression pattern, we can directly apply the hat matrix to the original gene expression matrix to obtain the fitted gene expression matrix $\hat{\textbf{Y}}$:

$$\begin{aligned} \hat{\textbf{Y}} = \textbf{Y} \textbf{H} \end{aligned}$$

Here $\hat{y}_{ij}$ represents the fitted gene expression value of gene *i* in cell or spot *j*.

PreTSA chooses $K=0$ for all genes. PreTSA-K chooses the optimal *K* using the same BIC procedure as the case of fitting temporal gene expression pattern. For a given *K* and a given gene *i*, $\text {BIC} = n\log \left(\frac{1}{n} \sum _{j=1}^n (y_{ij} - \hat{y}_{ij})^2\right) + ((K+4)^2 + 1)\log (n) + n + n\log (2\pi )$.

By default, PreTSA-K chooses *K* from the set of integers ranging from 0 to 5.

#### Testing for spatially variable genes (SVGs)

To test whether the expression level of gene *i* varies spatially, similar to the case of testing for TVGs, we define the following null and alternative hypotheses:

$$\begin{aligned} & H_0: \beta _{i,1,0} = \ldots = \beta _{i,K+3,0} = \beta _{i,0,1} = \ldots = \beta _{i,0,K+3} = \beta _{i,1,1} = \ldots = \beta _{i,K+3,K+3} = 0\\ & H_1: \text {At least one of the parameters in} \; H_0 \; \text {is not zero} \end{aligned}$$

PreTSA uses the F-statistics:

$$\begin{aligned} F_i = \frac{\sum _{j=1}^n (\hat{y}_{ij} - \bar{y}_{i})^2 / ((K+4)^2 - 1) }{\sum _{j=1}^n (y_{ij} - \hat{y}_{ij})^2 / (n - (K+4)^2) } \end{aligned}$$

which follows an exact F-distribution with $((K+4)^2 - 1, n - (K+4)^2)$ degrees of freedom under $H_0$.

Here $\bar{y}_{i} = \frac{1}{n} \sum _{j=1}^n y_{ij}$. All *p*-values are then adjusted for multiple testing using the BH procedure [60].

### Optional temporal and spatial binning

PreTSA provides an optional binning strategy that partitions pseudotime values into 100 equal-width intervals or spatial locations into an evenly spaced 100 $\times$ 100 grid, with gene expression averaged within each temporal or spatial bin. PreTSA or PreTSA-K then fits temporal or spatial gene expression patterns using the binned gene expression values.

### GO enrichment analysis

GO enrichment analysis was performed using the R package topGO (version 2.56.0). When more than 10% of genes were identified as differential TVGs or SVGs, only the top 10% were used for enrichment analysis, otherwise all differential genes were included. All *p*-values were adjusted for multiple testing using the BH procedure to obtain FDRs [60], and GO terms with an FDR $\le 0.05$ were retained and ranked in decreasing order of fold change.

### Data collection and processing

#### Human PBMC scRNA-seq dataset

The processed dataset (Additional file 1: Fig. S39) was downloaded using the R package SeuratData (version 0.2.2). It included the log-normalized gene expression matrix, principal components (PCs) and cell type annotations. TSCAN [2] (version 2.0.0) was performed using the top 10 PCs linking naive CD4 T cells and memory CD4 T cells, using the R function guided_tscan(). The direction of the pseudotime is from naive CD4 T cells to memory CD4 T cells. Genes that are expressed in fewer than 5 cells within the pseudotime trajectory were filtered out. A total of 10,509 genes and 1,180 cells were obtained.

#### Mouse pancreas scRNA-seq dataset

The processed dataset (Additional file 1: Fig. S40) was obtained from the scVelo package (version 0.3.3) and included the gene expression count matrix, principal components (PCs), and cell type annotations. TSCAN [2] (version 2.0.0) was applied to the top 10 PCs to reconstruct trajectories linking cells involved in pancreatic endocrine cell development (alpha, beta, delta, and epsilon lineages), using the R function guided_tscan(). For each lineage, the pseudotime was oriented from “Ngn3 low EP” through “Ngn3 high EP” and “Pre-endocrine” to the corresponding terminal endocrine cell type (“Alpha”, “Beta”, “Delta”, or “Epsilon”). Genes expressed in fewer than five cells along the trajectory were filtered out, resulting in 13,709 genes and 1,977 cells for the alpha lineage, 13,755 genes and 2,087 cells for the beta lineage, 13,176 genes and 1,566 cells for the delta lineage, and 13,344 genes and 1,638 for the epsilon lineage. The log-normalized gene expression matrix was then obtained using the NormalizeData function in Seurat with default settings.

#### Visium human heart dataset

The dataset (Additional file 1: Fig. S41) was downloaded from the 10x website https://www.10xgenomics.com/resources/datasets/human-heart-1-standard-1-1-0. Mitochondrial genes and genes expressed in fewer than 50 spots were filtered out. A total of 11,953 genes and 4,247 spots were obtained. SCTransform [63] (version 0.4.1) with default settings was used to normalize and log-transform the raw count data.

#### HDST mouse olfactory bulb dataset

The processed dataset (Additional file 1: Fig. S42) was downloaded from https://github.com/xzhoulab/SPARK-X-Analysis/tree/main/processed_data. Genes expressed in fewer than 10 spots were filtered out, and only spots with positive expression for at least one retained gene were kept, resulting in 10,729 genes and 181,135 spots. The log-normalized gene expression matrix was then obtained using the NormalizeData function in Seurat with default settings.

#### Visium HD human colon cancer dataset

The dataset (Additional file 1: Fig. S43) was downloaded from the 10x website https://www.10xgenomics.com/datasets/visium-hd-cytassist-gene-expression-libraries-of-human-crc. The gene expression count matrix was generated from 8$\mu$m binned spots, and only spots with positive expression in at least 100 genes were retained. Mitochondrial genes and genes expressed in fewer than 1% of retained spots were removed, resulting in 9,621 genes and 419,309 spots. The log-normalized gene expression matrix was then obtained using the NormalizeData function in Seurat with default settings.

#### Xenium human lung cancer dataset

The dataset (Additional file 1: Fig. S44) was downloaded from the 10x website https://www.10xgenomics.com/datasets/preview-data-ffpe-human-lung-cancer-with-xenium-multimodal-cell-segmentation-1-standard. The gene expression count matrix was obtained from 10x Xenium Ranger software, and only cells with positive expression in at least 10 genes were retained, resulting in 377 genes and 151,677 cells. The log-normalized gene expression matrix was then obtained using the NormalizeData function in Seurat with default settings.

### Sensitivity analysis of the number of internal knots

For the human PBMC scRNA-seq dataset, PreTSA was applied with internal knots ranging from 0 to 10. For the Visium human heart dataset, PreTSA was applied with internal knots ranging from 0 to 5. Pearson correlation coefficients were then calculated between the fitted values and the overall gene rankings obtained under different numbers of internal knots.

### Sensitivity analysis of tissue rotation

For each spatial dataset, the spatial coordinates of spots were rotated by various angles, and Pearson correlation coefficients were calculated between the fitted values and overall gene rankings obtained by PreTSA or PreTSA-K from the original and rotated datasets.

### Simulation datasets

#### Simulation datasets via sampling

For human PBMC scRNA-seq dataset, we randomly sampled 1 thousand, 10 thousand, 100 thousand, 1 million, and 3 million cells with replacement to create simulated datasets. The gene expression profiles of these cells are the same as in the original dataset. The pseudotime values of these cells are assigned as a sequence of consecutive integers, starting from one and continuing through to the total number of cells.

For Visium human heart dataset, we randomly sampled 1 thousand, 10 thousand, 100 thousand, 1 million, and 2 million spots with replacement to create simulated datasets. The gene expression profiles of these spots are the same as in the original dataset. The spatial locations of these spots are assigned as all possible combinations of two sequences of consecutive integers. The first sequence ranges from 1 to *i*, and the second sequence ranges from 1 to *j*, for each pair of values (*i*, *j*) specified. In this simulation, the specific pairs used are (25, 40), (80, 125), (250, 400), (800, 1250), (1000, 2000), respectively.

To reduce memory usage when constructing the full gene expression matrix for extremely large datasets, both PreTSA and GAM divided the genes into 10 bins, generated fitted matrices for each bin, and then combined the results. This approach was applied to datasets with at least 1 million cells or spots.

#### Simulation datasets via scDesign3

scDesign3 (version 1.5.0) was used to generate synthetic data based on the human PBMC scRNA-seq and Visium human heart datasets. A total of 2,000 genes were randomly selected for simulation, with 10% designated as ground-truth TVGs/SVGs and the remaining as non-TVGs/SVGs. Different TVG and SVG identification methods were applied to the simulated gene expression matrix, and their performance was assessed using the area under the precision-recall curve (AUPRC), calculated with the pr.curve function from the PRROC package.

#### Null simulation datasets

For the human PBMC scRNA-seq dataset, cell pseudotime values were randomly permuted and methods for identifying TVGs were applied to the resulting null dataset. For the Visium human heart dataset, spatial coordinates of cells were randomly permuted and methods for identifying SVGs were applied to the null dataset. Genes with an FDR $\le 0.05$ were treated as false positives.

### Competing methods

For modeling temporal gene expression patterns, TSCAN-GAM was implemented using the R package mgcv (version 1.8.41) with the formula $y \sim s(t, k=3)$, same as TSCAN [2]. Monocle-GAM was implemented using the R package VGAM (version 1.1.13) with the formula $y \sim sm.ns(t, df=3)$, while the epsilon parameter was set to 0.1 and the family parameter was set to “uninormal”, same as Monocle [1]. PseudotimeDE [21] (version 1.0.0) with the R function runPseudotimeDE(model = “gaussian”) was used for testing TVGs.

For modeling spatial gene expression patterns, GAM was implemented using the R package mgcv (version 1.8.41) with the formula $y \sim te(s_{1}, s_{2})$, where $s_{1}$ and $s_{2}$ are vectors representing spatial locations in two dimensions, respectively. To test SVGs, GAM performs an asymptotic chi-squared likelihood ratio test where the full model fits the gene expression across spatial locations and the null model considers the gene expression as a constant across spatial locations. In addition, we applied SpatialDE [22] (version 1.1.3), SPARK [23] (version 1.1.1), SPARK-G [23] (version 1.1.1), SPARK-X [24] (version 1.1.1), and nnSVG [25] (version 1.5.8) for testing SVGs.

### Overlap proportion

To quantify the similarity of the top-ranked genes identified by different methods, overlap proportion is defined as the proportion of top-ranked genes identified by both methods. Specifically, denote *A* and *B* as the set of top *L* genes identified by two methods. $\vert A \vert = \vert B \vert = L$, where $\vert . \vert$ is the cardinality of a set. The overlap proportion is defined as $\frac{\vert A \cap B \vert }{L}$. The overlap proportion was calculated for different choices of *L*.

## Supplementary information

Additional file 1. Supplementary figures. This file contains Figs. S1-44.

Additional file 2. Peer review history.

## Data Availability

All datasets used in the study are publicly available. Human PBMC scRNA-seq dataset is available from 10x Genomics: https://www.10xgenomics.com/datasets/3-k-pbm-cs-from-a-healthy-donor-1-standard-1-1-0 [64]. Mouse pancreas scRNA-seq dataset is available from Gene Expression Omnibus under accession number GSE132188: https://www.ncbi.nlm.nih.gov/geo/query/acc.cgi?acc=GSE132188 [65]. Visium human heart dataset is available from 10x Genomics: https://www.10xgenomics.com/resources/datasets/human-heart-1-standard-1-1-0 [66]. HDST mouse olfactory bulb dataset is available from Gene Expression Omnibus under accession number GSE130682: https://www.ncbi.nlm.nih.gov/geo/query/acc.cgi?acc=GSE130682 [67]. Visium HD human colon cancer dataset is available from Gene Expression Omnibus under accession number GSE280318: https://www.ncbi.nlm.nih.gov/geo/query/acc.cgi?acc=GSE280318 [68]. Xenium human lung cancer dataset is available from 10x Genomics: https://www.10xgenomics.com/datasets/preview-data-ffpe-human-lung-cancer-with-xenium-multimodal-cell-segmentation-1-standard [69]. The R package PreTSA is publicly available at https://github.com/haotian-zhuang/PreTSA [70] and Zenodo [71] under the MIT license. Comprehensive tutorials are available at https://haotian-zhuang.github.io/PreTSA/. The source code to reproduce the results in this paper is available at https://github.com/haotian-zhuang/PreTSA_Paper and Zenodo [71] under the MIT license.
