# Supplementary material for: PreTSA: computationally efficient modeling of temporal and spatial gene expression patterns
Source: Genome Biol. 2026 Feb 12;27:93. doi: 10.1186/s13059-026-03994-3 (PMC12998178; doi:10.1186/s13059-026-03994-3)
Supplement: Supplementary file 1 — Additional file 1. Supplementary figures. This file contains Figs. S1-44. [file 13059_2026_3994_MOESM1_ESM.pdf]

Additional file 1

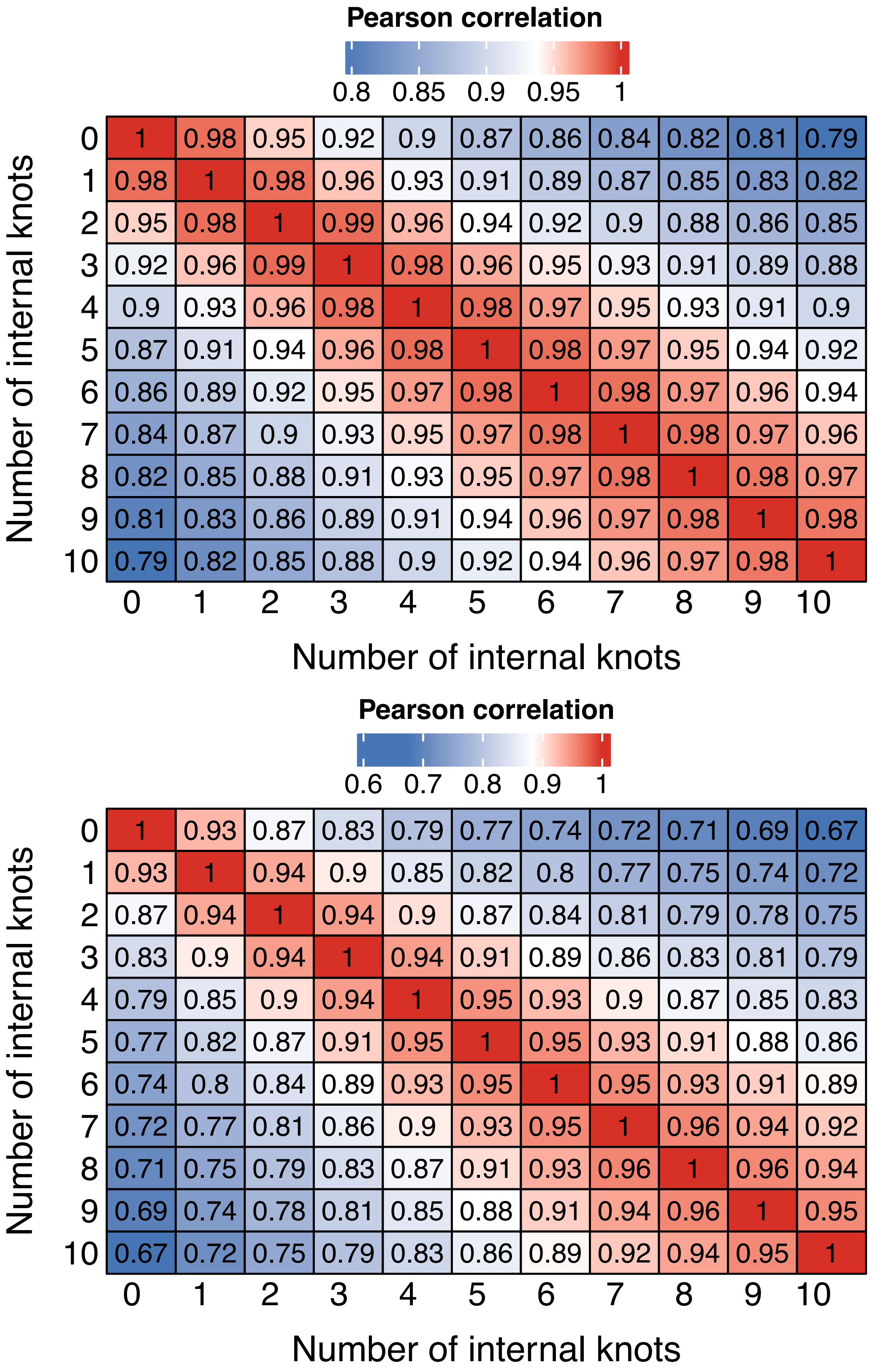

Fig. S1. Pearson correlations between fitted values (top) and overall gene rankings (bottom) obtained by applying `PreTSA` with varying numbers of internal knots to the human PBMC scRNA-seq dataset. Median correlations of fitted values were calculated using genes whose default `PreTSA`-fitted values had standard deviations greater than 0.05.

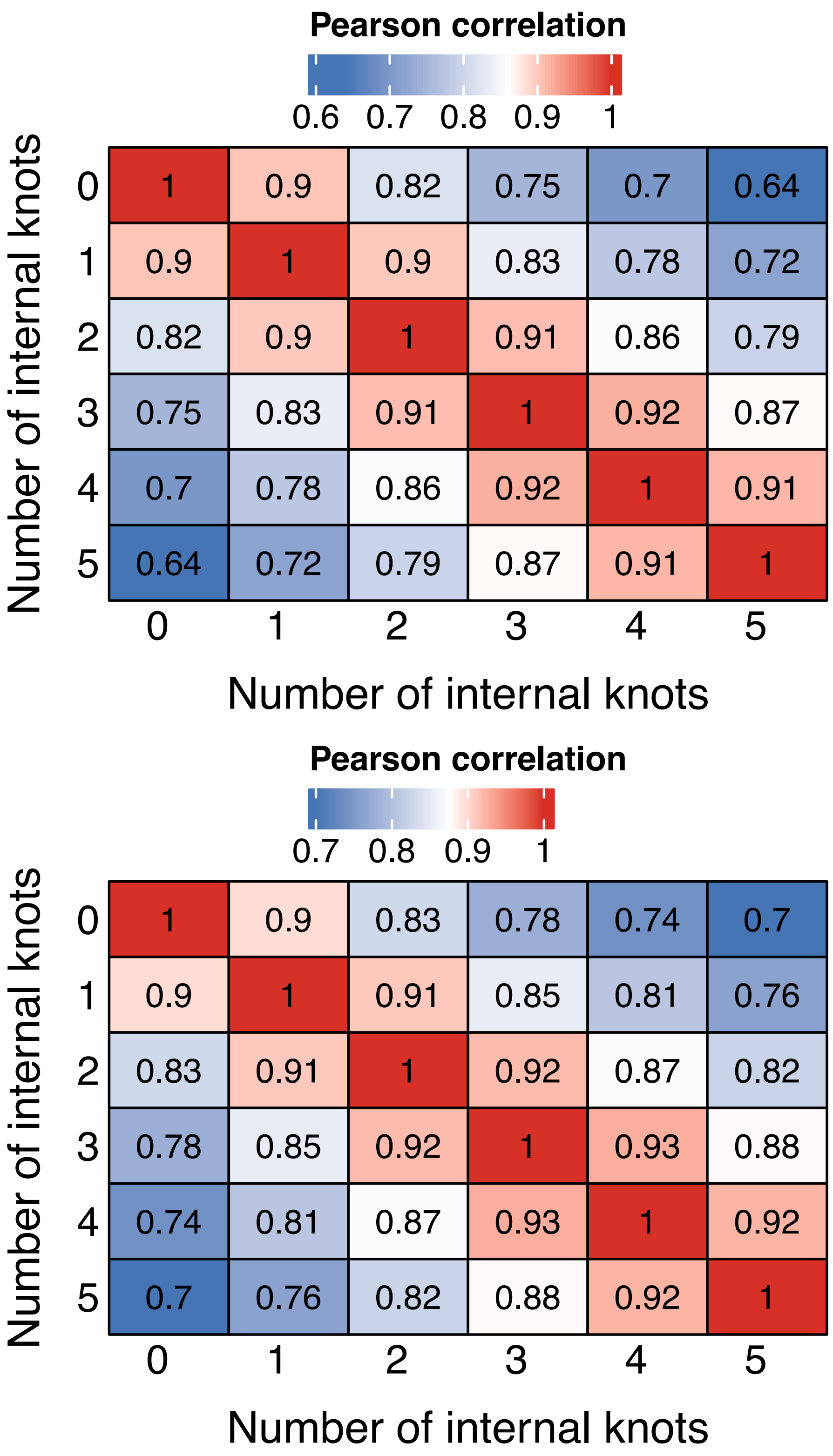

Fig. S2. Pearson correlations between fitted values (top) and overall gene rankings (bottom) obtained by applying `PreTSA` with varying numbers of internal knots to the Visium human heart dataset. Median correlations of fitted values were calculated using genes whose default `PreTSA`-fitted values had standard deviations greater than 0.05.

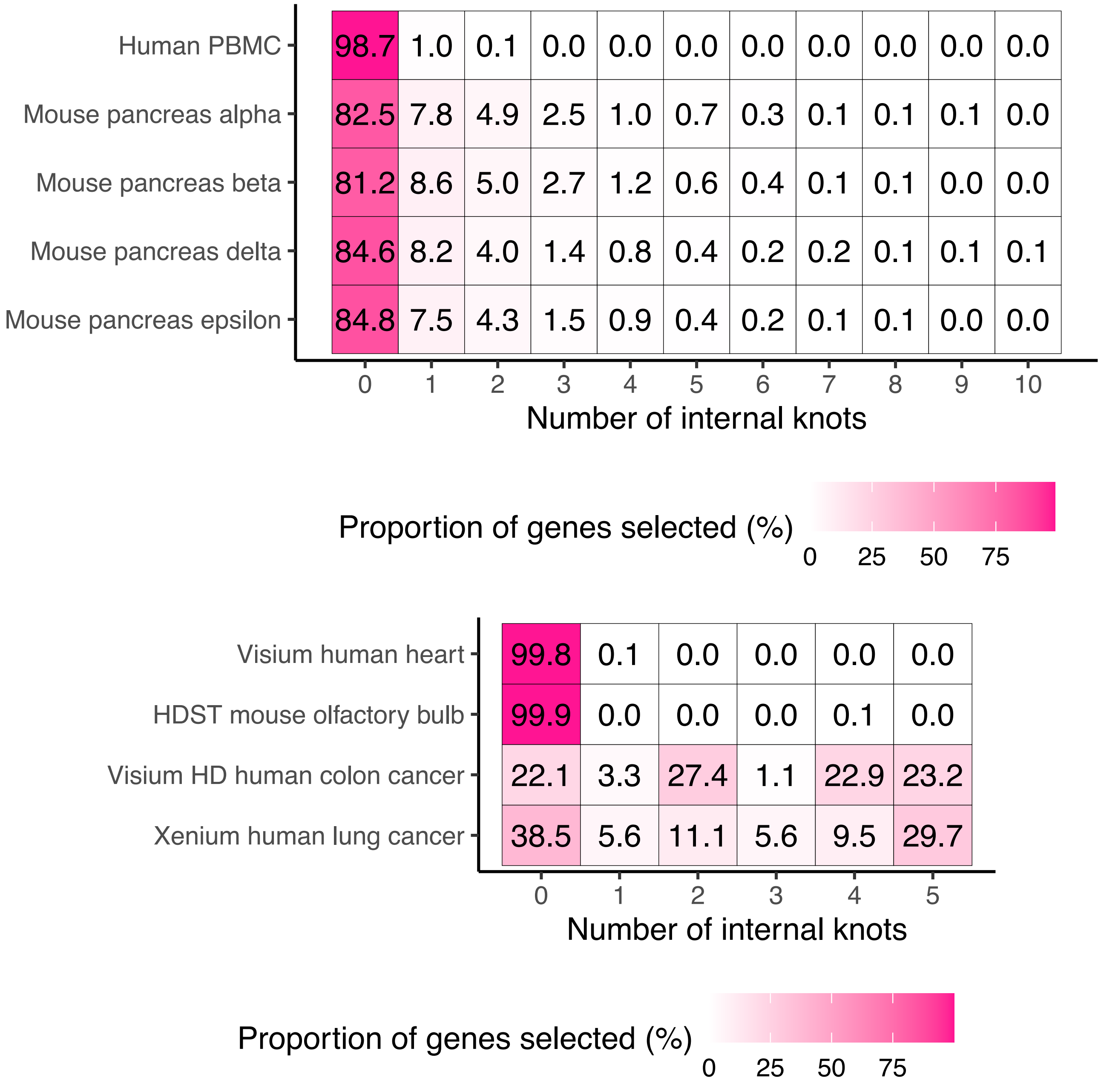

Fig. S3. Proportion of genes for which different numbers of internal knots were selected by `PreTSA-K` in each pseudotime (top) and spatial (bottom) dataset.

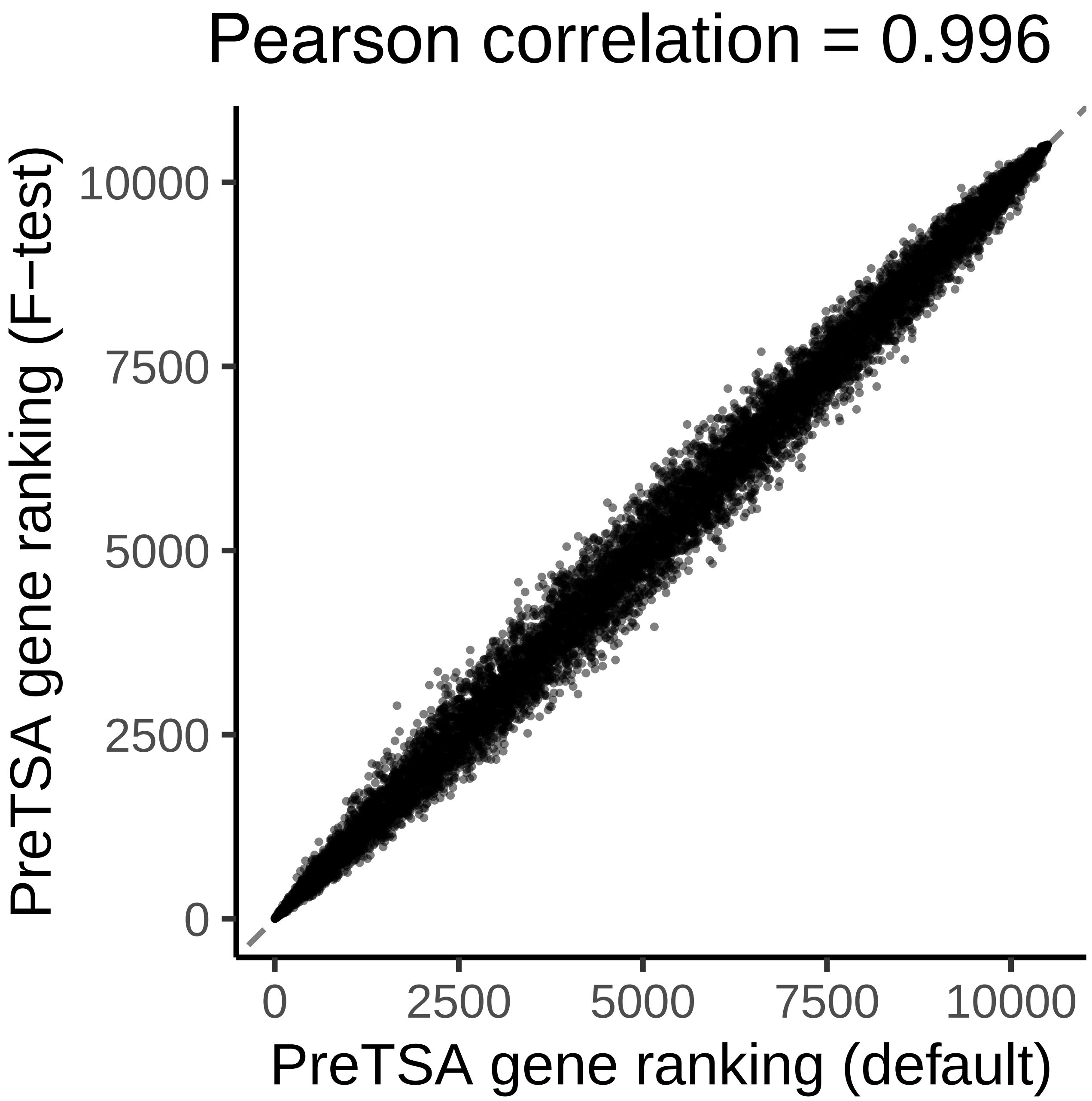

Fig. S4. Gene rankings for TVG identification in the human PBMC scRNA-seq dataset, with default `PreTSA` on the x-axis and `PreTSA` using the F-test on the y-axis.

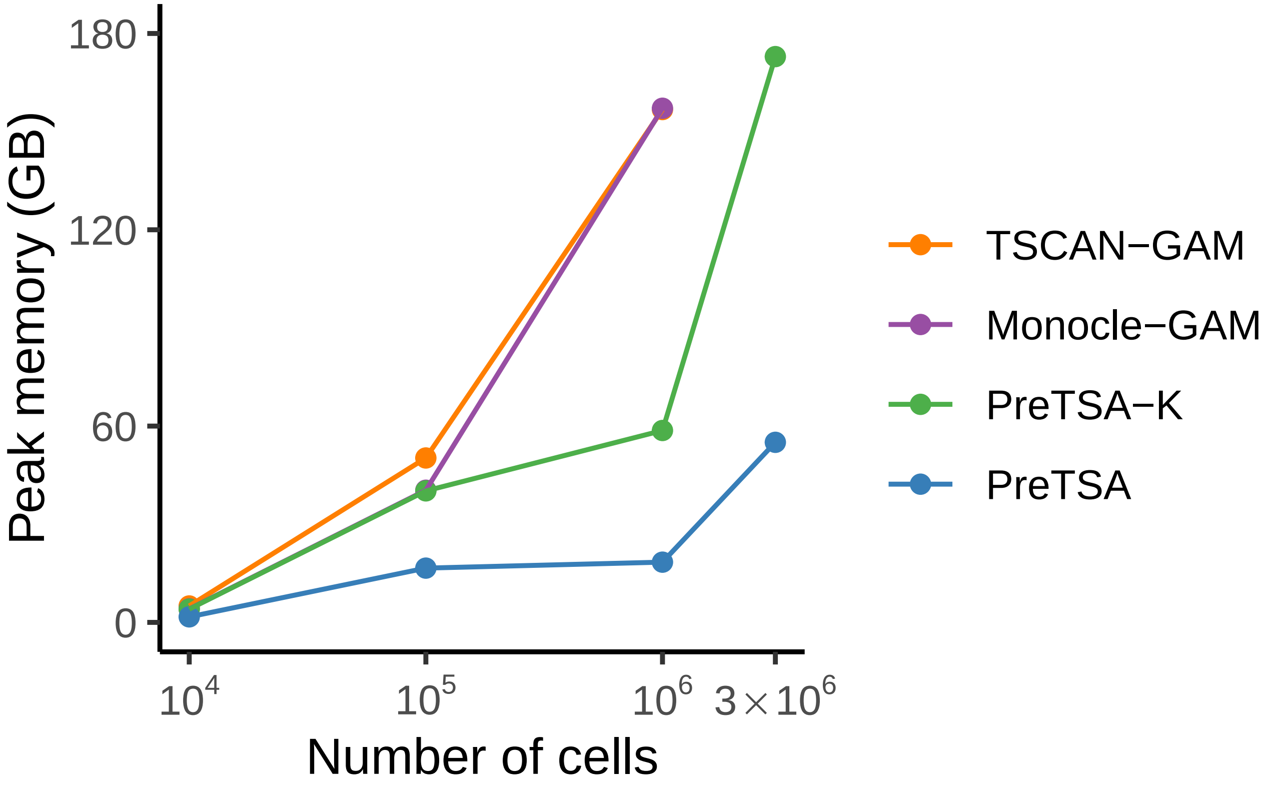

Fig. S5. Peak memory usage of different methods for fitting temporal patterns in simulated data generated by sampling from the human PBMC scRNA-seq dataset with varying numbers of cells. For scenarios in which a method exceeded the one-week time limit, its memory usage could not be obtained and is therefore not shown in the plot.

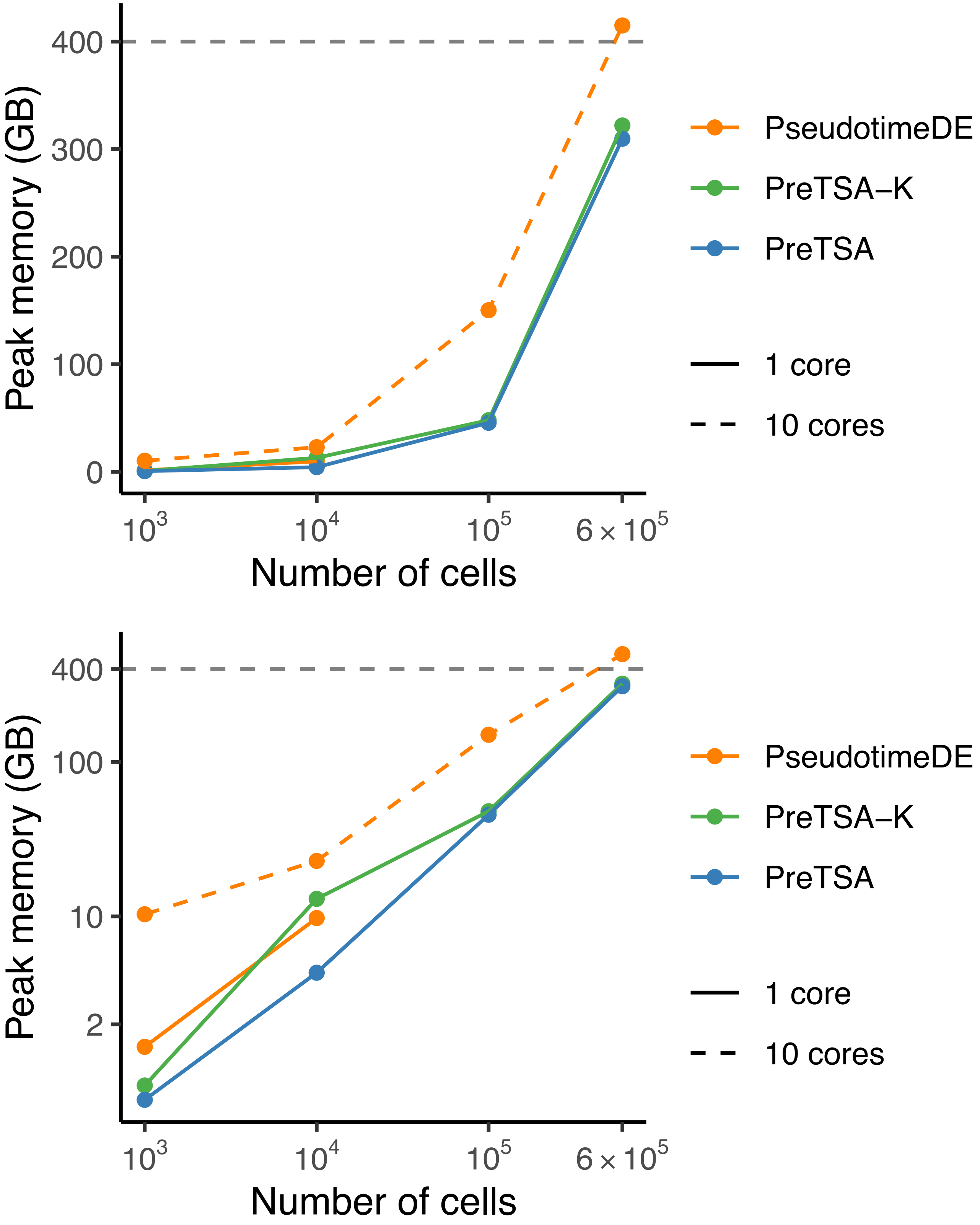

Fig. S6. Peak memory usage of different methods for testing TVGs in simulated data generated by sampling from the human PBMC scRNA-seq dataset with varying numbers of cells. For scenarios in which a method exceeded the one-week time limit, its memory usage could not be obtained and is therefore not shown in the plot.

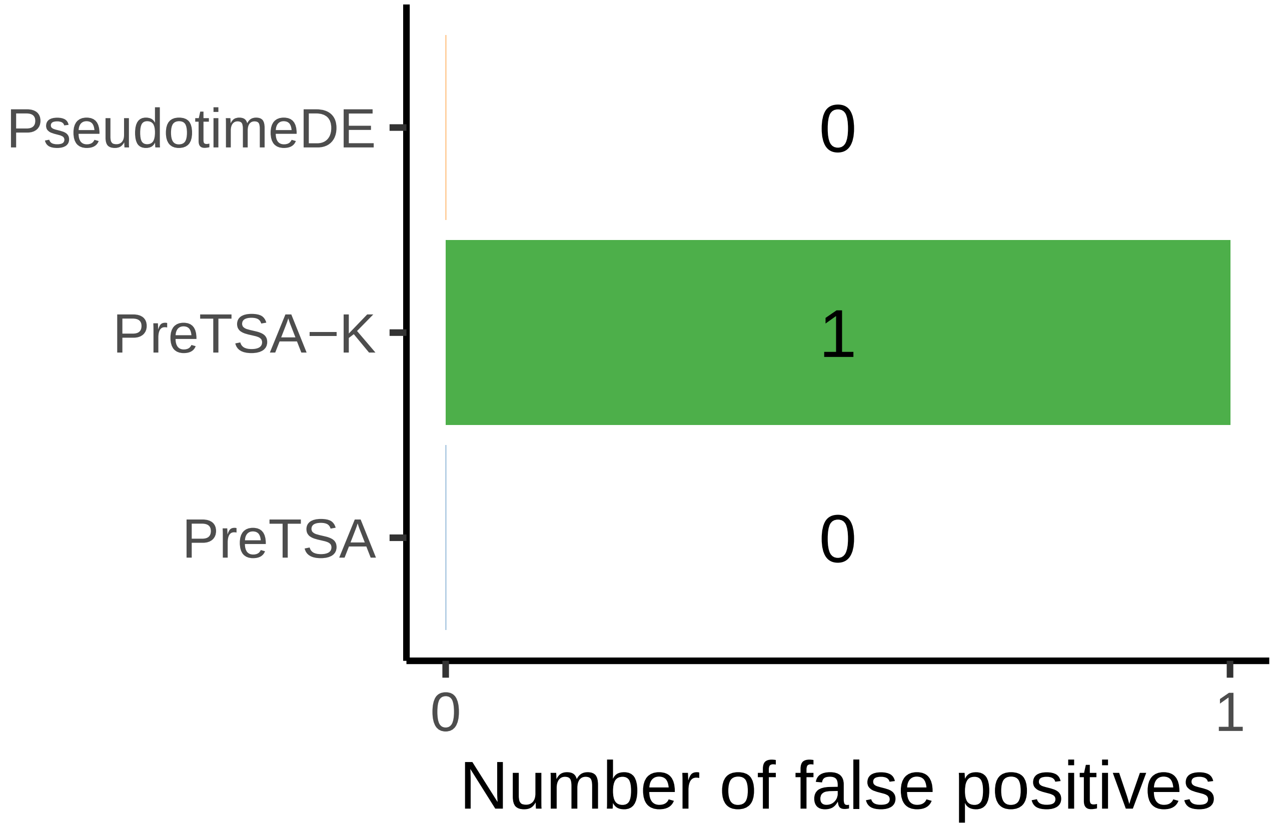

Fig. S7. Number of false positives detected by different methods in a null dataset derived from the human PBMC scRNA-seq dataset.

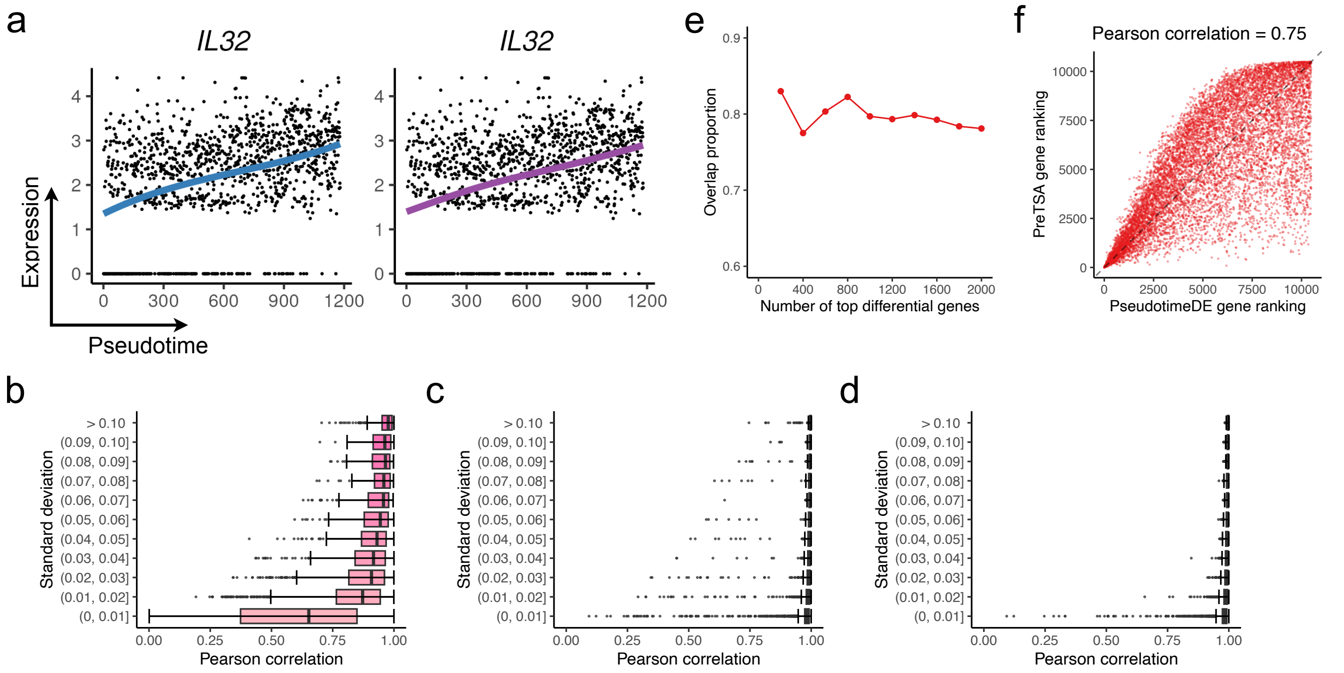

Fig. S8. Additional results of the original human PBMC scRNA-seq dataset. **a**, Scatterplots showing the expression of *IL32* (y-axis) and the pseudotime (x-axis). The curve indicates the fitted curve by `PreTSA` (left) or by Monocle-GAM (right). **b**, Pearson correlations between fitted values by TSCAN-GAM and by `PreTSA` (x-axis), grouped by the standard deviation of fitted values by TSCAN-GAM (y-axis). **c**, Pearson correlations between fitted values by Monocle-GAM and by `PreTSA-K` (x-axis), grouped by the standard deviation of fitted values by Monocle-GAM (y-axis). **d**, Pearson correlations between fitted values by Monocle-GAM and by `PreTSA` (x-axis), grouped by the standard deviation of fitted values by Monocle-GAM (y-axis). **e**, Overlap proportion for different numbers of top differential genes using `PreTSA` and `PseudotimeDE`. **f**, Gene rankings by `PreTSA` (y-axis) and by `PseudotimeDE` (x-axis).

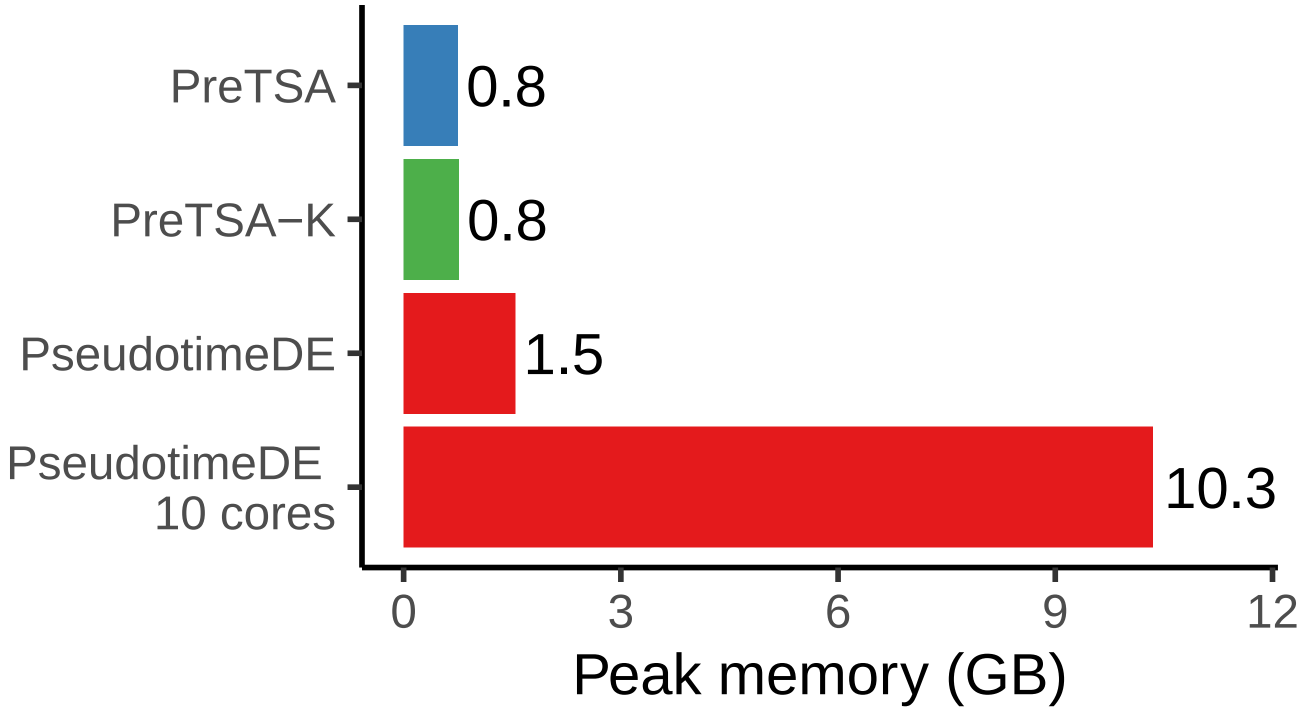

Fig. S9. Peak memory usage of different methods for testing TVGs in the human PBMC scRNA-seq dataset.

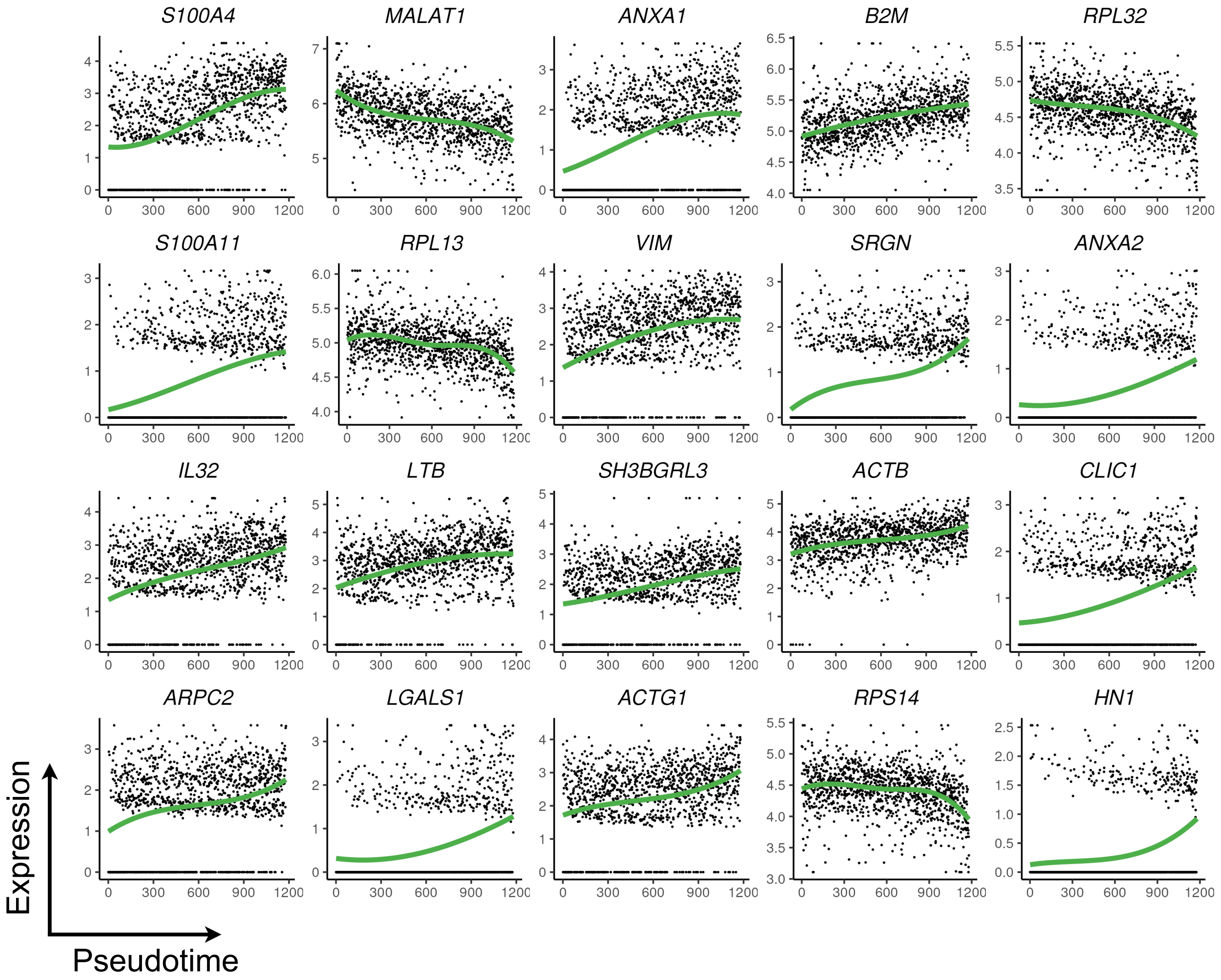

Fig. S10. Expression of the top 20 TVGs identified by `PreTSA-K` (y-axis) along pseudotime (x-axis) in the human PBMC scRNA-seq dataset. Dots represent single-cell expression values, and curves show the fitted trajectories by `PreTSA-K`.

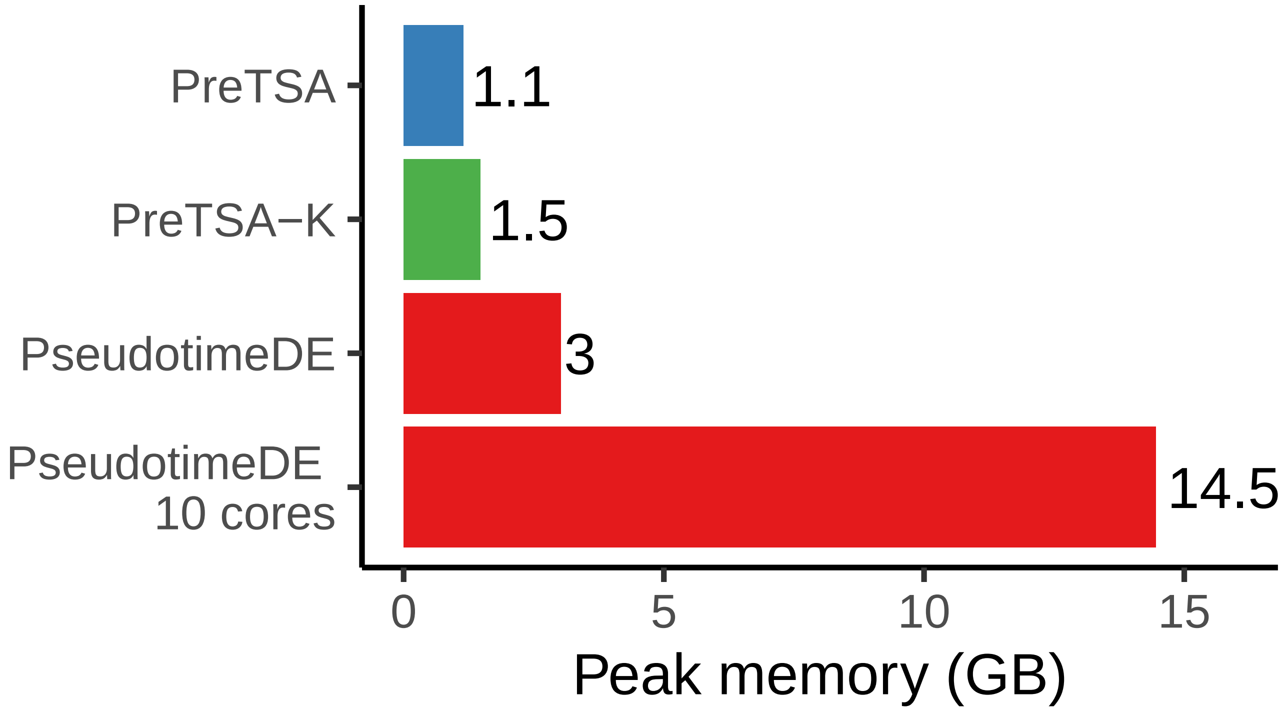

Fig. S11. Peak memory usage of different methods for testing TVGs in the beta branch of the mouse pancreas scRNA-seq dataset.

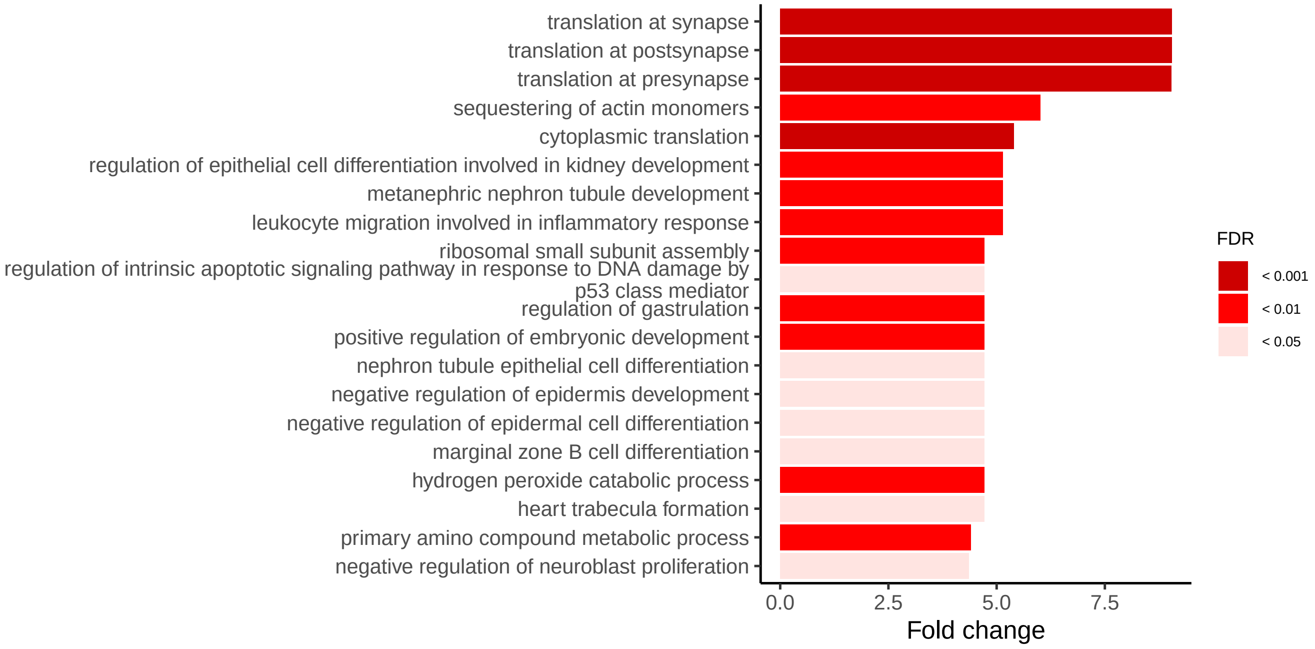

Fig. S12. Top GO terms enriched among TVGs identified by `PreTSA-K` in the alpha branch of the mouse pancreas scRNA-seq dataset.

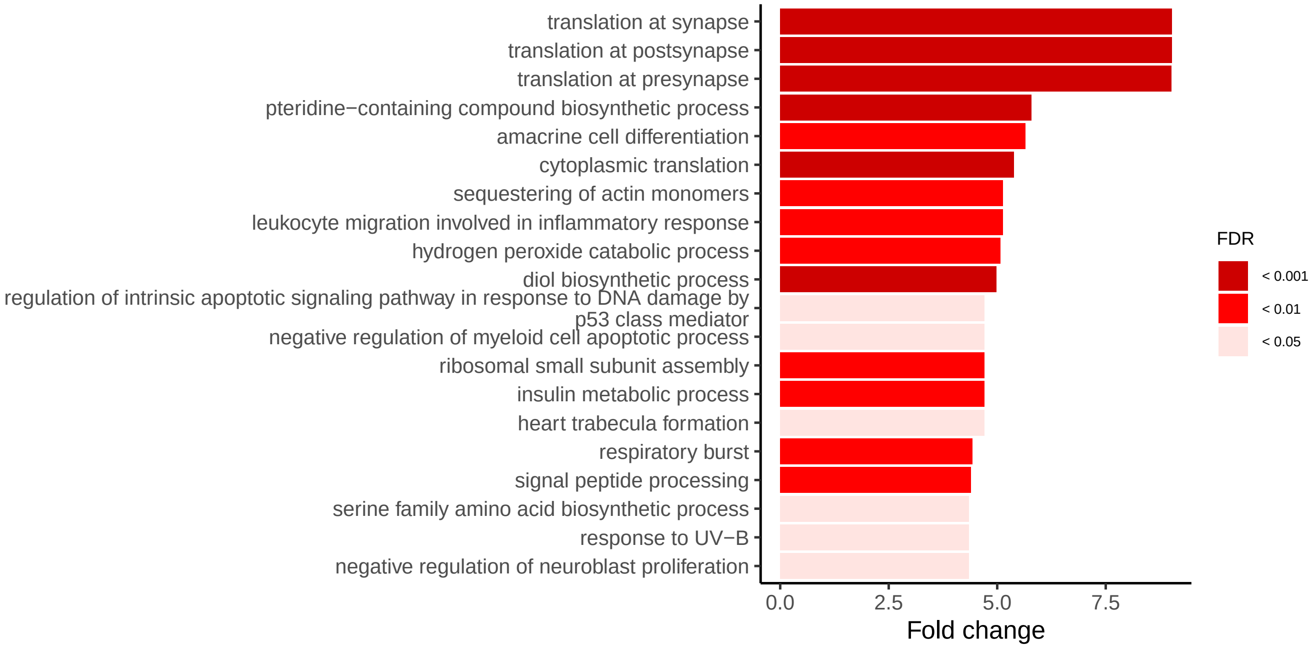

Fig. S13. Top GO terms enriched among TVGs identified by `PreTSA-K` in the beta branch of the mouse pancreas scRNA-seq dataset.

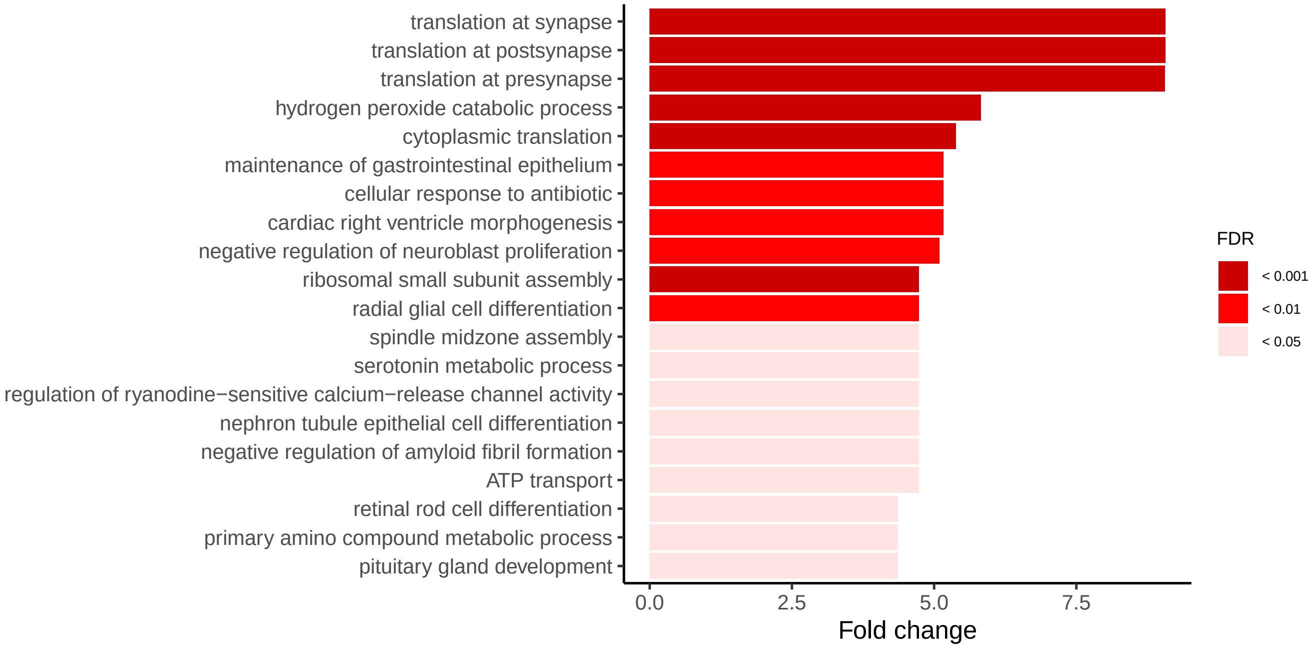

Fig. S14. Top GO terms enriched among TVGs identified by `PreTSA-K` in the delta branch of the mouse pancreas scRNA-seq dataset.

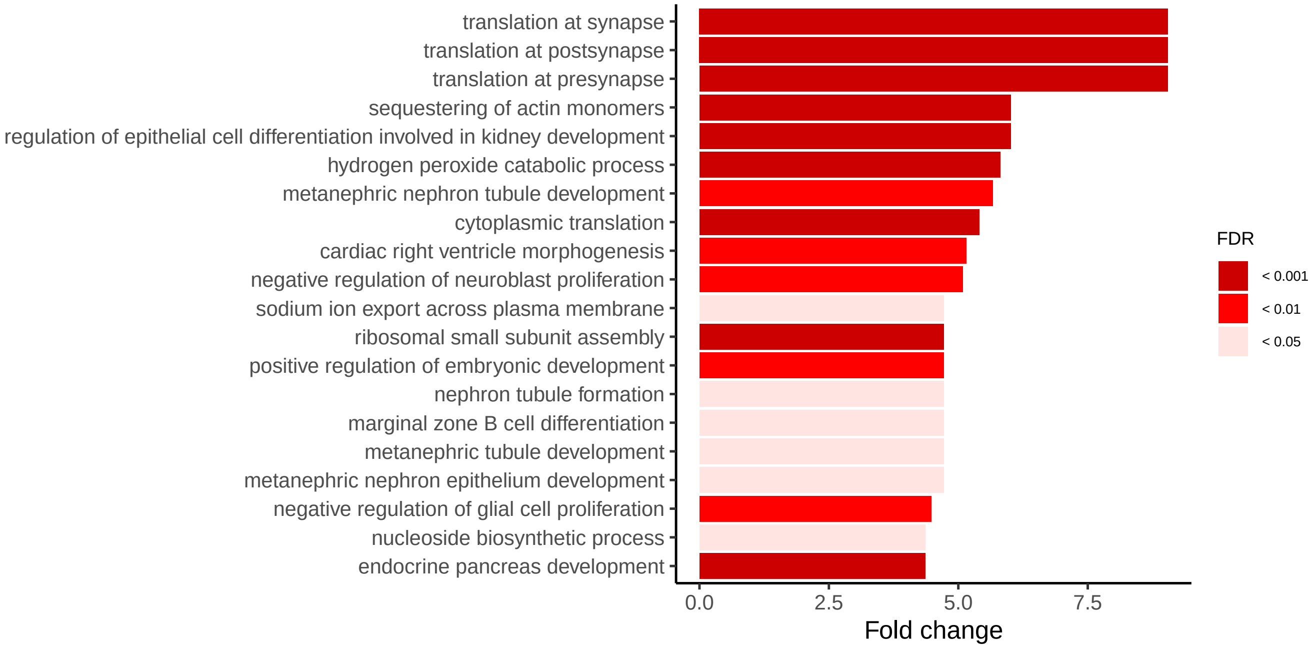

Fig. S15. Top GO terms enriched among TVGs identified by `PreTSA-K` in the epsilon branch of the mouse pancreas scRNA-seq dataset.

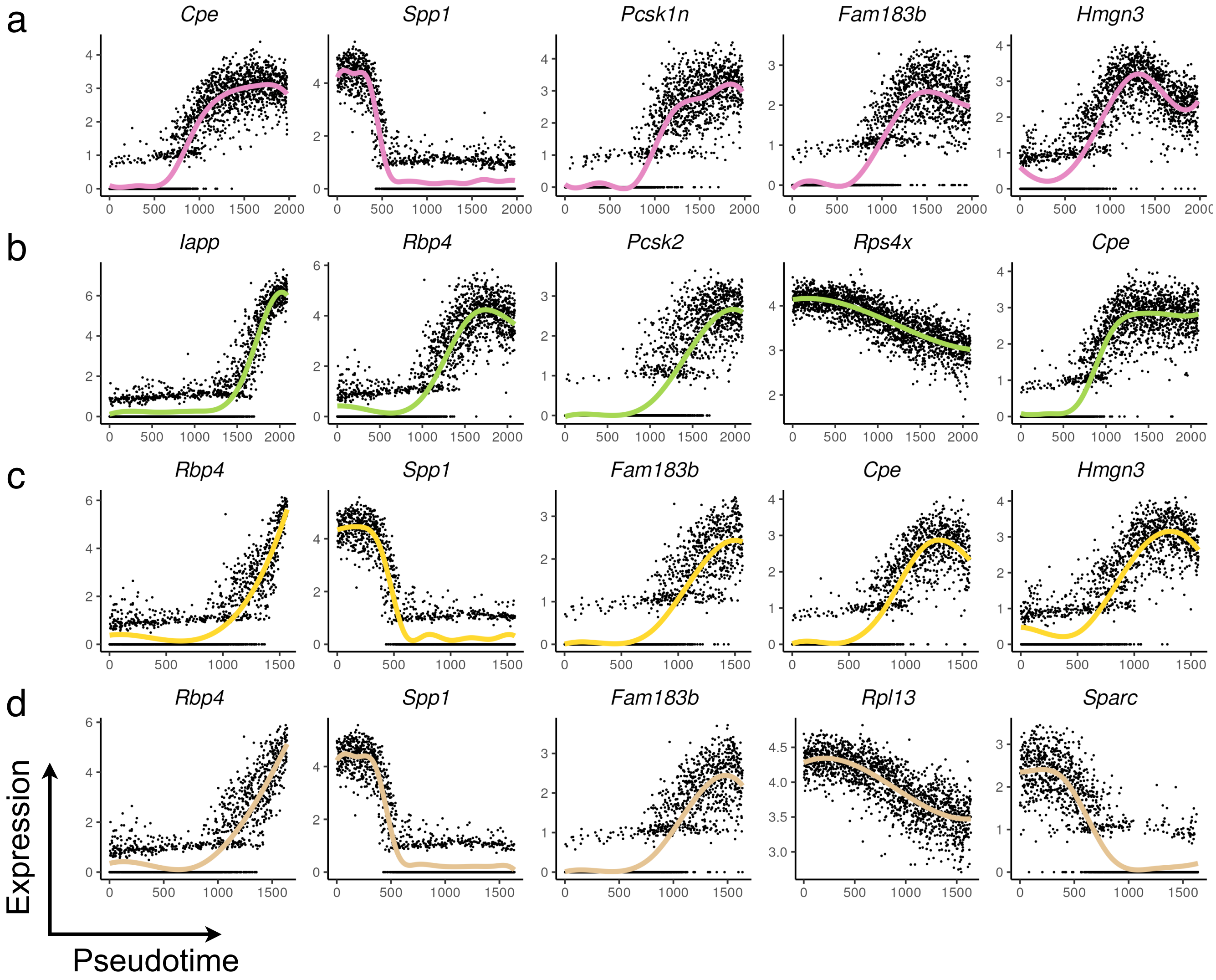

Fig. S16. Expression of the top 5 TVGs identified by `PreTSA-K` (y-axis) along pseudotime (x-axis) in the alpha branch (**a**), beta branch (**b**), delta branch (**c**), and epsilon branch (**d**) of the mouse pancreas scRNA-seq dataset. Dots represent single-cell expression values, and curves show the fitted trajectories by `PreTSA-K`.

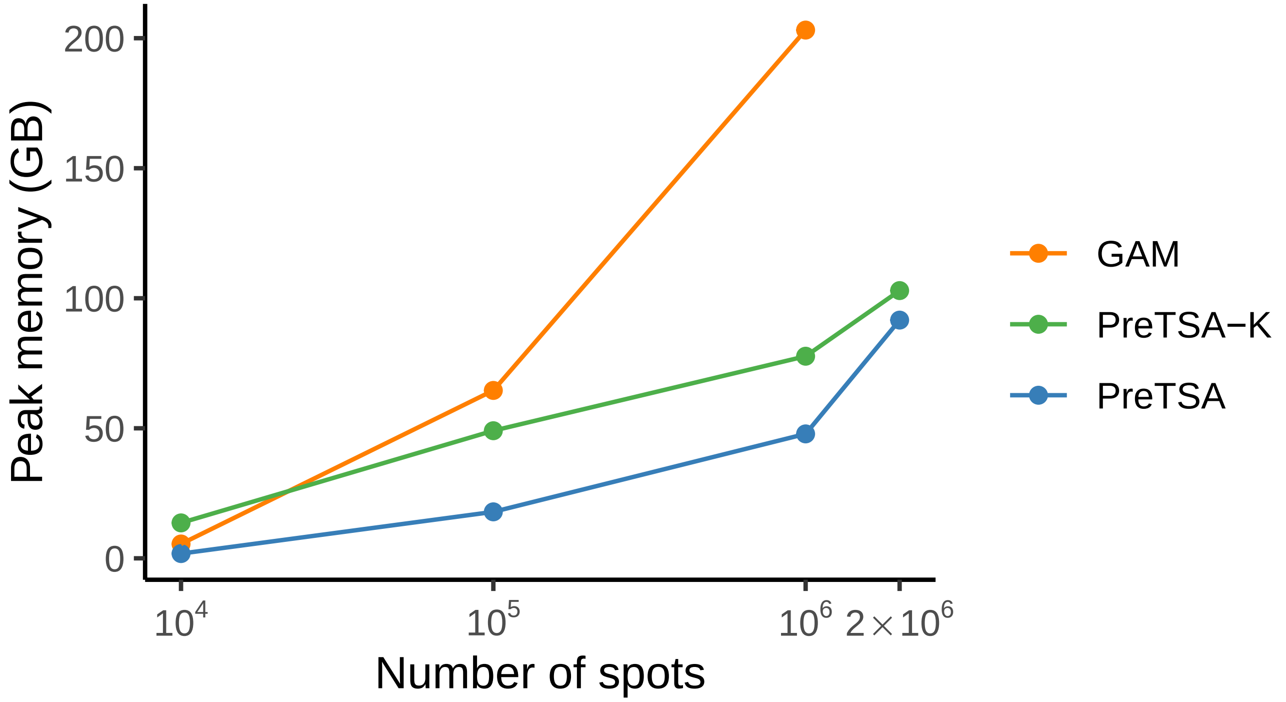

Fig. S17. Peak memory usage of different methods for fitting spatial patterns in simulated data generated by sampling from the Visium human heart dataset with varying numbers of spots. For scenarios in which a method exceeded the one-week time limit, its memory usage could not be obtained and is therefore not shown in the plot.

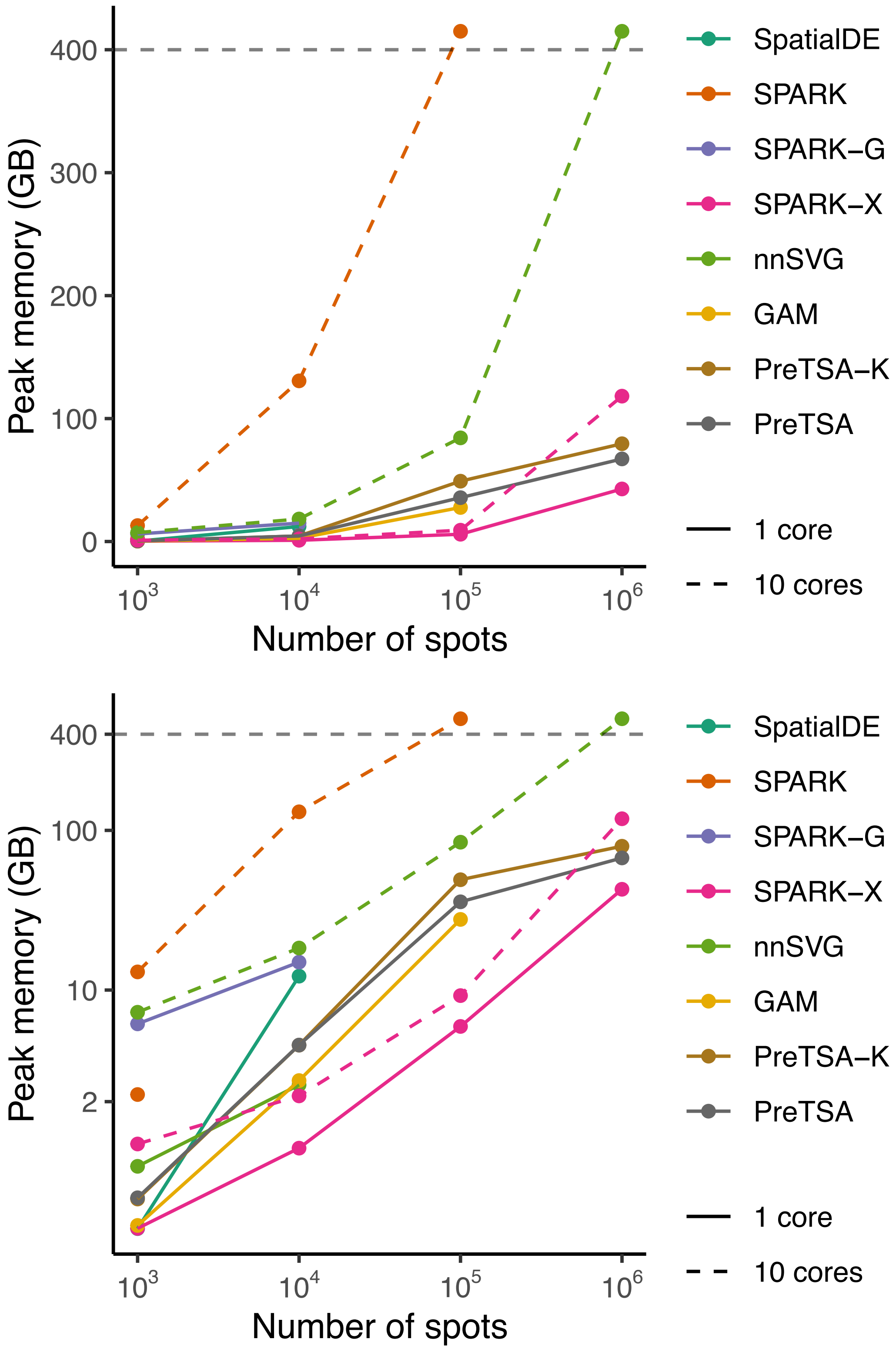

Fig. S18. Peak memory usage of different methods for testing SVGs in simulated data generated by sampling from the Visium human heart dataset with varying numbers of spots. For scenarios in which a method exceeded the one-week time limit, its memory usage could not be obtained and is therefore not shown in the plot. The bottom panel shows the log-scale version of the top panel.

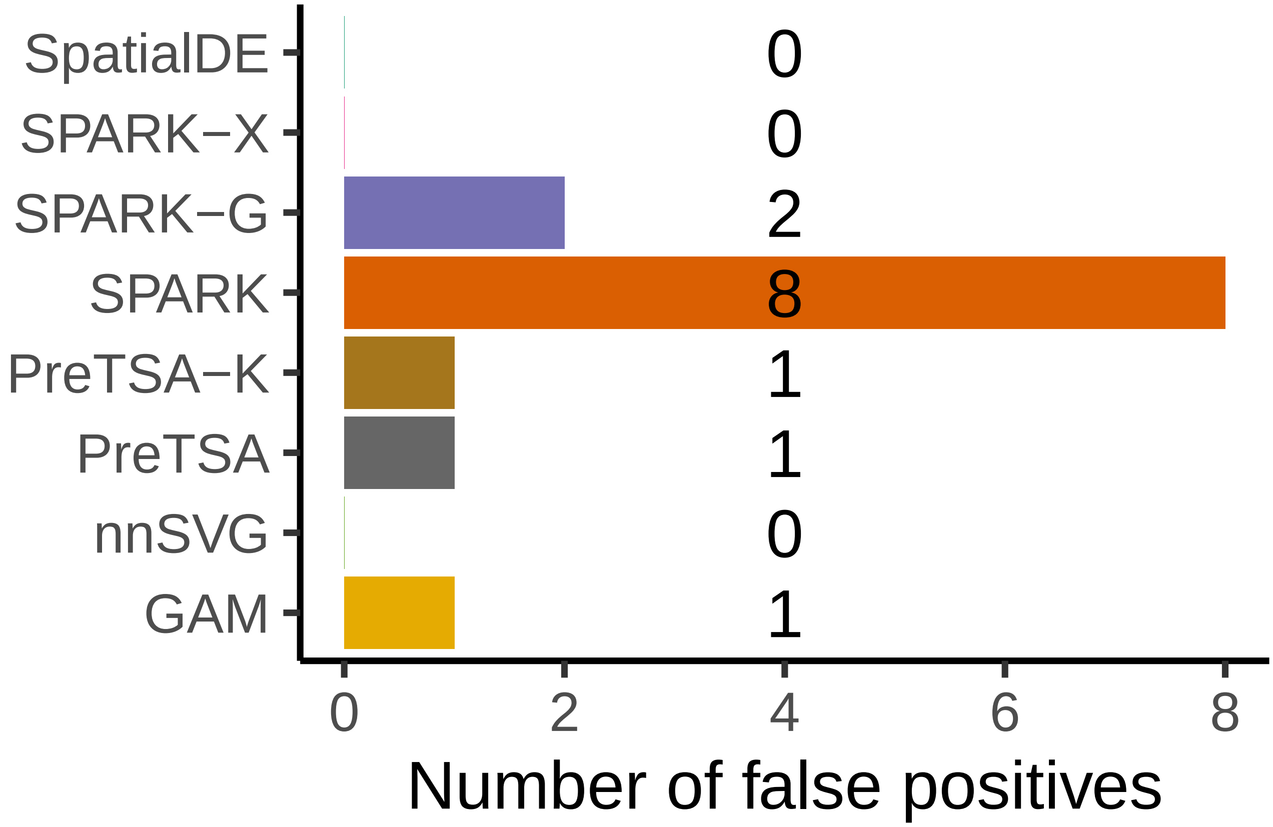

Fig. S19. Number of false positives detected by different methods in a null dataset derived from the Visium human heart dataset.

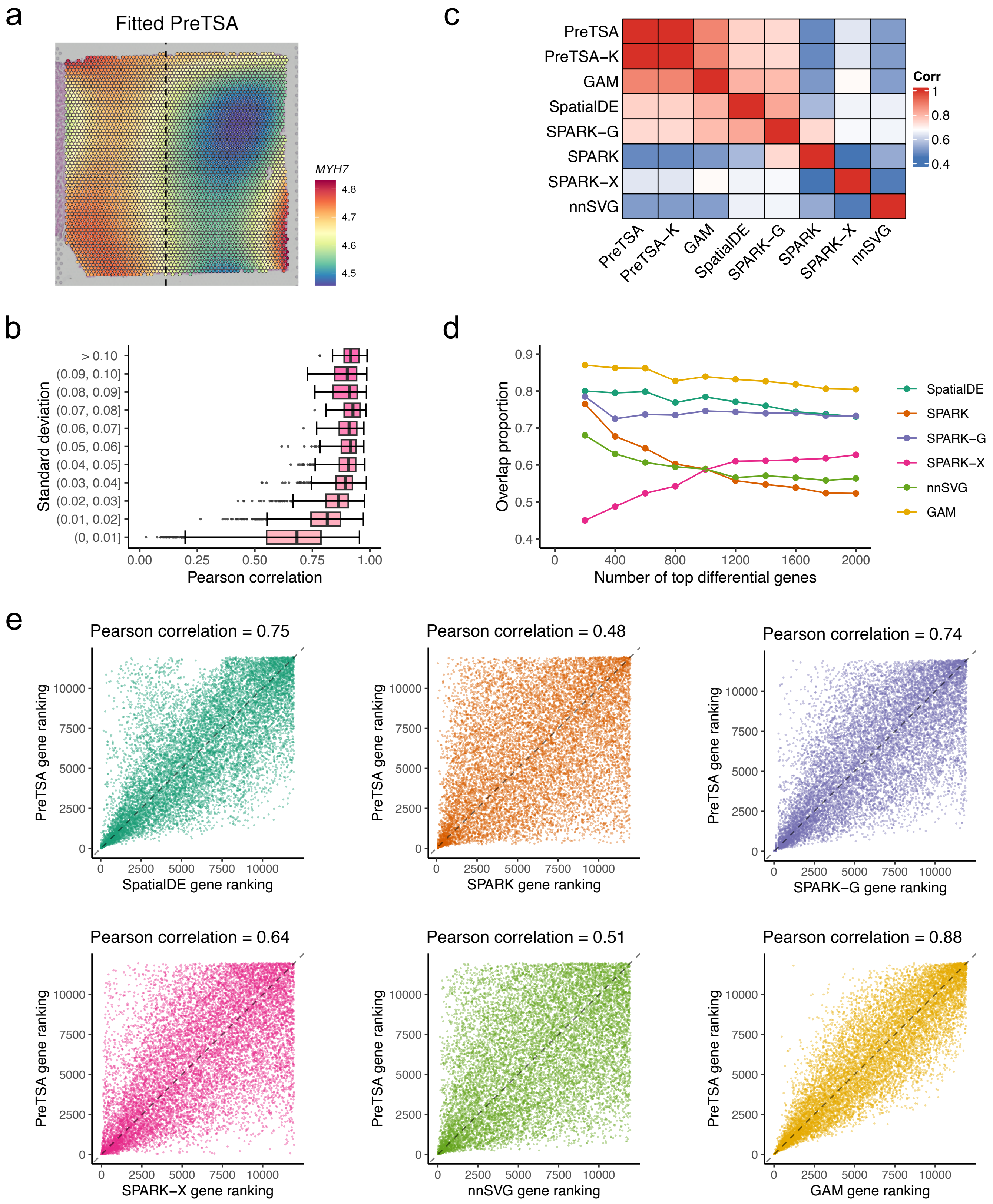

Fig. S20. Additional results of the original Visium human heart dataset. **a**, Spatial expression pattern of *MYH7* fitted by `PreTSA`. **b**, Pearson correlations between fitted values by GAM and by `PreTSA` (x-axis), grouped by the standard deviation of fitted values by GAM (y-axis). **c**, Pearson correlations for the overall gene rankings between different methods. **d**, Overlap proportion for different numbers of top differential genes using `PreTSA` and other methods. **e**, Gene rankings by `PreTSA` (y-axis) and by other methods (x-axis).

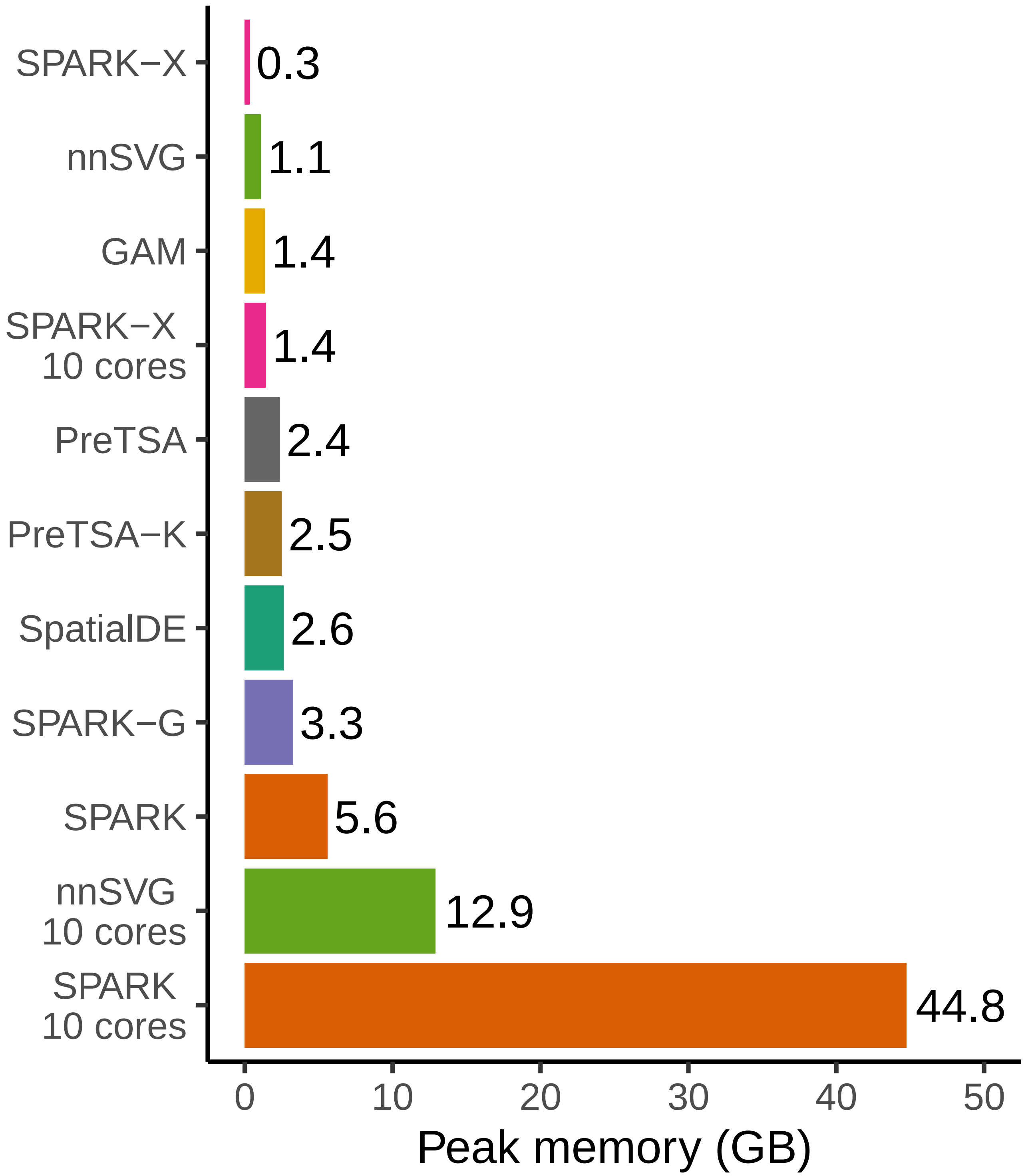

Fig. S21. Peak memory usage of different methods for testing SVGs in the Visium human heart dataset.

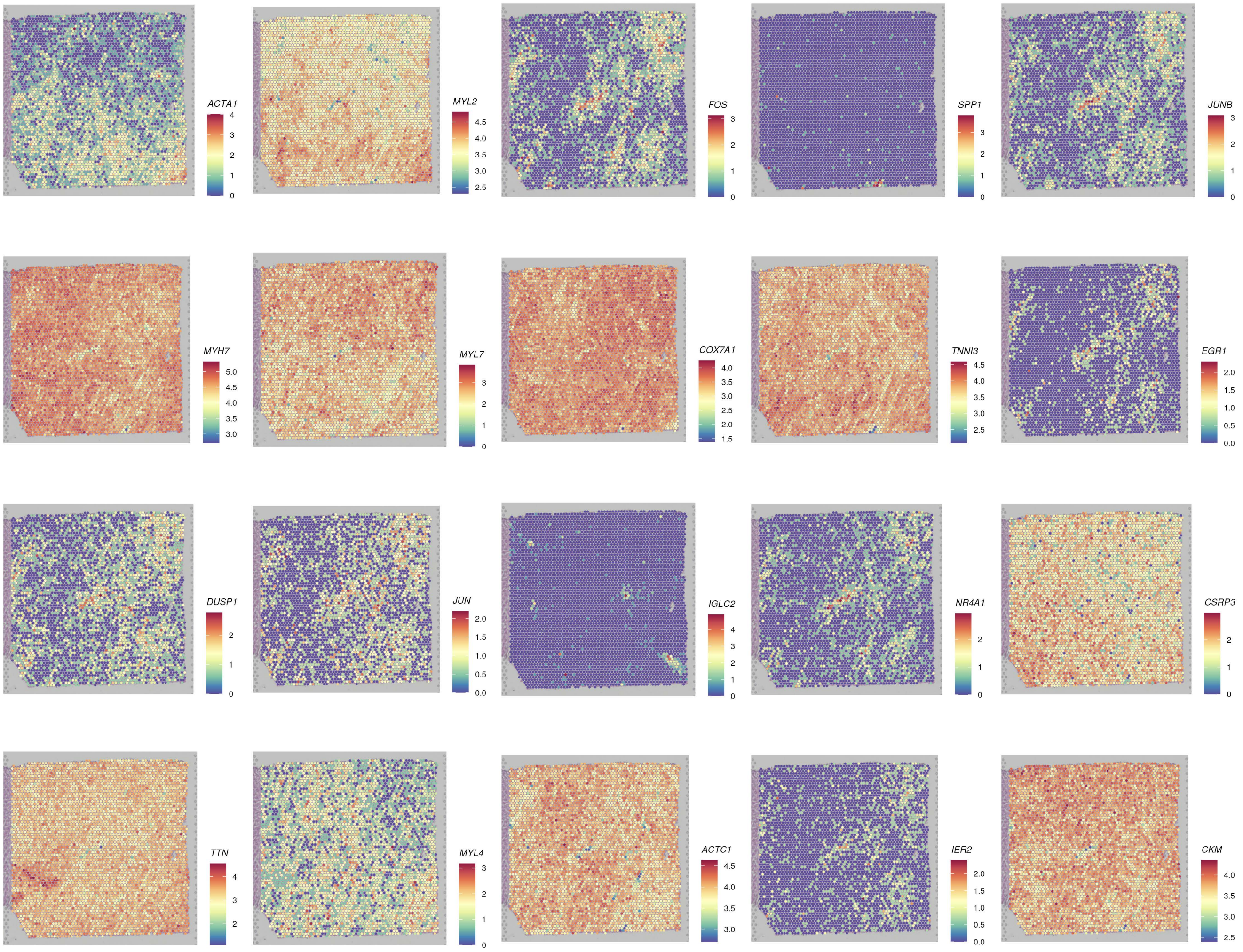

Fig. S22. Original expression of the top 20 SVGs identified by `PreTSA-K` in the Visium human heart dataset.

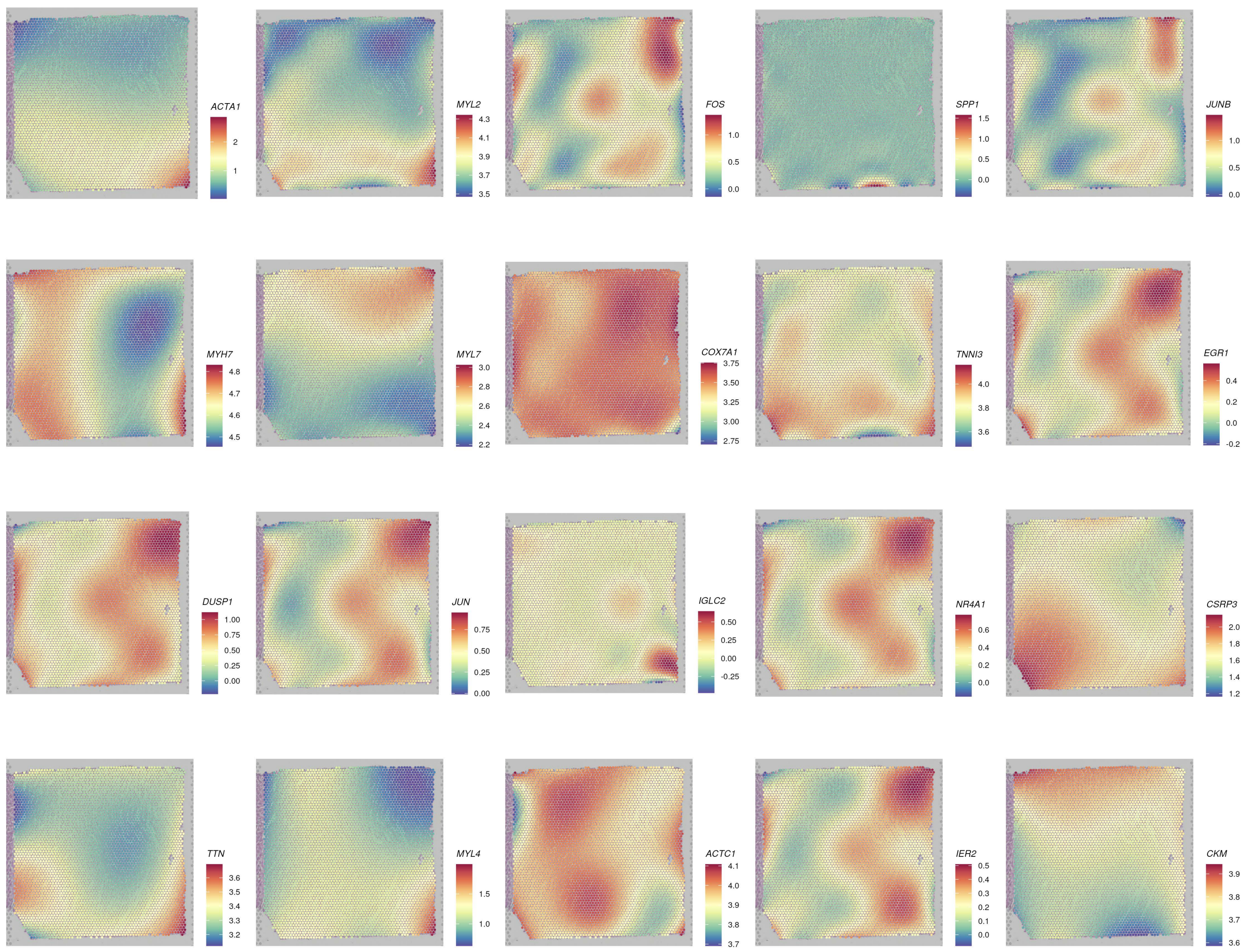

Fig. S23. Spatial expression patterns fitted by `PreTSA-K` for the top 20 SVGs identified by `PreTSA-K` in the Visium human heart dataset.

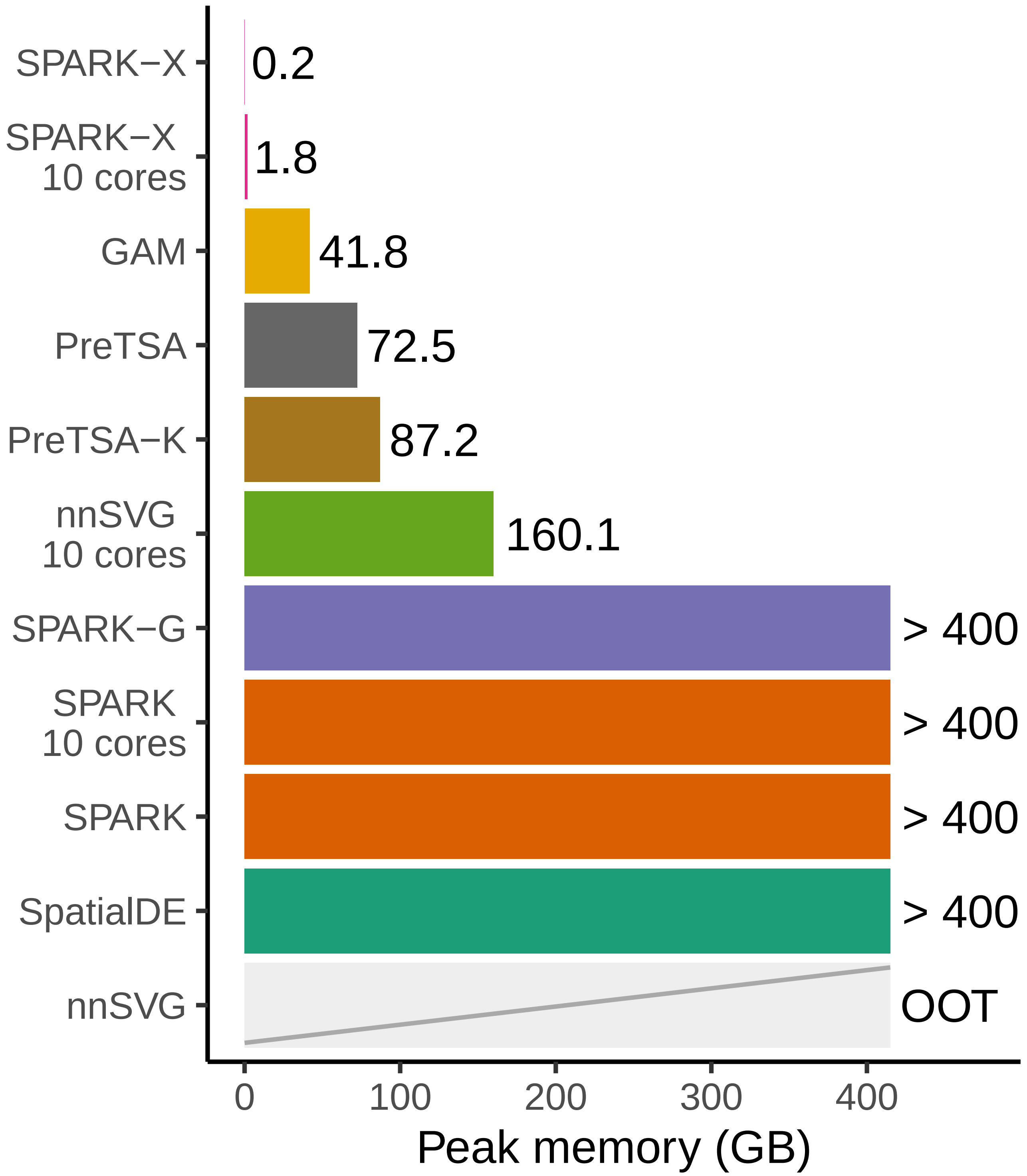

Fig. S24. Peak memory usage of different methods for testing SVGs in the HDST mouse olfactory bulb dataset.

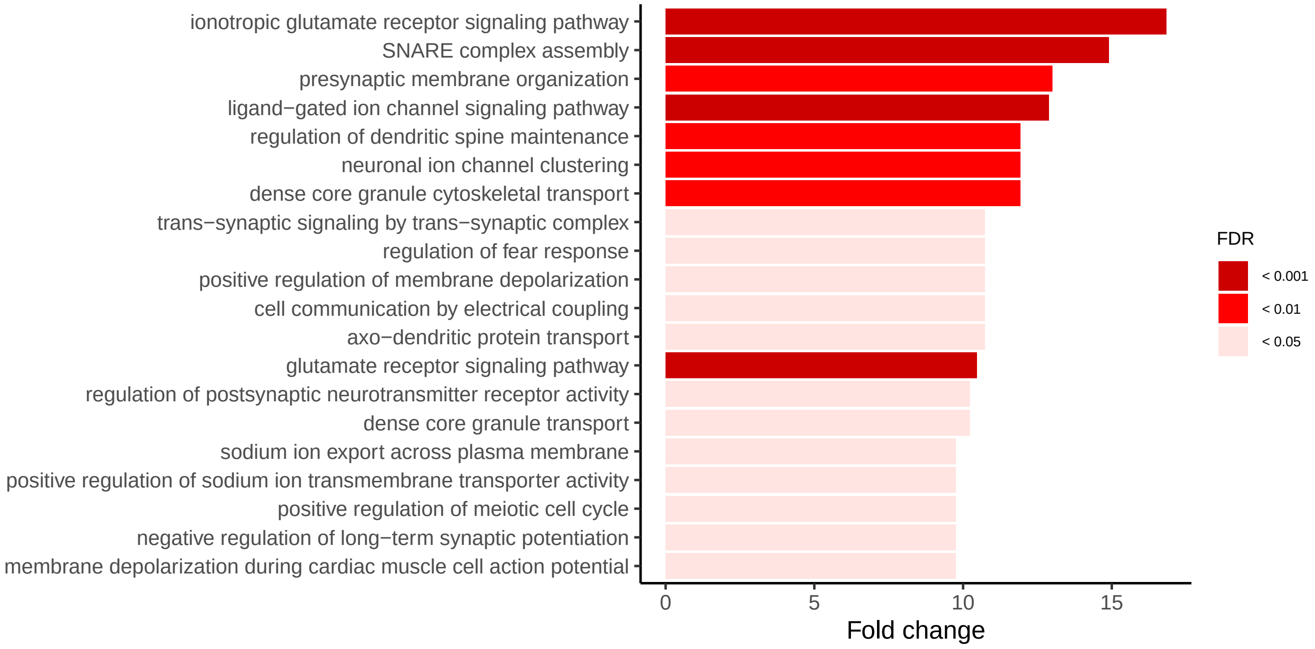

Fig. S25. Top GO terms enriched among SVGs identified by `PreTSA-K` in the HDST mouse olfactory bulb dataset.

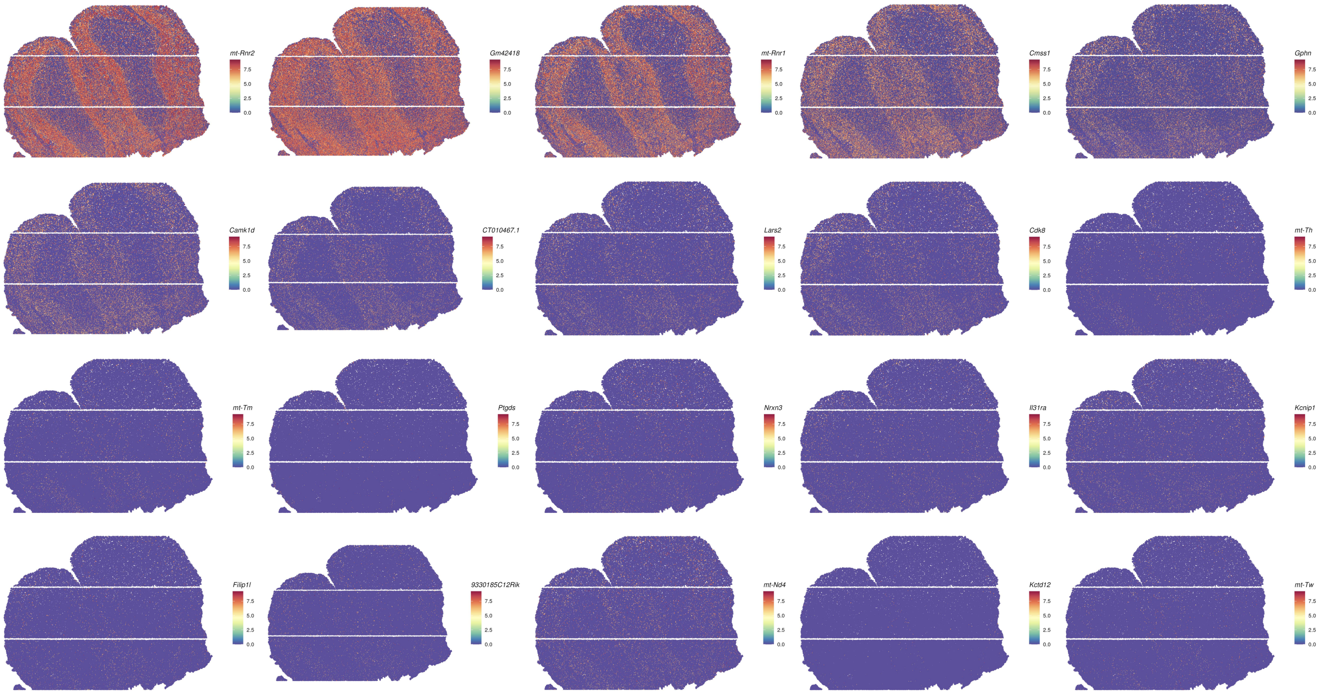

Fig. S26. Original expression of the top 20 SVGs identified by `PreTSA-K` in the HDST mouse olfactory bulb dataset.

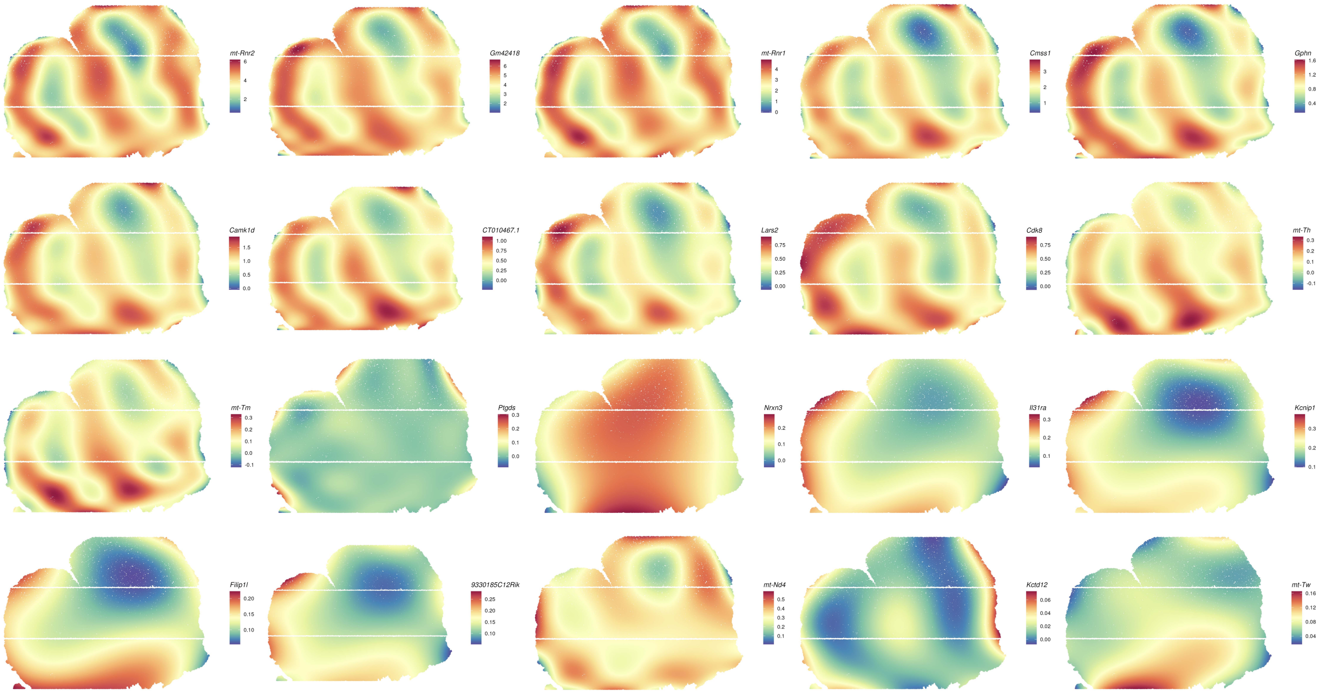

Fig. S27. Spatial expression patterns fitted by `PreTSA-K` for the top 20 SVGs identified by `PreTSA-K` in the HDST mouse olfactory bulb dataset.

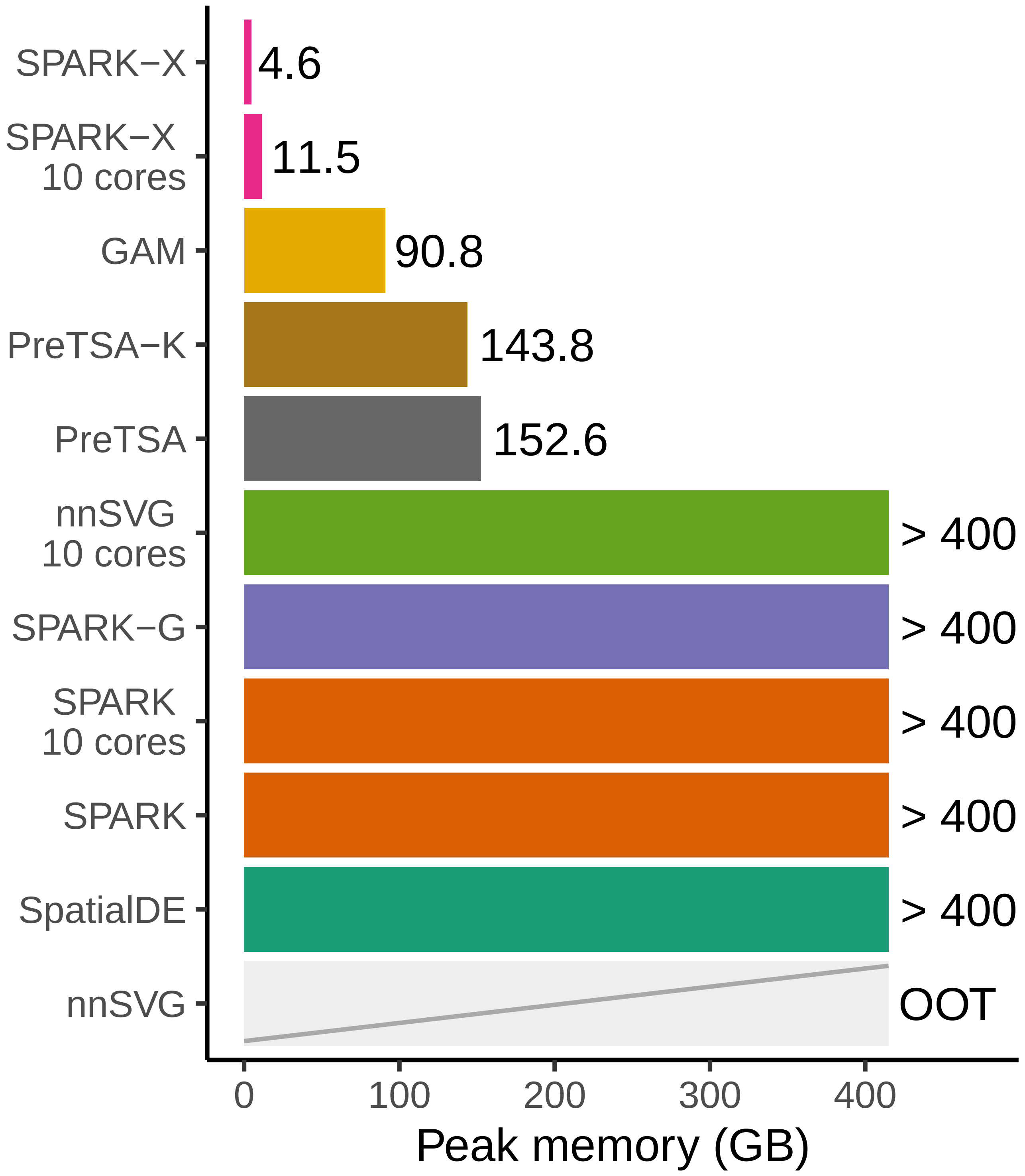

Fig. S28. Peak memory usage of different methods for testing SVGs in the Visium HD human colon cancer dataset.

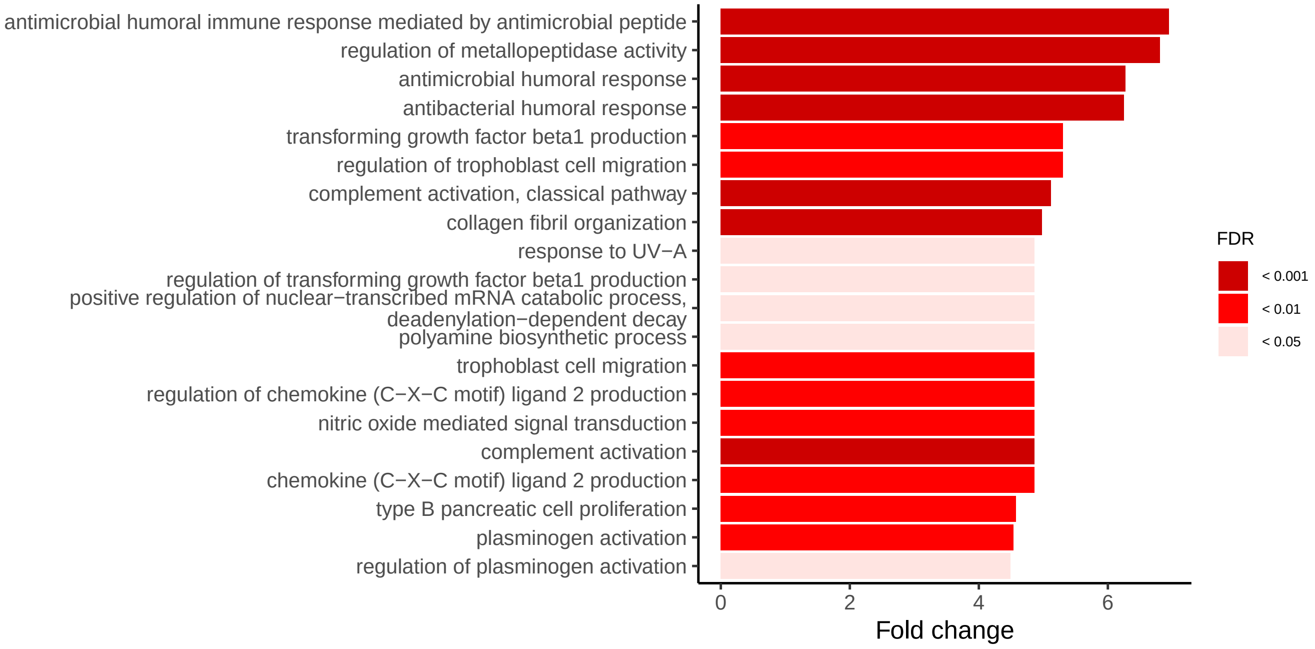

Fig. S29. Top GO terms enriched among SVGs identified by `PreTSA-K` in the Visium HD human colon cancer dataset.

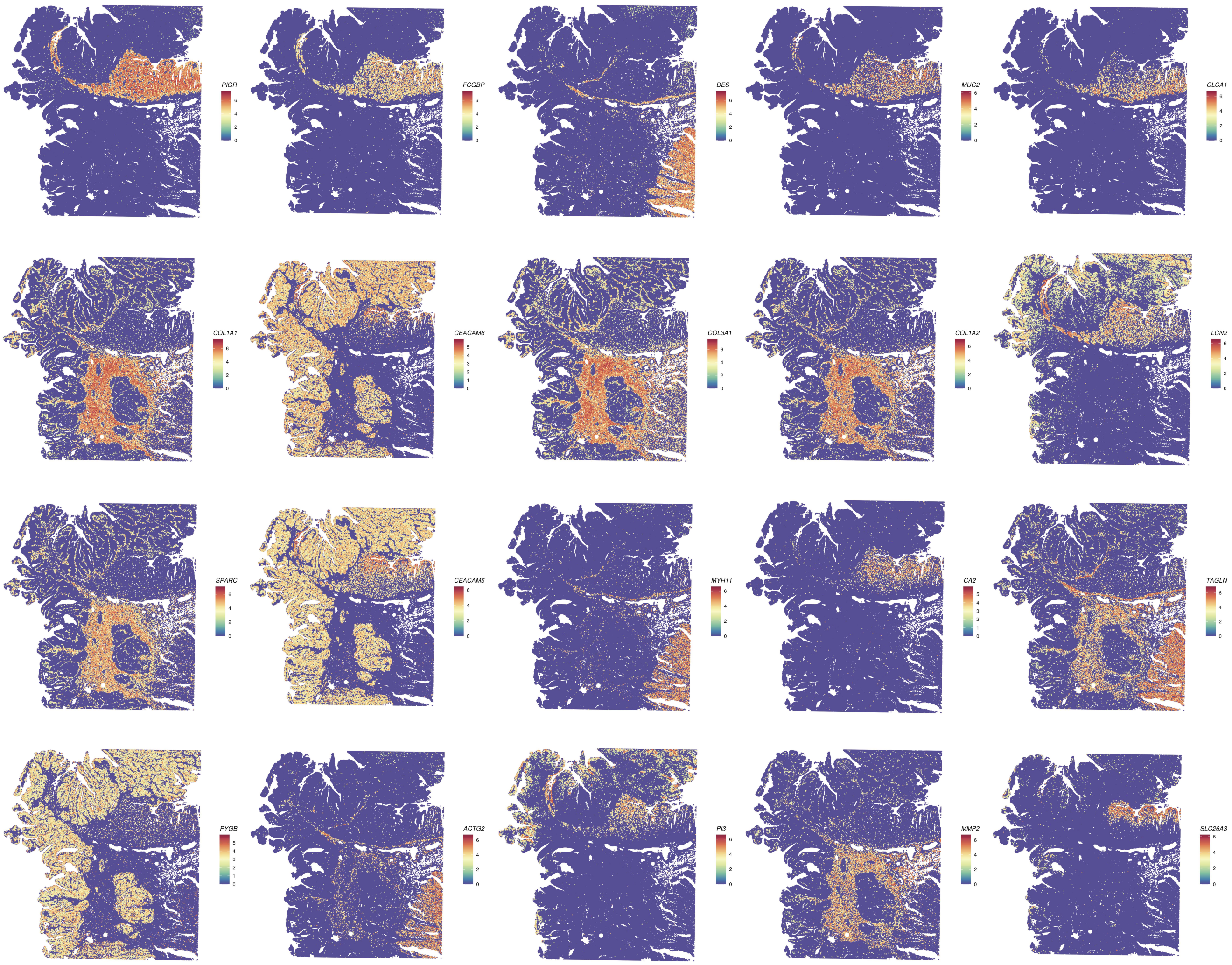

Fig. S30. Original expression of the top 20 SVGs identified by `PreTSA-K` in the Visium HD human colon cancer dataset.

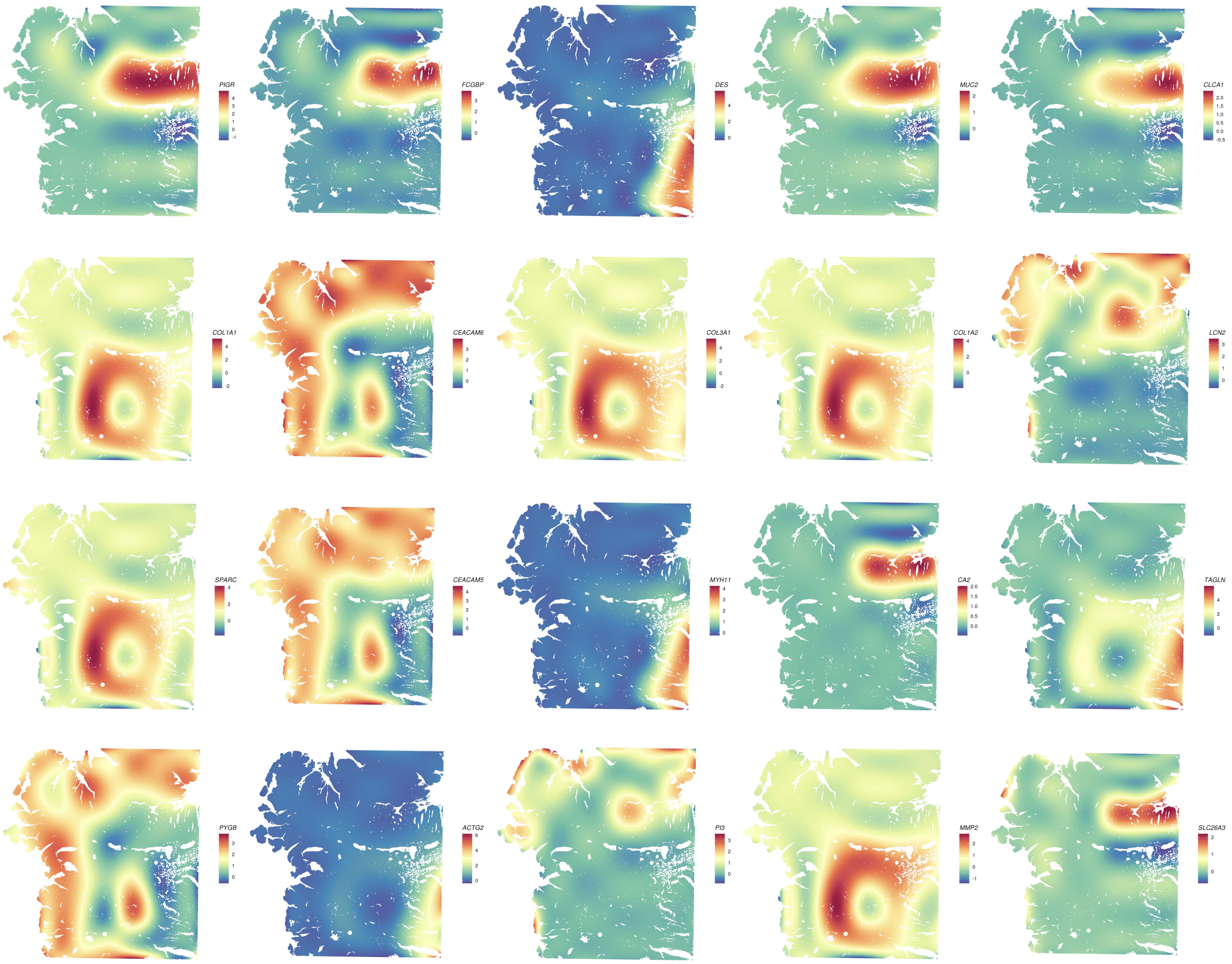

Fig. S31. Spatial expression patterns fitted by `PreTSA-K` for the top 20 SVGs identified by `PreTSA-K` in the Visium HD human colon cancer dataset.

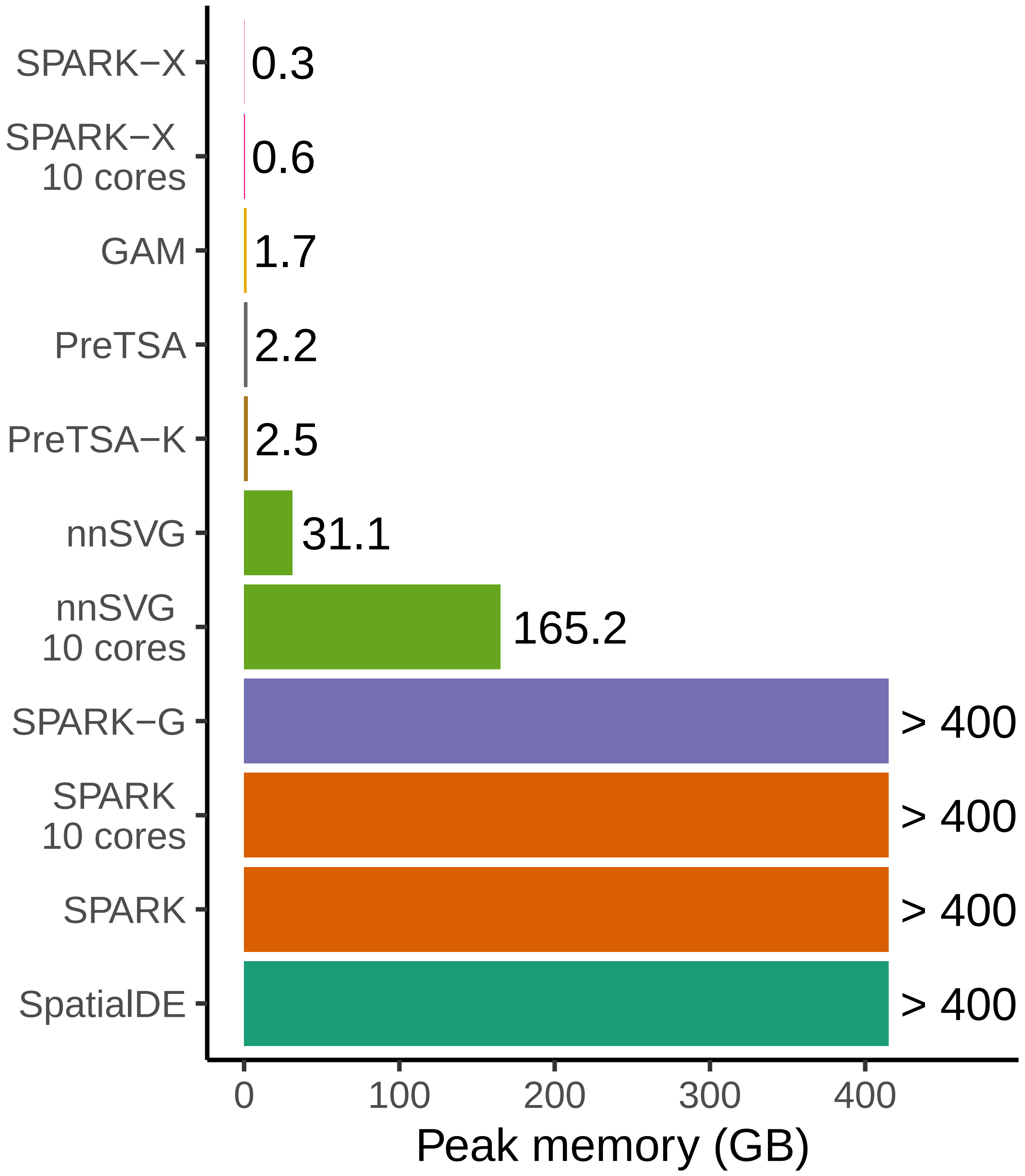

Fig. S32. Peak memory usage of different methods for testing SVGs in the Xenium human lung cancer dataset.

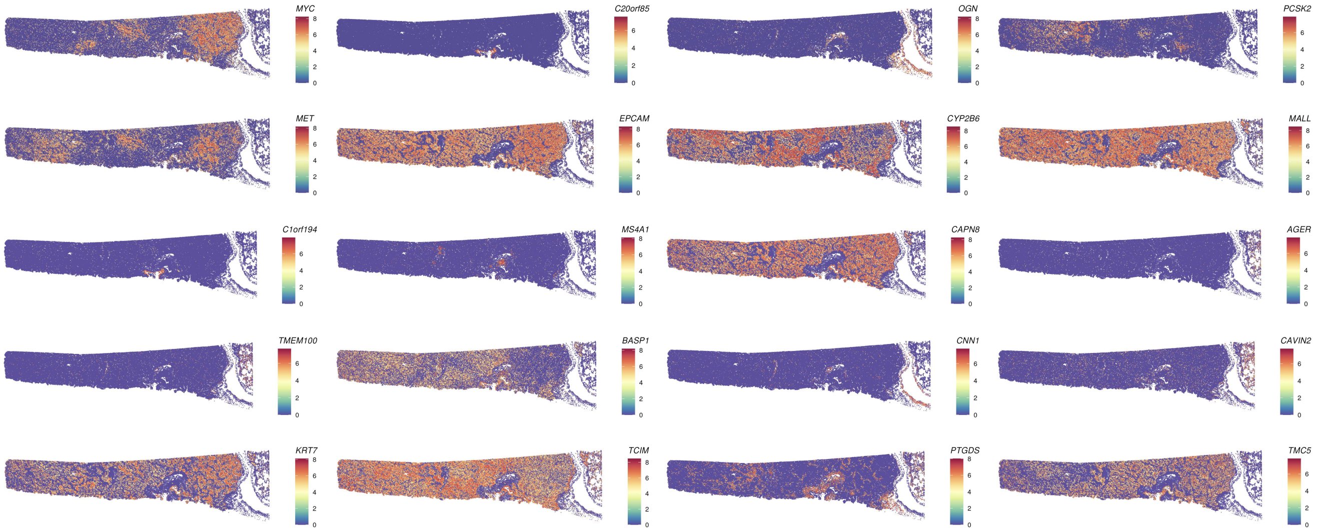

Fig. S33. Original expression of the top 20 SVGs identified by `PreTSA-K` in the Xenium human lung cancer dataset.

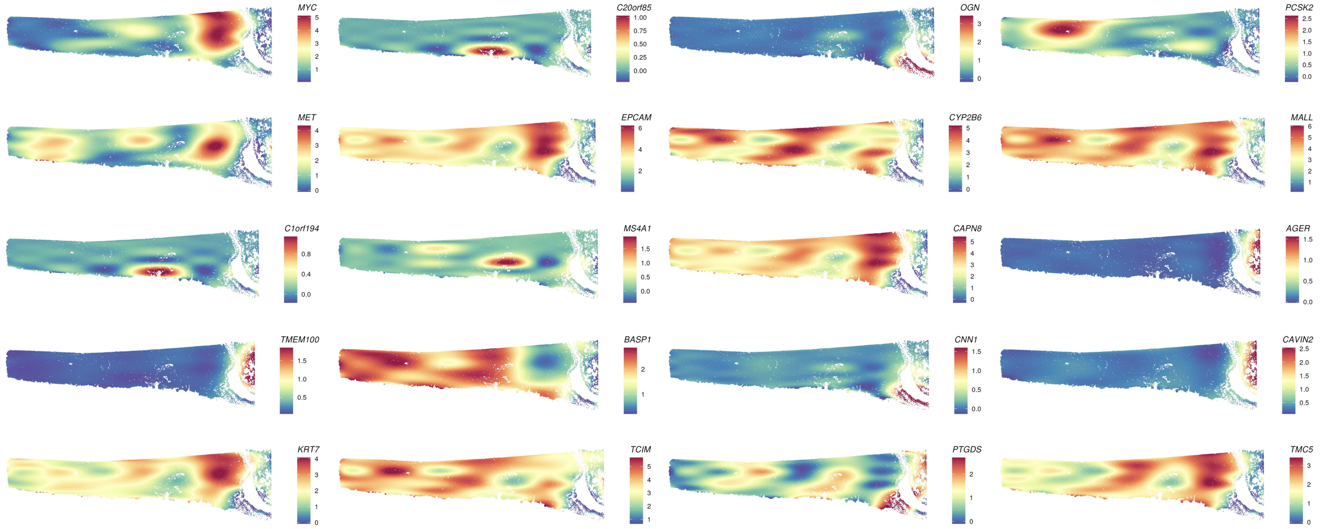

Fig. S34. Spatial expression patterns fitted by `PreTSA-K` for the top 20 SVGs identified by `PreTSA-K` in the Xenium human lung cancer dataset.

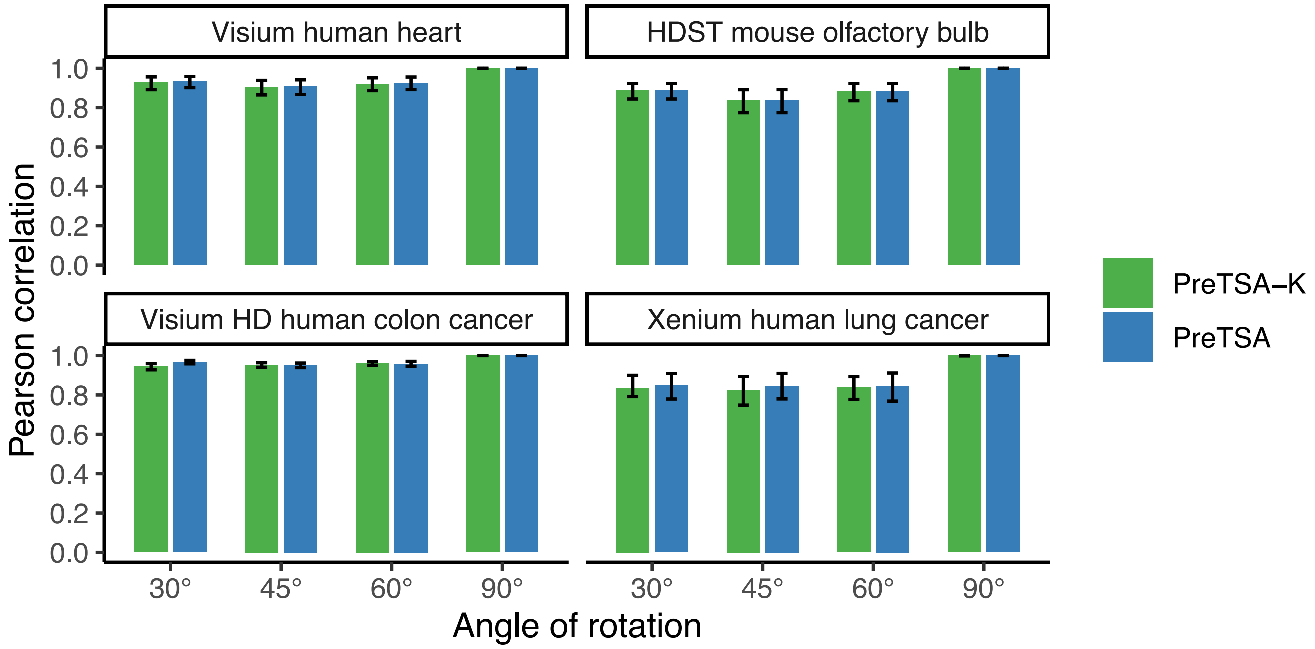

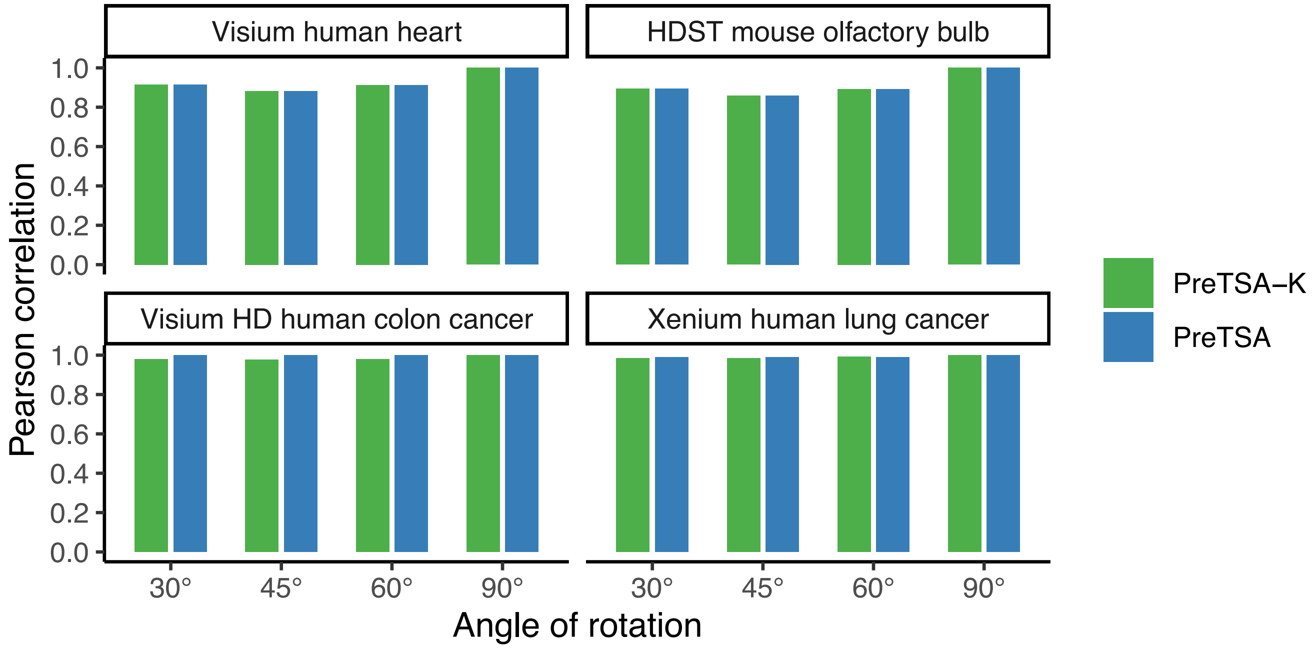

Fig. S35. Pearson correlations between fitted values (top) or overall gene rankings (bottom) obtained by `PreTSA` or `PreTSA-K` from the original datasets and from datasets rotated at different angles. Median correlations of fitted values were calculated using genes whose fitted values in the original dataset had standard deviations greater than 0.05. Error bars represent the interquartile range (IQR), spanning the 25th to 75th percentiles.

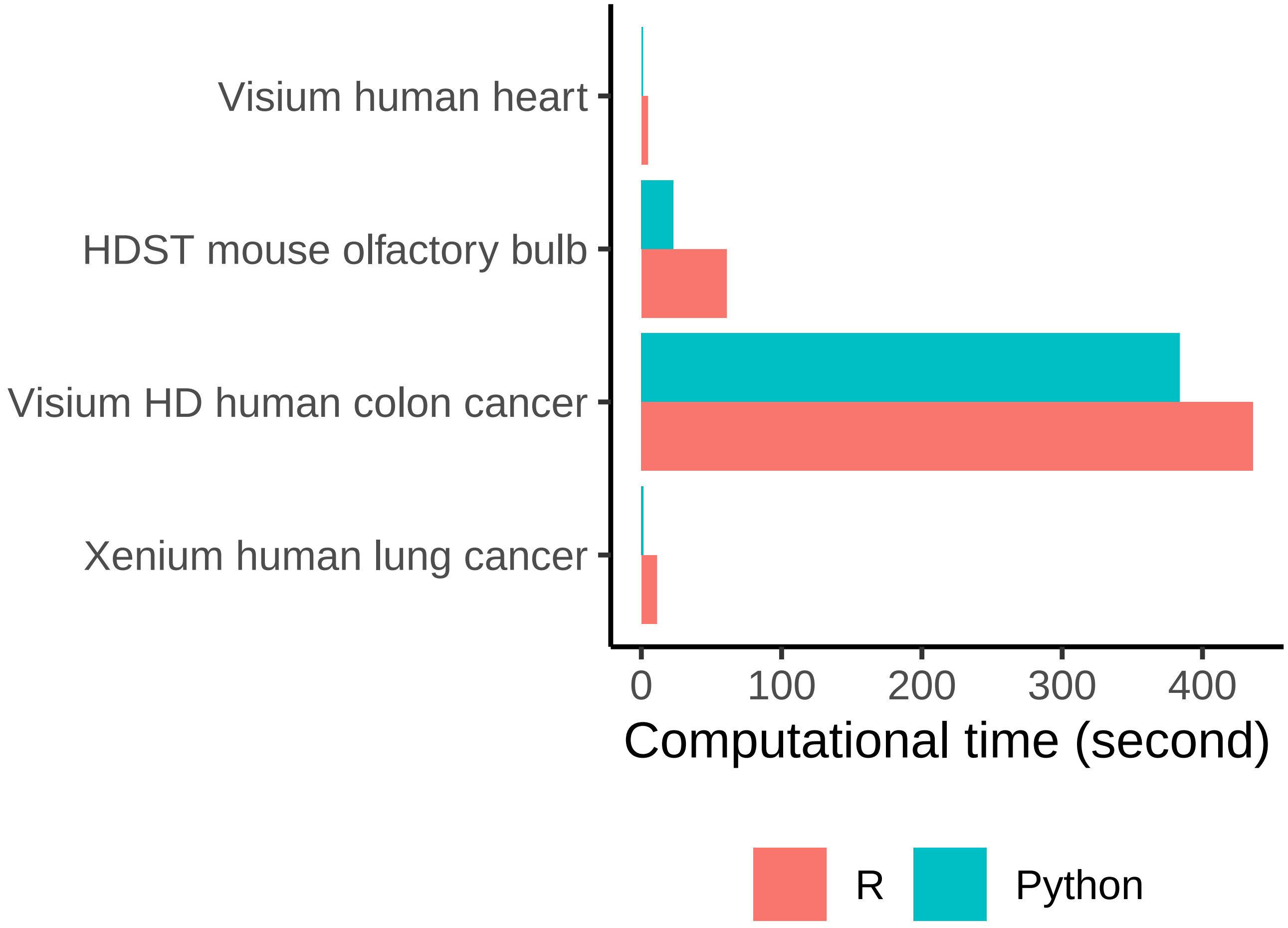

Fig. S36. Computational time of R and Python implementations of `PreTSA` in different datasets.

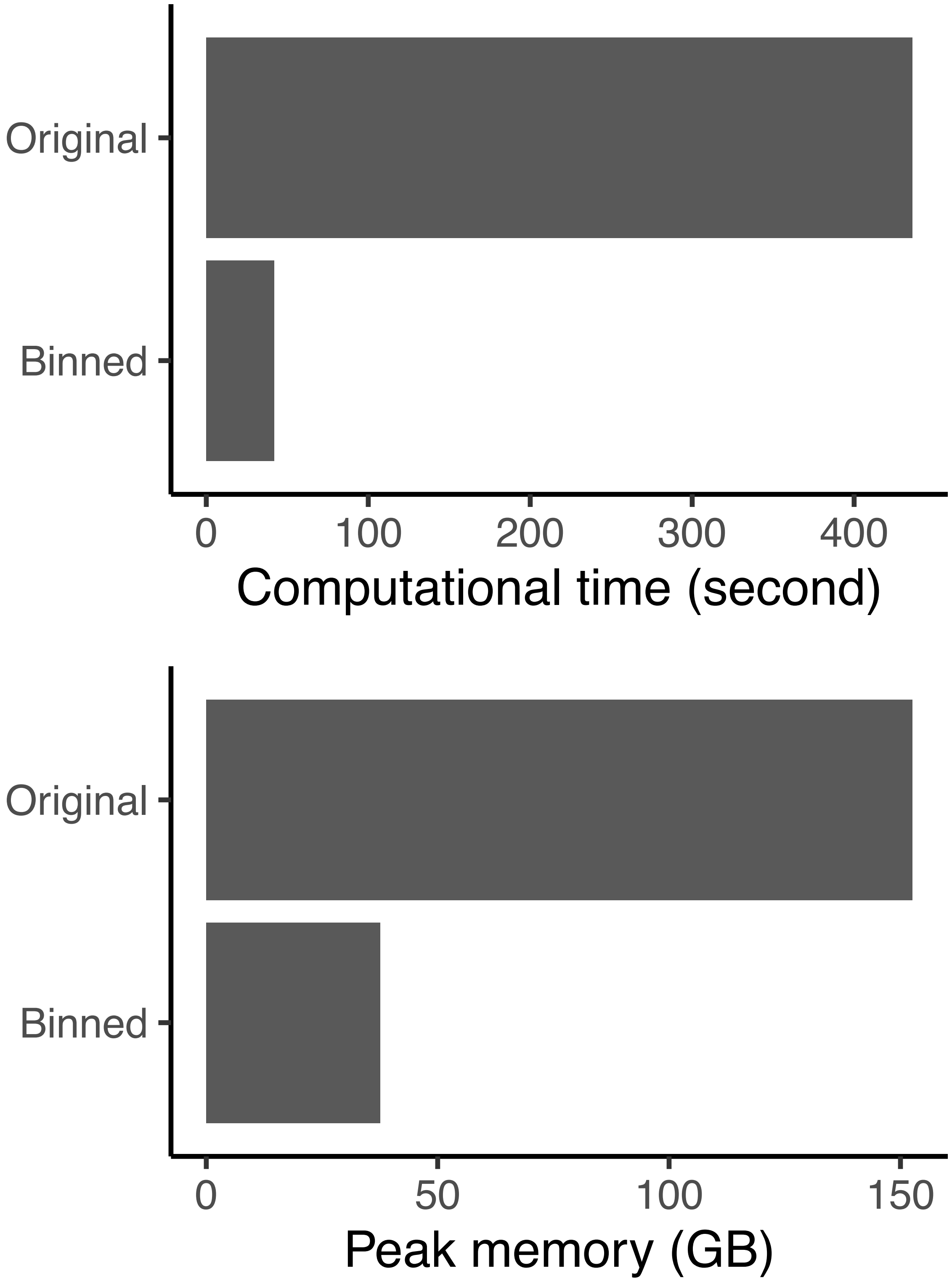

Fig. S37. Computational time (top) and peak memory usage (bottom) of `PreTSA` in the original and 10,000-binned Visium HD human colon cancer dataset.

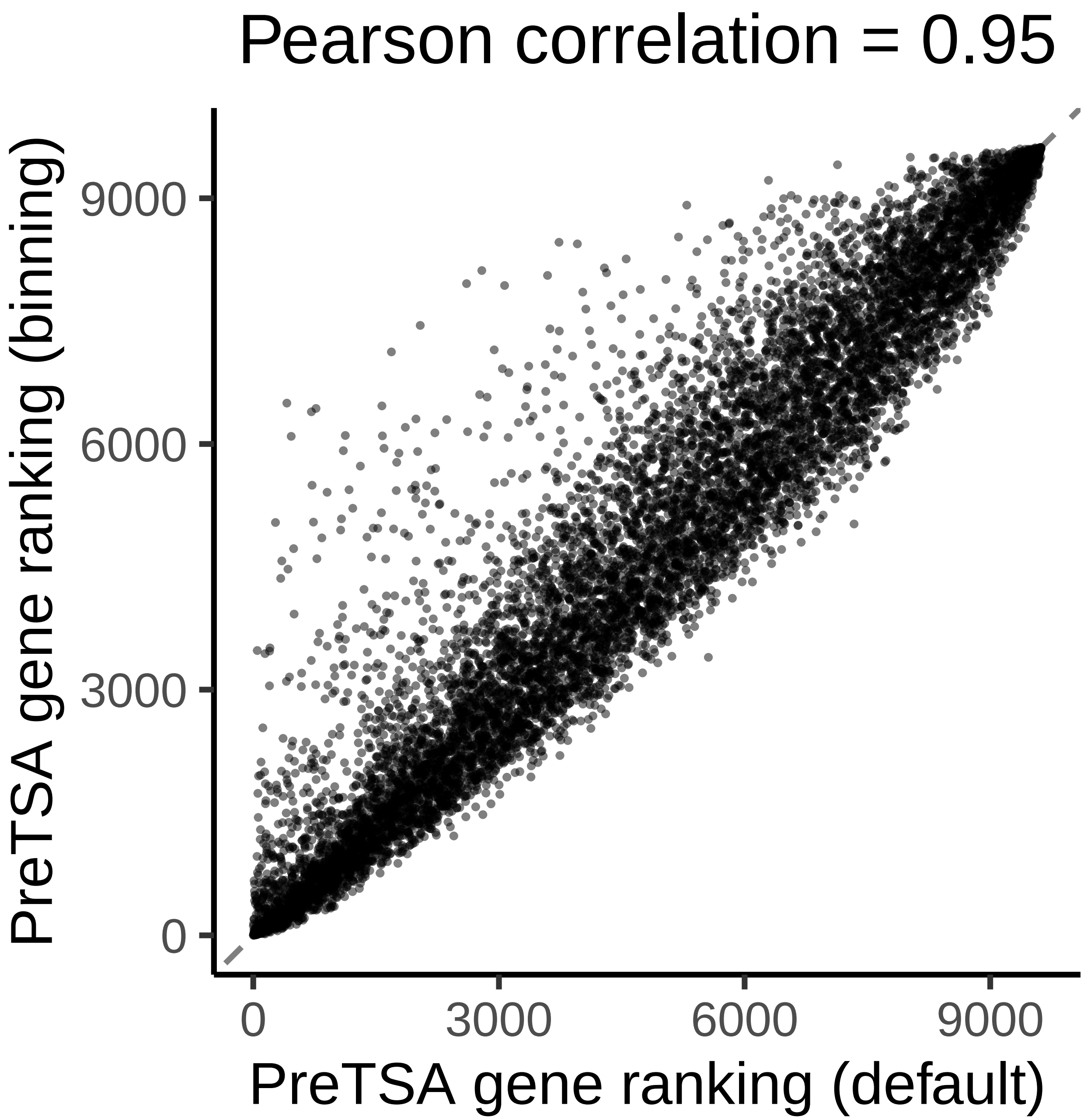

Fig. S38. Gene rankings for SVG identification in the Visium HD human colon cancer dataset, with default `PreTSA` on the x-axis and `PreTSA` using 10,000 bins on the y-axis.

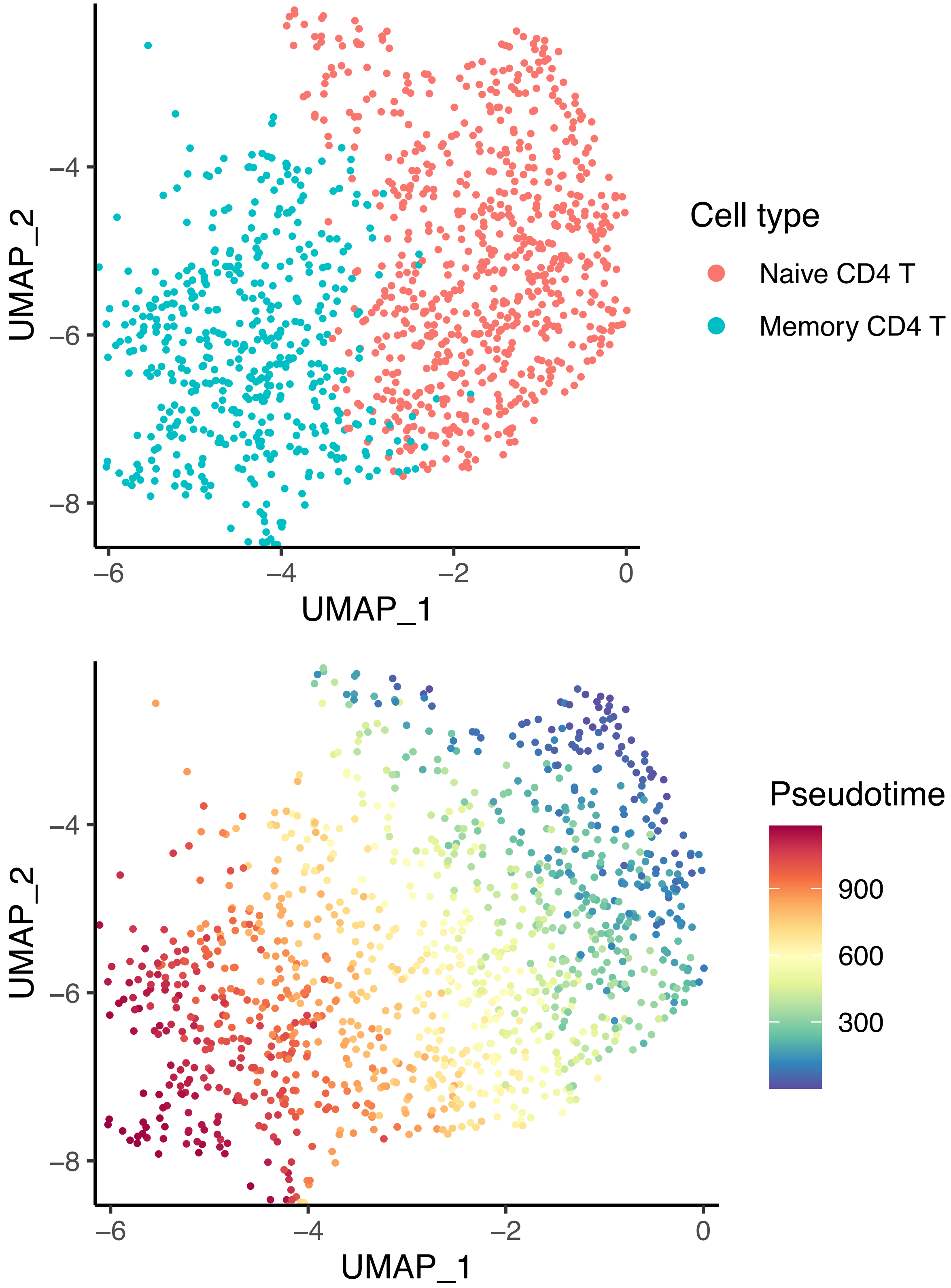

Fig. S39. UMAP plot colored by cell types (top) and pseudotime values (bottom) for the human PBMC scRNA-seq dataset.

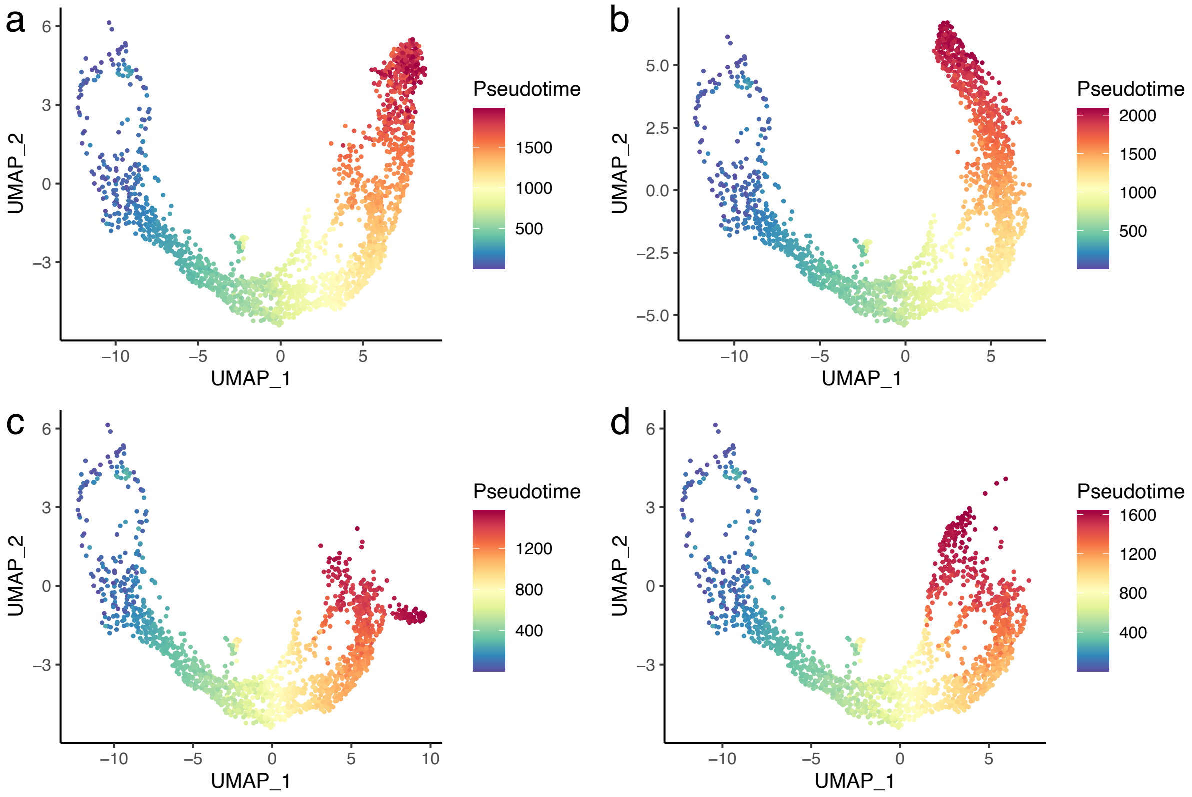

Fig. S40. UMAP plot colored by pseudotime values for the alpha branch (**a**), beta branch (**b**), delta branch (**c**), and epsilon branch (**d**) of the mouse pancreas scRNA-seq dataset.

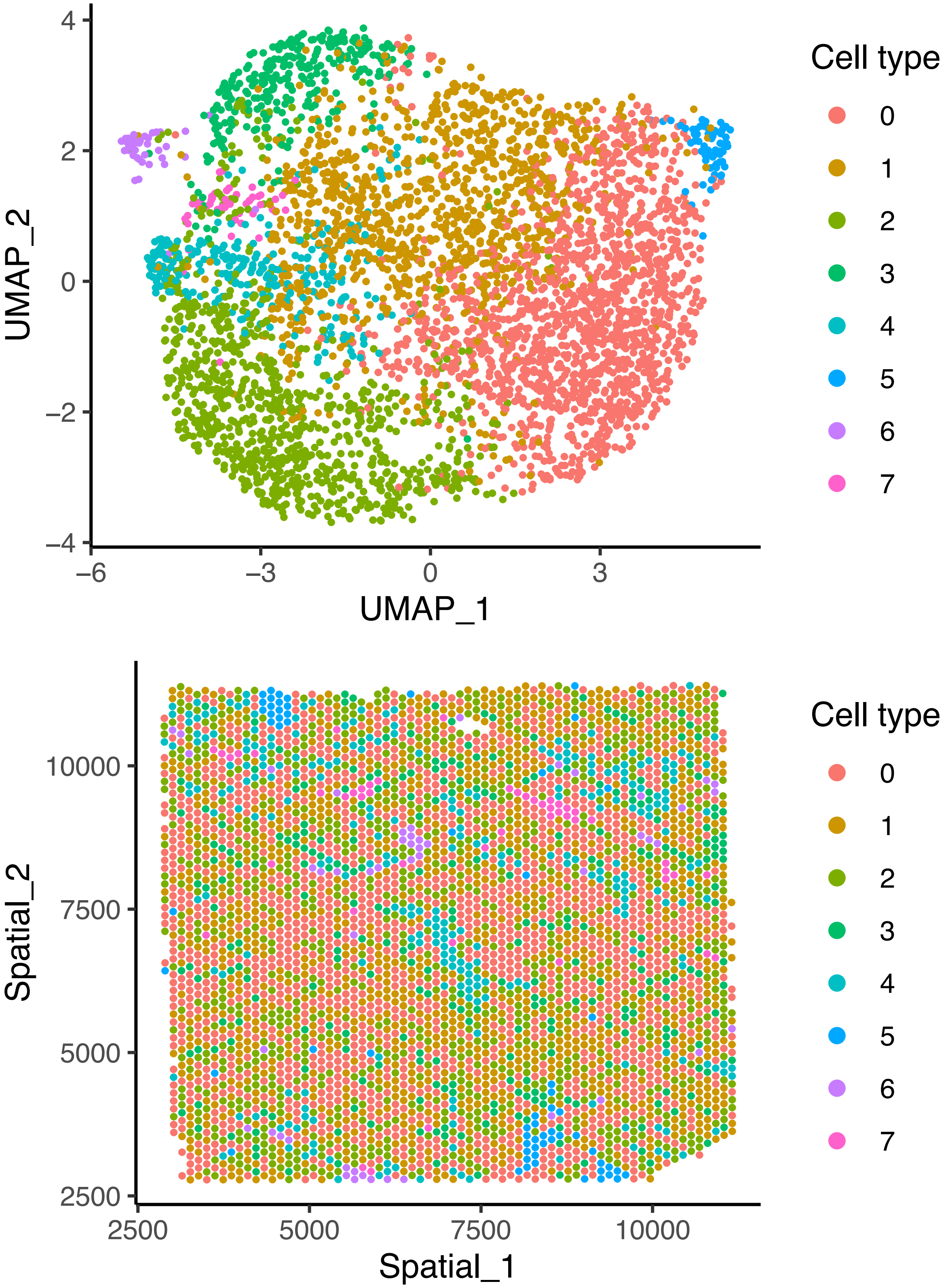

Fig. S41. UMAP plot (top) and spatial plot (bottom) colored by cell types for the Visium human heart dataset.

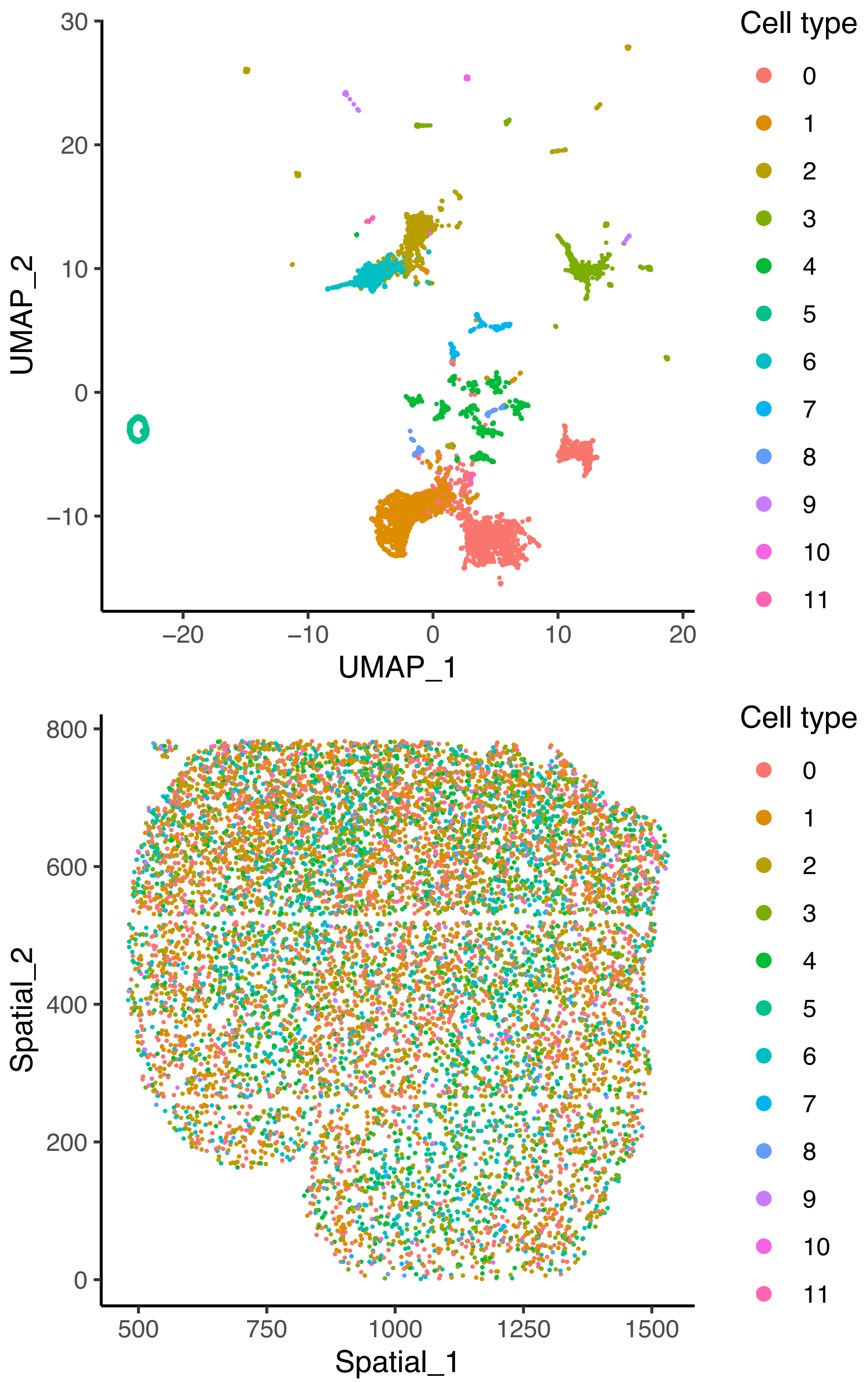

Fig. S42. UMAP plot (top) and spatial plot (bottom) colored by cell types for the HDST mouse olfactory bulb dataset.

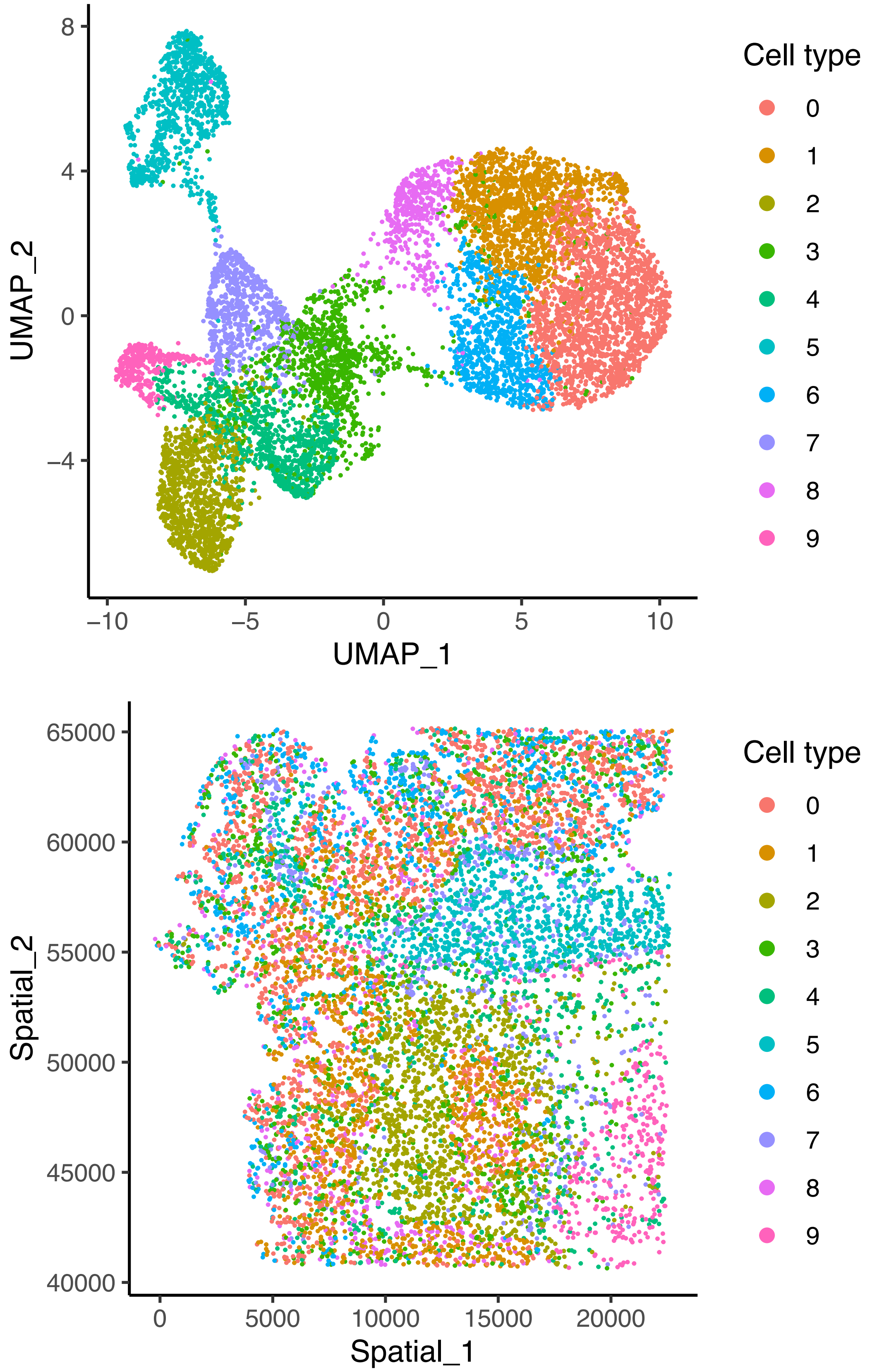

Fig. S43. UMAP plot (top) and spatial plot (bottom) colored by cell types for the Visium HD human colon cancer dataset.

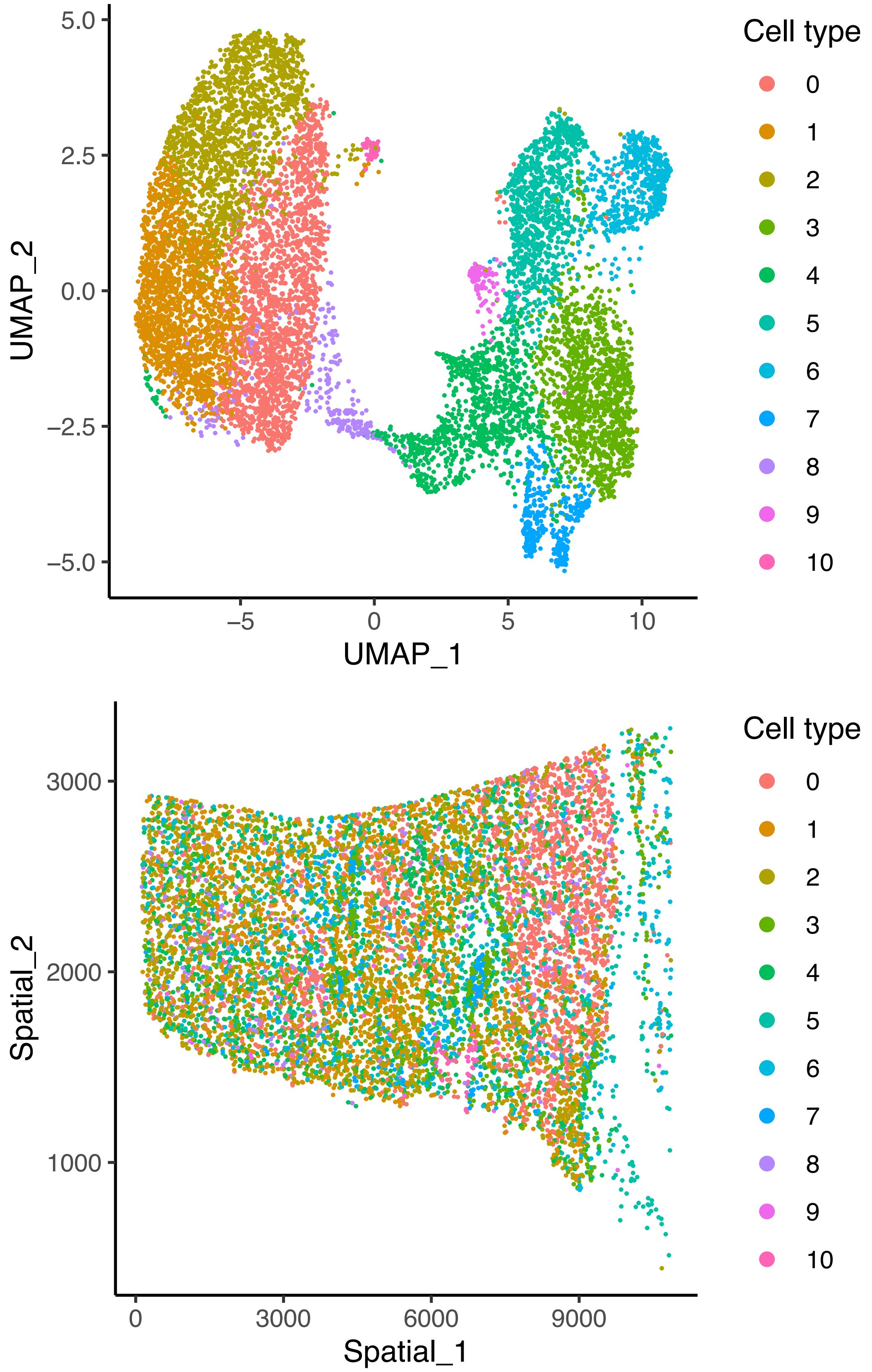

Fig. S44. UMAP plot (top) and spatial plot (bottom) colored by cell types for the Xenium human lung cancer dataset.
