# Supplementary material for: PreTSA: computationally efficient modeling of temporal and spatial gene expression patterns
Source: Genome Biol. 2026 Feb 12;27:93. doi: 10.1186/s13059-026-03994-3 (PMC12998178; doi:10.1186/s13059-026-03994-3)
Supplement: Supplementary file 2 — Additional file 2. Peer review history. [file 13059_2026_3994_MOESM2_ESM.docx]

Review History

**First round of review**

**Reviewer 1**

**Were you able to assess all statistics in the manuscript, including the appropriateness of statistical tests used?** There are no statistics in the manuscript.

**Were you able to directly test the methods?** No

**Comments to author:**

The manuscript presents PreTSA, a novel computational method designed to efficiently model temporal and spatial gene expression patterns in large-scale single-cell and spatial transcriptomics data. The authors highlight the limitations of current methods when dealing with large datasets comprising millions of cells and propose PreTSA as a solution that significantly improves computational efficiency without compromising the consistency of results. Although the efficiency is important, at this stage this is not the bottleneck. However, the authors need to address the following issues:

1. The core innovation of the manuscript is the improvement in computational efficiency. Although the authors have partially demonstrated the computational effectiveness of their method, they didn't provided enough experiments. As there is generally a trade-off between computational efficiency and performance, for the prestigious journal like Genome Biology, the authors should conduct more experiments to verify that the recommended method is effective across various tasks and outperforms baseline methods.

2. To assist users in understanding and utilizing the tool, the authors should provide detailed tutorials on GitHub. This would facilitate users in reproducing the results of the paper and in using the tool effectively.

3. Although PreTSA shows computational prowess, the manuscript must strengthen the discussion on the interpretability of the results. The authors are advised to provide more biological insights to demonstrate how PreTSA can elucidate complex gene expression dynamics.

4. A more thorough analysis of the robustness of the PreTSA model is needed, particularly concerning parameter selection and the determination of the number of internal knots. The authors should discuss the model's sensitivity to different parameter settings and offer guidance on selecting optimal parameters.

5. The analysis of PreTSA results in the article mainly focuses on the comparison with existing methods, while the analysis of the results of PreTSA itself is not in-depth enough. It is recommended that the author further analyze the TVGs and SVGs identified by PreTSA, such as their biological functions and roles in the process of cell differentiation.

6. The article only used a simulated dataset and a real spatial transcriptomics dataset to evaluate the performance of PreTSA. It is recommended that the author use more real datasets to further verify the effectiveness and universality of PreTSA.

7. The description of the PreTSA method in the article is not detailed enough, especially in the modeling of spatial gene expression patterns. It is recommended that the author describe in detail in the methods section how PreTSA uses the tensor product to extend the one-dimensional B-spline basis to two dimensions, and how to use the F-test to identify SVGs.

8. The authors should compare their method with some of the latest deep learning-based approaches. From my understanding, deep learning can be effectively scaled to datasets with millions of entries through batch training and testing, and it typically performs well. Therefore, I feel that the innovation of this paper is insufficient. The authors need to further substantiate the novelty of their work. Additionally, I suggest that the authors include comparisons with recent deep learning methods in their experiments to comprehensively evaluate the strengths and weaknesses of their approach. Demonstrating performance on larger datasets could more convincingly prove the effectiveness and uniqueness of their method.

**Reviewer 2**

**Were you able to assess all statistics in the manuscript, including the appropriateness of statistical tests used?** Yes, and I have assessed the statistics in my report.

**Were you able to directly test the methods?**

**Comments to author:**

The authors of the manuscript present PreTSA, a new computational method to fit gene expression in single cell and spatial transcriptomics data as separate continuous fields per gene on a manifold of dimension 1 or 2. The fields are parametrized by cubic B-splines for the 1D case and by a tensor product of cubic B-splines for the 2D case and fitted to the data using least squares. The manifolds are given as a mapping of observations (cells or pixels) to coordinates in a pre-computed pseudotime ordering of cells in the single cell transcriptomics case or as the measured spatial coordinates in the spatial transcriptomics case. Aside from the fitted smooth dependency of the expression on the continuous pseudotime or spatial coordinates, the method also provides temporally or spatially variable genes (TVGs or SVGs) as a derived result. The new method produces comparable results as pre-existing methods in the cases benchmarked by the authors. The main advance of PreTSA over pre-existing methods is its very small computational resource footprint, which enables its application to very large transcriptomics datasets which are increasingly available today.

The central aim of the study is to provide a computationally much more efficient method to fit gene expression fields and to find TVGs or SVGs. The authors implement such a method and provide benchmarking with previous methods to establish the claim of computational efficiency and to demonstrate similar results. This is in principle sufficient to demonstrate the computational performance gain and the validity of the new method. While method papers often also include more biological analyses to showcase new features of the method, new features aside from computational efficiency are not central to the manuscript, so biological analyses are not necessary in principal. However, the benchmarking lacks the breadth and depth necessary to sufficiently support the validity of the new method and the computational gains with respect to the population of previous methods on current compute hardware. Additionally, the method seems to have potentially severe undiscussed limitations (focus on tissue-scale spatial structure, assumptions of Gaussian distribution of count data and separability of the spatial distribution, support only for linear developmental trajectories).

Detailed comments:

There is only limited evidence provided that the results are valid, accurate, and biologically meaningful: increasing computational performance is of limited value if the quality of the results suffers. The authors recognize a conceptual difficulty to provide conclusive evidence and state that "it is hard to assess the quality of identified SVGs due to the lack of a gold standard and varying definitions of SVGs". While true, it is certainly possible to provide more indirect evidence, e.g. by including more structured comparisons to other published methods and, crucially, to include benchmarking with simulated data where there is a ground truth available. Including more published methods would also strengthen the claim of increased computational performance with respect to the population of pre-existing methods. In the current manuscript, the authors just compare performance to one independent method for the pseudo-time case (PseudotimeDE; the other one, GAM, is implemented by the authors).

The need of increased computational performance is motivated by the larger datasets generated with current experimental spatial transcriptomics methods. But in the spatial case, the manuscript only demonstrates PreTSA for (upsampled) original Visium data, which has great differences in data properties like sparsity, spatial resolution, and genome size compared to more current methods. In particular, a higher spatial resolution means that instead of averages over cellular communities individual cells can be resolved which leads to an additional set of spatially variable genes as spatial heterogeneity on much smaller spatial length scales is accessible. This ties into the point below regarding the spatial scales which PreTSA is applicable on.

Computational performance is the key point in the manuscript. Since decades computers got faster by increasing parallelism, in particular the number of cores, such that current CPUs have hundreds of cores. Still, the authors only compare single threaded execution. This leaves the possibility that PreTSA is the only method which cannot scale to many cores, while the other methods do have comparable performance if a full current CPU is used. In particular, if the competing methods work independently per gene as suggested in Fig 1a they should scale perfectly and gain a factor of 100 in speed if they are allowed to use a CPU with 100 cores. R is chosen as the main programming language for the implementation of PreTSA, which is a very unusual choice if computational performance is important as e.g. the support for running on parallel devices has historically been very inefficient - this makes the scenario that PreTSA just does not scale well to many cores likely. An implementation in C or jit-compiled python (e.g. based on jax or pytorch) should be quite straightforward for the PreTSA case (with K=0), scale nicely with multiple cores, and even add support for GPU.

Aside from computation time also memory requirements are of paramount importance for computational performance. How do the methods compare here?

Is the 100x execution for p-value computation in the PseudotimeDE-scheme included in the cited computation time for PreTSA?

How does the computational cost of the pseudotime inference compare to the cost of running PreTSA on top of these results?

Is the multiple testing for finding an optimal K with the BIC in the PreTSA-K model already included in the multiple testing correction?

As the authors write, the empirical p-value for the TVG determination captures the uncertainty in inferring the pseudotime, but at the cost of having to perform the potentially very expensive pseudotime inference 100 times. How does this compare to just using the computed F statistic for an F test, which would be 100x more efficient? As computational efficiency is the key contribution of this manuscript, this should be discussed.

The pseudotime comes often from methods for reconstructing developmental trajectories. These trajectories can have complicated topologies, e.g. branched or non-simply connected topologies. It seems that PreTSA is limited to non-branching line-segment like developmental trajectories. If so this limitation is crucial for practical applications in the pseudotime case.

The tensor product ansatz of cubic B splines for the spatial case implicitly restricts the functional space covered by the fit compared to full polynomials in x and y coordinates of degree 3: They constitute only separable functions in x and y, i.e. assume f(x,y)=g(x)*h(y)), although in general separability should not hold. In particular, this assumption breaks the rotational symmetry of 2D space into a discrete subgroup: not all directions are equivalent and the model cannot provide the same fit for data rotated by e.g. 45°. This introduces artefacts in the modelling which were not discussed, quantified, or remedied in the current manuscript. A possible artefact is a more axis-aligned result, evidence for which could be the PreTSA result shown in Fig 2g. For the PreTSA case (not the more general PreTSA-K), a solution which keeps the symmetry is very simple: just include the full basis of polynomials of degree 3, instead of the separable subset appearing in the tensor product.

Spatial scales are very relevant for the analysis of spatial data to analyze effects of whole tissues (global gradients within a tissue), tissue substructures (e.g., effects of distance to vascular structures), of cell neighbourship level (e.g. cell-cell communication effects) or even intracellular (e.g., reorganization of organellar structures). As going down to finer spatial scales would blow up the number of necessary internal nodes orders of magnitude beyond the proposed maximum of K=5, it seems that PreTSA only applies to the global scale, which is often confounded with tissue handling and/or imaging artefacts. This should drastically limit the practical relevance of the method for the spatial use case.

The model assumes Gaussian distribution of the expression data, which for count data is clearly not the case (as can be seen in Fig 1d). This apparent mismatch should be discussed. That this is important is indicated in the curious diversity of the SPARK-group of benchmarked methods: the most recent method with speed comparable to PreTSA, SPARK-X, was introduced as a non-parametric fix of the earlier parametric SPARK and SPARK-G models which assumed Poisson or Gaussian distributions. That the assumed distribution matters and has a strong impact on the results is also visualized by Figs 2j, S2c, S2d in the current manuscript: The three methods with the most similar results to PreTSA (GAM, SpatialDE, Spark-G) all assume Gaussian distribution.

Finally, a remark about the presentation: Fig 1b and 1c suggest that the right hand panels are zoomed versions of the left hand panels, which is not the case as GAM and PseudotimeDE are not represented in the right hand panels. At least the point with the smallest number of cells should still be visible in the zoomed version, as they have smaller computation time than the values of the largest number of cells of PreTSA-K. Also the time axis on the left hand panels is on the linear scale with a scale break and on the right hand panels on a log scale - it would help the reader to put both on a log scale.

**Reviewer 3**

**Were you able to assess all statistics in the manuscript, including the appropriateness of statistical tests used?** Yes, and I have assessed the statistics in my report.

**Were you able to directly test the methods?**

**Comments to author:**

===
The authors present PreTSA, a memory and time efficient method for modeling temporal and spatial gene expression. The results presented in this manuscript demonstrate that PreTSA has a better performance in terms of running time and memory usage at different dataset sizes in comparison with other available methods, while preserving comparable results when detecting Temporal and Spatial Variable Genes. I have a few comments that may benefit the manuscript.
===
- Although the correlation and overlapping analysis show a good agreement between PreTSA and previous methods for the detection of TVG and SVG, I believe the manuscript is laking plots that show the direct output from PreTSA when applying it to the mentioned real and simulated datasets (e.g., a UMAP of cells colored using the pseudotime vs a UMAP with cell types)
- While Fig. 2d-g suggests an interesting improvement in the detection of spatial patterns, the original expression shows a complex and subtle pattern that makes difficult to conclude the result is accurate. I suggest to include a second example where the original expression offers a cleared distribution.
- Fig. 2j shows the overlap proportion between PreTSA and other methods. I wonder how the spatial distribution of gene expression from the genes found using PreTSA but not detected on the other methods looks like. Do you observe particular patterns that explain why they are found using PreTSA but no with the other methods?
- I suggest including the website link in the Data availability section, currently is only visible when exploring the GitHub repository.

**Authors Response**

## Response to Reviewer 1

The manuscript presents PreTSA, a novel computational method designed to efficiently model tem-poral and spatial gene expression patterns in large-scale single-cell and spatial transcriptomics data. The authors highlight the limitations of current methods when dealing with large datasets comprising millions of cells and propose PreTSA as a solution that significantly improves computational efficiency without compromising the consistency of results. Although the efficiency is important, at this stage this is not the bottleneck. However, the authors need to address the following issues:

We sincerely thank the reviewer for the constructive feedback. We have carefully addressed all questions and comments in this revision, which we believe has substantially improved the manuscript. Our point-by-point responses to the comments are provided below.

1. The core innovation of the manuscript is the improvement in computational efficiency. Although the authors have partially demonstrated the computational effectiveness of their method, they didn’t provided enough experiments. As there is generally a trade-off between computational efficiency and performance, for the prestigious journal like Genome Biology, the authors should conduct more ex-periments to verify that the recommended method is effective across various tasks and outperforms baseline methods.

We thank the reviewer for the valuable comment. In the revised manuscript, we have incorporated a substantial number of additional experiments and analyses to further strengthen the study. Specifically, we have made the following updates:

- We added one additional real dataset for pseudotime analysis and two additional datasets for spatial analysis. The spatial datasets were generated by two genomic platforms distinct from the 10x Visium platform and include a much larger number of cells than the dataset used in the original manuscript, further demonstrating PreTSA’s efficiency on extremely large datasets.
- We included additional simulation datasets with ground truth for both pseudotime and spatial settings. The results show that PreTSA achieves among the best performance in correctly iden-tifying TVGs and SVGs while effectively controlling false positives in null simulations. These findings complement the main result that PreTSA substantially improves the computational efficiency of existing methods.
- We conducted an analysis of memory usage in addition to running time. The results indicate that PreTSA is not only computationally efficient but also among the most memory-efficient methods.
- We provided biological interpretation of the results obtained by PreTSA. For each real dataset, we performed GO enrichment analysis to reveal pathways associated with the top TVGs and SVGs, and we discussed the biological relevance of the genes identified.
- We added further analyses of the PreTSA method, which are included in the Discussion section of the revised manuscript.

### We have added Figures 1d, 1j–l, 2d, and 2m–o, as well as Supplementary Figures 1–6, 8–15, and 17–33, and have substantially revised the Results, Discussion, and Methods sections of the main manuscript.

2. To assist users in understanding and utilizing the tool, the authors should provide detailed tutorials on GitHub. This would facilitate users in reproducing the results of the paper and in using the tool effectively.

We thank the reviewer for the comment. We have provided detailed tutorials on GitHub, which are now available at <https://haotian-zhuang.github.io/PreTSA/> . These resources include installation instructions for the PreTSA package and two tutorials that demonstrate modeling temporal and spatial gene expression patterns separately. Each tutorial contains an introduction, step-by-step guidance for loading the necessary packages and datasets, procedures for fitting temporal or spatial gene expression patterns, methods for identifying TVGs or SVGs, and instructions for visualizing the fitted patterns.

### We have added the website link of the tutorials to the Data Availability section:

“ All datasets used in the study are publicly available. The R package PreTSA is publicly available at <https://github.com/haotian-zhuang/PreTSA>. Comprehensive tutorials are available at [https:](https://haotian-zhuang.github.io/PreTSA/)

[//haotian-zhuang.github.io/PreTSA/](https://haotian-zhuang.github.io/PreTSA/).

3. Although PreTSA shows computational prowess, the manuscript must strengthen the discussion on the interpretability of the results. The authors are advised to provide more biological insights to demonstrate how PreTSA can elucidate complex gene expression dynamics.

We thank the reviewer for this comment. In the revised manuscript, we have substantially enhanced the biological insights of the genes identified by PreTSA. Specifically, for each of the two pseudotime datasets and each of the three spatial datasets, we performed GO enrichment analysis and provided biological interpretations of how the top TVGs or SVGs are related to cell development and differen-tiation processes, or to the spatial organization of the tissue. The results suggest that the TVGs and SVGs identified by PreTSA are highly relevant to continuous biological processes and spatial patterns, and can be used to elucidate temporal and spatial gene expression dynamics.

### We have added Figure 1j-l, Figure 2m-o, Supplementary Figure 9,11-12, 18-19, 21-23, 25-27, and the following content to the Results section of the manuscript:

“ The top TVGs identified by PreTSA capture key biological functions underlying the cell develop-ment process. For instance, many enriched GO terms are associated with memory T cell activation, such as MHC protein complex assembly and response to interleukin-7 (Figure 1j). Among the top 20 TVGs (Supplementary Figure 9), *S100A4* exhibits an increasing temporal trend, consistent with its role in modulating T cell migration^21^ (Figure 1k). In contrast, the ribosomal gene *RPL13* shows a decreasing trend, reflecting reduced ribosome biogenesis during the transition to a quiescent memory state^1^ (Figure 1l).

Consistent with endocrine development, GO terms enriched among the TVGs identified by PreTSA include insulin metabolic process and dense core granule exocytosis (Supplementary Figure 11). Among the top TVGs identified by PreTSA, *Iapp*, a hormone co-secreted with insulin, shows an increasing trend^20^. *Chga*, which is involved in hormone processing, increases during endocrine differentiation and then slightly decreases as beta cells mature^3^. In addition, *Neurog3* rises initially as the master transcription factor initiating endocrine commitment, but decreases once its transient role is fulfilled as cells progress into the pre-endocrine state^3^^,^ ^19^ (Supplementary Figure 12).

Consistent with cardiac function, GO terms enriched among the SVGs identified by PreTSA include sarcomere organization, regulation of cardiac contraction force, and myofibril assembly (Figure 2m). Notably, *ACTA1* shows elevated expression in the bottom-right region of the tissue, suggesting cardiac remodeling^10^ (Figure 2n), whereas *MYL7* is enriched in the top-right region, likely corresponding to atrial cardiomyocytes^12^ (Figure 2o, Supplementary Figure 18-19).

The SVGs identified by PreTSA-K were enriched in key biological processes of the olfactory bulb, in-cluding ionotropic glutamate receptor signaling and SNARE complex assembly (Supplementary Figure 21). The fitted spatial expression patterns of top-ranked SVGs showed pronounced spatial organiza-tion consistent with known biology. For example, *Gphn* and *Camk1d*, which are critical for inhibitory synapse organization and calcium signaling, respectively, showed elevated expression in the external plexiform layer and glomerular layer^16^^,^ ^17^ (Supplementary Figure 22-23).

The SVGs identified by PreTSA-K were enriched in pathways such as antimicrobial humoral response and collagen fibril organization, reflecting the distinct epithelial and stromal programs characteristic of colon cancer tissue (Supplementary Figure 25). Consistent with these pathways, many of the top SVGs are well-established tumor-associated genes. For example, *MUC2*, which contributes to mucus barrier formation, showed higher expression in the epithelial goblet cell compartment^6^; *COL1A1*, encoding fibrillar collagens secreted by cancer-associated fibroblasts, was upregulated in the stromal compartment^23^; and *CEACAM6*, a tumor-associated adhesion molecule, was enriched in malignant glands^8^ (Supplementary Figure 26-27). ”

4. A more thorough analysis of the robustness of the PreTSA model is needed, particularly concern-ing parameter selection and the determination of the number of internal knots. The authors should discuss the model’s sensitivity to different parameter settings and offer guidance on selecting optimal parameters.

We thank the reviewer for the comment. In the revised manuscript, we performed a sensitivity analysis to compare the fitted gene expression profiles and gene rankings obtained by PreTSA under different numbers of internal knots. The results (Supplementary Figure 1-2) show that both the fitted gene expression and the gene rankings remain highly consistent across different settings, indicating that the PreTSA model is relatively robust to this parameter choice.

We recommend that users employ the automatic knot selection procedure (PreTSA-K) when aiming to balance computational efficiency with fitting accuracy, as PreTSA-K evaluates a set of candidate models and selects the optimal one to better capture spatial and temporal gene expression variation. When computational efficiency is the primary concern, we suggest using PreTSA directly without automatic knot selection.

### We have added Supplementary Figure 1-2, and the following discussions and guidelines to the Results section of the manuscript:

“ While PreTSA is robust to the number of knots (Supplementary Figure 1-2), users have the option to select a flexible mode, PreTSA-K, which automatically determines the number of knots in the B-spline basis (Methods). PreTSA is recommended for maximum computational efficiency, while PreTSA-K should be used when balancing efficiency with fitting accuracy. ”

5. The analysis of PreTSA results in the article mainly focuses on the comparison with existing meth-ods, while the analysis of the results of PreTSA itself is not in-depth enough. It is recommended that the author further analyze the TVGs and SVGs identified by PreTSA, such as their biological functions and roles in the process of cell differentiation.

We thank the reviewer for the comment. As noted in our response to Comment 3, we have substan-tially enhanced the interpretation of PreTSA results in the revised manuscript, including expanded GO analyses and discussion of the roles of specific genes in each real dataset.

6. The article only used a simulated dataset and a real spatial transcriptomics dataset to evaluate the performance of PreTSA. It is recommended that the author use more real datasets to further verify the effectiveness and universality of PreTSA.

We thank the reviewer for the comment. As noted in our response to Comment 1, we have incorporated one additional real dataset for pseudotime analysis and two additional real datasets for spatial analysis in the revised manuscript. Across all settings, the results consistently demonstrate that PreTSA remains the most computationally efficient method, even for extremely large spatial datasets.

### We have added the below contents to the Results section of the manuscript:

“ As another example, we applied PreTSA to study beta cell development during mouse pancreatic endocrinogenesis^3^. PreTSA and PreTSA-K completed the analysis in 8 and 18 minutes, respectively, whereas PseudotimeDE required 7.3 hours on a single core and 1.4 hours on 10 cores, in addition to

using more memory (Supplementary Figure 10). Consistent with endocrine development, GO terms enriched among the TVGs identified by PreTSA include insulin metabolic process and dense core granule exocytosis (Supplementary Figure 11). Among the top TVGs identified by PreTSA, *Iapp*, a hormone co-secreted with insulin, shows an increasing trend^20^. *Chga*, which is involved in hormone processing, increases during endocrine differentiation and then slightly decreases as beta cells mature^3^. In addition, *Neurog3* rises initially as the master transcription factor initiating endocrine commit-ment, but decreases once its transient role is fulfilled as cells progress into the pre-endocrine state^3^^,^ ^19^ (Supplementary Figure 12).

To further demonstrate the generality of PreTSA across different ST technologies, we applied it to a mouse olfactory bulb dataset generated by HDST^18^. After filtering, the dataset contained 10,729 genes and 181,135 spots. PreTSA, PreTSA-K, and SPARK-X each completed the analysis within 4 minutes, whereas other methods either required much longer runtimes or failed due to excessive memory usage (Supplementary Figure 20). The SVGs identified by PreTSA-K were enriched in key biological processes of the olfactory bulb, including ionotropic glutamate receptor signaling and SNARE complex assembly (Supplementary Figure 21). The fitted spatial expression patterns of top-ranked SVGs showed pro-nounced spatial organization consistent with known biology. For example, *Gphn* and *Camk1d*, which are critical for inhibitory synapse organization and calcium signaling, respectively, showed elevated expression in the external plexiform layer and glomerular layer^16^^,^ ^17^ (Supplementary Figure 22-23).

Finally, we applied PreTSA to identify SVGs in a human colorectal cancer (CRC) sample generated by the newly developed 10x Visium HD platform^9^. After filtering, the dataset contained 9,621 genes and 419,309 spots. PreTSA, PreTSA-K, and SPARK-X each completed the analysis within 30 minutes, whereas other methods either required substantially longer runtimes or failed due to excessive memory usage (Supplementary Figure 24). The SVGs identified by PreTSA-K were enriched in pathways such as antimicrobial humoral response and collagen fibril organization, reflecting the distinct epithelial and stromal programs characteristic of colon cancer tissue (Supplementary Figure 25). Consistent with these pathways, many of the top SVGs are well-established tumor-associated genes. For example, *MUC2*, which contributes to mucus barrier formation, showed higher expression in the epithelial goblet cell compartment^6^; *COL1A1*, encoding fibrillar collagens secreted by cancer-associated fibroblasts, was upregulated in the stromal compartment^23^; and *CEACAM6*, a tumor-associated adhesion molecule, was enriched in malignant glands^8^ (Supplementary Figure 26-27). ”

7. The description of the PreTSA method in the article is not detailed enough, especially in the mod-eling of spatial gene expression patterns. It is recommended that the author describe in detail in the methods section how PreTSA uses the tensor product to extend the one-dimensional B-spline basis to two dimensions, and how to use the F-test to identify SVGs.

We thank the reviewer for this helpful comment. In the previous version of the manuscript, we had already included details on how PreTSA employs the tensor product to extend the one-dimensional B-spline basis to two dimensions, as well as how the F-test is used to identify SVGs. In the revised manuscript, **we have rewritten the section describing the fitting of SVGs** using the tensor product of B-spline basis functions to improve clarity. For reference, the updated descriptions of the SVG fitting and F-test procedures are provided below.

“

### Fitting spatial gene expression patterns

Denote **Y** as the *m×n* gene expression matrix, with *m* representing number of genes and *n* representing number of cells or spots, and *y_ij_* as the expression level of gene *i* in cell or spot *j*. Denote **S***_j_* = (*s_j_*_1_*, s_j_*_2_) as the 2-dimensional spatial location of cell or spot *j*.

For each gene *i*, we define a smooth surface over spatial locations using a tensor product of univariate B-spline basis functions along each spatial axis:

iid

*y_ij_* = *f_i_*(*s_j_*_1_*, s_j_*_2_) + *ϵ_ij_,	ϵ_ij_ ∼ N* (0*, σ*^2^)*,*

*i*

*K*+3 *K*+3

*K*+3

*K*+3

*fi*(*sj*1*, sj*2) = X X *βi,k*1*,k*2 *b*1*,k*1 (*sj*1) *b*2*,k*2 (*sj*2) + X *βi,k*1*,*0 *b*1*,k*1 (*sj*1) + X *βi,*0*,k*2 *b*2*,k*2 (*sj*2) + *βi*0*.*

*k*1=1 *k*2=1

*k*1=1

*k*2=1

In this model, *b*_1_*_,k_*(*s*) and *b*_2_*_,k_*(*s*) denote the *k*th cubic B-spline basis functions for the first and second spatial dimensions, respectively. Each dimension includes *K* + 3 basis functions, assuming *K* internal knots. The term *b*_1_*_,k_*_1_ (*s_j_*_1_) *b*_2_*_,k_*_2_ (*s_j_*_2_) defines the tensor product basis, capturing interac-tions between the two spatial axes. The additive terms *β_i,k_*_1_*,*0 *b*_1_*_,k_*_1_ (*s_j_*_1_) and *β_i,_*_0_*_,k_*_2_ *b*_2_*_,k_*_2_ (*s_j_*_2_) represent marginal effects along each axis, and the scalar *β_i_*_0_ serves as the intercept. The full set of parameters, including the interaction coefficients *β_i,k_*_1_*,k*_2_ , the marginal coefficients *β_i,k_*_1_*,*0 and *β_i,_*_0_*_,k_*_2_ , the intercept *β_i_*_0_, and the variance *σ*^2^, are specific to each gene and estimated via least squares. This formulation yields a flexible and smooth spatial surface for gene expression, capturing both individual axis effects and their interactions across the two-dimensional tissue landscape.

*i*

For gene *i*, the regression model can be written in matrix form as follows: **Y***_i_* = **X*β****i* + ***ϵ****_i_*, where

	

1	*b*1*,*1(*s*11)	*· · · b*1*,K*+3(*s*11)	*b*2*,*1(*s*12)	*· · · b*2*,K*+3(*s*12)	*b*1*,*1(*s*11)*b*2*,*1(*s*12)	*· · ·	b*1*,K*+3(*s*11)*b*2*,K*+3(*s*12)

1	*b*1*,*1(*s*21)	*· · · b*1*,K*+3(*s*21)	*b*2*,*1(*s*22)	*· · · b*2*,K*+3(*s*22)	*b*1*,*1(*s*21)*b*2*,*1(*s*22)	*· · ·	b*1*,K*+3(*s*21)*b*2*,K*+3(*s*22)

	

**X** =	*.*



.

.

.

.



1 *b*1*,*1(*sn*1) *· · · b*1*,K*+3(*sn*1) *b*2*,*1(*sn*2) *· · · b*2*,K*+3(*sn*2) *b*1*,*1(*sn*1)*b*2*,*1(*sn*2) *· · · b*1*,K*+3(*sn*1)*b*2*,K*+3(*sn*2)

Similar to the case of modeling the temporal gene expression pattern, we can directly apply the hat matrix to the original gene expression matrix to obtain the fitted gene expression matrix **Y**ˆ :

**Y**ˆ = **YH**

Here *y*ˆ*_ij_* represents the fitted gene expression value of gene *i* in cell or spot *j*.

PreTSA chooses *K* = 0 for all genes. PreTSA-K chooses the optimal *K* using the same BIC procedure as the case of fitting temporal gene expression pattern. For a given *K* and a given gene *i*, BIC =

*n* log( ^1^ L.*n* (*y_ij_ − y*ˆ*_ij_*)^2^) + ((*K* + 4)^2^ + 1) log(*n*) + *n* + *n* log(2*π*).

*n*

*j*=1

By default, PreTSA-K chooses *K* from the set of integers ranging from 0 to 5.

### Testing for spatially variable genes (SVGs)

To test whether the expression level of gene *i* varies spatially, similar to the case of testing for TVGs, we define the following null and alternative hypotheses:

*H*0 : *βi,*1*,*0 = *. . .* = *βi,K*+3*,*0 = *βi,*0*,*1 = *. . .* = *βi,*0*,K*+3 = *βi,*1*,*1 = *. . .* = *βi,K*+3*,K*+3 = 0

*H*_1_ : At least one of the parameters in *H*_0_ is not zero

PreTSA uses the F-statistics:

*n j*=1

L.

L.

(*y*ˆ*_ij_ − y*¯*_i_*)^2^*/*((*K* + 4)^2^ *−* 1)

*F_i_* =	*_n_*

*j*=1

(*y_ij_*

*− y*ˆ*_ij_*

)^2^*/*(*n −* (*K* + 4)^2^)

which follows an exact F-distribution with ((*K* + 4)^2^ *−* 1*, n −* (*K* + 4)^2^) degrees of freedom under

*H*_0_.

Here *y*¯*_i_* = ^1^ L.*n	y_ij_*. All *p*-values are then adjusted for multiple testing using the BH procedure^4^.

*n	j*=1

”

In addition, **we have provided references** to better guide readers to the corresponding sections of the Methods:

“ Similarly, GAM can be extended with tensor products (Methods). ”

8. The authors should compare their method with some of the latest deep learning-based approaches. From my understanding, deep learning can be effectively scaled to datasets with millions of entries through batch training and testing, and it typically performs well. Therefore, I feel that the innovation of this paper is insufficient. The authors need to further substantiate the novelty of their work. Ad-ditionally, I suggest that the authors include comparisons with recent deep learning methods in their experiments to comprehensively evaluate the strengths and weaknesses of their approach. Demonstrat-ing performance on larger datasets could more convincingly prove the effectiveness and uniqueness of their method.

We thank the reviewer for this helpful comment. We would like to emphasize that beyond the identification of TVGs and SVGs, there are many other important tasks in the analysis of single-cell and spatial genomic data. The primary objectives of most existing deep learning–based methods are focused on tasks other than TVG and SVG identification. For example, several deep learning–based approaches, including SpaGCN, SpaSEG, DeepGFT, and SPACEL, have been specifically developed for spatial domain detection, which is a common and well-recognized goal in the analysis of spatial transcriptomics data. These models are powerful in uncovering tissue architecture and domain-specific expression patterns, but they are not designed for, nor can they be readily adapted to, the task of identifying SVGs.

To the best of our knowledge, no existing deep learning–based methods have been proposed that can be directly applied to identify either TVGs or SVGs. This observation highlights a current gap in the field. While we fully recognize and agree with the reviewer about the great potential of deep learning methods in advancing single-cell and spatial genomics, the lack of existing approaches targeting TVG and SVG identification prevents us from including such methods in our comparisons. We believe this further underscores the novelty and importance of developing methods like PreTSA that are specifically designed for these tasks.

As discussed above, in the revised manuscript we have included several spatial transcriptomics datasets that are substantially larger than the Visium dataset. The results demonstrate that PreTSA remains the most computationally efficient method, whereas many existing approaches fail to complete the computation within a reasonable time. These findings further highlight the advantage of PreTSA for identifying SVGs in extremely large-scale spatial transcriptomics datasets.

To further address this point, **we have expanded the Conclusions section of the manuscript**

to include a discussion on the potential role of deep learning methods in SVG identification:

“ PreTSA, like other existing approaches for identifying SVGs, is grounded in statistical modeling. In parallel, a number of deep learning–based methods have been developed for analyzing ST data, but their primary focus has been on tasks such as spatial domain detection rather than SVG identification. Examples include SpaGCN^7^, SpaSEG^2^, DeepGFT^15^, and SPACEL^22^, which are effective for uncovering tissue architecture and domain-specific expression patterns but are not designed for detecting SVGs. While deep learning methods are often praised for their scalability and efficiency in handling large-scale data, it remains unknown whether such approaches can be successfully adapted to the task of SVG identification. Exploring this possibility represents an interesting avenue for future research. ”

## Response to Reviewer 2

The authors of the manuscript present PreTSA, a new computational method to fit gene expression in single cell and spatial transcriptomics data as separate continuous fields per gene on a manifold of dimension 1 or 2. The fields are parametrized by cubic B-splines for the 1D case and by a tensor product of cubic B-splines for the 2D case and fitted to the data using least squares. The manifolds are given as a mapping of observations (cells or pixels) to coordinates in a pre-computed pseudotime ordering of cells in the single cell transcriptomics case or as the measured spatial coordinates in the spatial transcriptomics case. Aside from the fitted smooth dependency of the expression on the continuous pseudotime or spatial coordinates, the method also provides temporally or spatially variable genes (TVGs or SVGs) as a derived result. The new method produces comparable results as pre-existing methods in the cases benchmarked by the authors. The main advance of PreTSA over pre-existing methods is its very small computational resource footprint, which enables its application to very large transcriptomics datasets which are increasingly available today.

The central aim of the study is to provide a computationally much more efficient method to fit gene expression fields and to find TVGs or SVGs. The authors implement such a method and provide benchmarking with previous methods to establish the claim of computational efficiency and to demon-strate similar results. This is in principle sufficient to demonstrate the computational performance gain and the validity of the new method. While method papers often also include more biological analyses to showcase new features of the method, new features aside from computational efficiency are not central to the manuscript, so biological analyses are not necessary in principal. However, the benchmarking lacks the breadth and depth necessary to sufficiently support the validity of the new method and the computational gains with respect to the population of previous methods on current compute hardware. Additionally, the method seems to have potentially severe undiscussed limitations (focus on tissue-scale spatial structure, assumptions of Gaussian distribution of count data and sepa-rability of the spatial distribution, support only for linear developmental trajectories).

We sincerely thank the reviewer for the constructive feedback. We have carefully addressed all questions and comments in this revision, which we believe has substantially improved the manuscript. Our point-by-point responses to the comments are provided below.

1. There is only limited evidence provided that the results are valid, accurate, and biologically mean-ingful: increasing computational performance is of limited value if the quality of the results suffers. The authors recognize a conceptual difficulty to provide conclusive evidence and state that ”it is hard to assess the quality of identified SVGs due to the lack of a gold standard and varying definitions of SVGs”. While true, it is certainly possible to provide more indirect evidence, e.g. by including more structured comparisons to other published methods and, crucially, to include benchmarking with simulated data where there is a ground truth available. Including more published methods would also strengthen the claim of increased computational performance with respect to the population of pre-existing methods. In the current manuscript, the authors just compare performance to one independent method for the pseudo-time case (PseudotimeDE; the other one, GAM, is implemented by the authors).

We thank the reviewer for the insightful comment. We agree that a method with improved com-putational efficiency must also demonstrate reliable accuracy. In the revised manuscript, we have added simulation studies to systematically evaluate and compare the accuracy of PreTSA and existing methods in identifying both TVGs and SVGs.

Two types of simulation studies were conducted. In the first, we used scDesign3 to generate realistic pseudotime and spatial datasets with ground truth TVGs and SVGs. We then applied PreTSA and other methods to these datasets and compared the identified genes with the ground truth, thereby assessing each method’s ability to accurately recover TVGs and SVGs. In the second, we randomly permuted the pseudotime values and spatial coordinates of cells from real datasets to construct null datasets, and applied PreTSA and other methods. Since no true TVGs or SVGs are expected after permutation, any identified genes represent false positives. This analysis therefore evaluates each method’s ability to control false discoveries.

In the first type of simulation, PreTSA ranked among the top-performing methods for both TVG and SVG identification. In the second, PreTSA detected virtually no TVGs or SVGs in null datasets.

Together, these results demonstrate that PreTSA not only achieves high accuracy in identifying TVGs and SVGs but also effectively controls false positives.

To demonstrate that the TVGs and SVGs identified by PreTSA are biologically meaningful, we have substantially enhanced the biological interpretation of real datasets in the revised manuscript. Specifically, we added GO enrichment analyses for the top TVGs and SVGs, and provided detailed biological interpretations of selected top genes. These results suggest that the identified TVGs and SVGs are highly relevant to cellular differentiation processes and the spatial organization of tissues.

For the competing methods in pseudotime analysis, we found that most existing approaches rely on fitting generalized additive models (GAMs), though their implementations differ. Specifically, Monocle uses the VGAM R package, whereas TSCAN employs the mgcv R package for GAM fitting. In the revised manuscript, we adopted the original implementations from Monocle and TSCAN and treated them as two distinct pseudotime fitting methods. The results show that while these two implementations yield similar performance, PreTSA is substantially more computationally efficient than both.

### We have added or revised Figure 1d, 1j-l, 2d, 2m-o, Supplementary Figure 6-7, 9, 11-12, 15, 18-19, 21-23, 25-27 and updated the Results section of the manuscript as follows:

“ We included two different implementations of GAM, as used in Monocle and TSCAN, in the comparison.

For analyzing one million cells, both versions of GAM required more than 70 hours, while PreTSA

required only 2 minutes.

PreTSA also demonstrated improved accuracy in identifying TVGs, achieving a higher area under the precision–recall curve (AUPRC) on a simulation dataset generated by scDesign3^14^ with ground truth TVGs (Figure 1d). Moreover, when applied to a null simulation dataset with permuted pseu-dotime values, PreTSA identified almost no TVGs, indicating its ability to effectively control false positives (Supplementary Figure 6).

The top TVGs identified by PreTSA capture key biological functions underlying the cell development process. For instance, many enriched GO terms are associated with memory T cell activation, such as MHC protein complex assembly and response to interleukin-7 (Figure 1j). Among the top 20 TVGs (Supplementary Figure 9), *S100A4* exhibits an increasing temporal trend, consistent with its role in modulating T cell migration^21^ (Figure 1k). In contrast, the ribosomal gene *RPL13* shows a decreasing trend, reflecting reduced ribosome biogenesis during the transition to a quiescent memory state^1^ (Figure 1l).

Consistent with endocrine development, GO terms enriched among the TVGs identified by PreTSA include insulin metabolic process and dense core granule exocytosis (Supplementary Figure 11). Among the top TVGs identified by PreTSA, *Iapp*, a hormone co-secreted with insulin, shows an increasing trend^20^. *Chga*, which is involved in hormone processing, increases during endocrine differentiation and then slightly decreases as beta cells mature^3^. In addition, *Neurog3* rises initially as the master transcription factor initiating endocrine commitment, but decreases once its transient role is fulfilled as cells progress into the pre-endocrine state^3^^,^ ^19^ (Supplementary Figure 12).

Similar to the case of TVG identification, PreTSA achieved one of the highest AUPRCs for SVG identification on an scDesign3 simulation dataset (Figure 2d) and detected almost no SVGs on a null simulation dataset (Supplementary Figure 15).

Consistent with cardiac function, GO terms enriched among the SVGs identified by PreTSA include sarcomere organization, regulation of cardiac contraction force, and myofibril assembly (Figure 2m). Notably, *ACTA1* shows elevated expression in the bottom-right region of the tissue, suggesting cardiac remodeling^10^ (Figure 2n), whereas *MYL7* is enriched in the top-right region, likely corresponding to atrial cardiomyocytes^12^ (Figure 2o, Supplementary Figure 18-19).

The SVGs identified by PreTSA-K were enriched in key biological processes of the olfactory bulb, in-cluding ionotropic glutamate receptor signaling and SNARE complex assembly (Supplementary Figure 21). The fitted spatial expression patterns of top-ranked SVGs showed pronounced spatial organiza-tion consistent with known biology. For example, *Gphn* and *Camk1d*, which are critical for inhibitory synapse organization and calcium signaling, respectively, showed elevated expression in the external plexiform layer and glomerular layer^16^^,^ ^17^ (Supplementary Figure 22-23).

The SVGs identified by PreTSA-K were enriched in pathways such as antimicrobial humoral response and collagen fibril organization, reflecting the distinct epithelial and stromal programs characteristic

of colon cancer tissue (Supplementary Figure 25). Consistent with these pathways, many of the top SVGs are well-established tumor-associated genes. For example, *MUC2*, which contributes to mucus barrier formation, showed higher expression in the epithelial goblet cell compartment^6^; *COL1A1*, encoding fibrillar collagens secreted by cancer-associated fibroblasts, was upregulated in the stromal compartment^23^; and *CEACAM6*, a tumor-associated adhesion molecule, was enriched in malignant glands^8^ (Supplementary Figure 26-27). ”

2. The need of increased computational performance is motivated by the larger datasets generated with current experimental spatial transcriptomics methods. But in the spatial case, the manuscript only demonstrates PreTSA for (upsampled) original Visium data, which has great differences in data properties like sparsity, spatial resolution, and genome size compared to more current methods. In par-ticular, a higher spatial resolution means that instead of averages over cellular communities individual cells can be resolved which leads to an additional set of spatially variable genes as spatial heterogeneity on much smaller spatial length scales is accessible. This ties into the point below regarding the spatial scales which PreTSA is applicable on.

We thank the reviewer for the comment and acknowledge that real datasets generated by the latest ST technologies may differ substantially in data distribution from the upsampled Visium dataset. In the revised manuscript, we added two new spatial datasets generated by HDST and Visium HD, both of which are distinct from the 10x Visium platform. The two datasets contain 181,135 and 419,309 spots, respectively, which are both substantially larger than the datasets generated by 10x Visium. For each dataset, we evaluated computational efficiency in terms of running time and memory usage, and provided biological interpretations through GO enrichment analysis and functional characterization of the top SVGs. Consistent with the findings from the 10x Visium dataset in the original manuscript, these new results suggest that SPARK-X and PreTSA are the only two methods capable of completing the computation within a reasonable time.

### We have added Supplementary Figure 20-27 and following discussions to the Results section of the revised manuscript:

“ To further demonstrate the generality of PreTSA across different ST technologies, we applied it to a mouse olfactory bulb dataset generated by HDST^18^. After filtering, the dataset contained 10,729 genes and 181,135 spots. PreTSA, PreTSA-K, and SPARK-X each completed the analysis within 4 minutes, whereas other methods either required much longer runtimes or failed due to excessive memory usage (Supplementary Figure 20). The SVGs identified by PreTSA-K were enriched in key biological processes of the olfactory bulb, including ionotropic glutamate receptor signaling and SNARE complex assembly (Supplementary Figure 21). The fitted spatial expression patterns of top-ranked SVGs showed pronounced spatial organization consistent with known biology. For example, *Gphn* and *Camk1d*, which are critical for inhibitory synapse organization and calcium signaling, respectively, showed elevated expression in the external plexiform layer and glomerular layer^16^^,^ ^17^ (Supplementary Figure 22-23).

Finally, we applied PreTSA to identify SVGs in a human colorectal cancer (CRC) sample generated by the newly developed 10x Visium HD platform^9^. After filtering, the dataset contained 9,621 genes and 419,309 spots. PreTSA, PreTSA-K, and SPARK-X each completed the analysis within 30 minutes, whereas other methods either required substantially longer runtimes or failed due to excessive memory usage (Supplementary Figure 24). The SVGs identified by PreTSA-K were enriched in pathways such as antimicrobial humoral response and collagen fibril organization, reflecting the distinct epithelial and stromal programs characteristic of colon cancer tissue (Supplementary Figure 25). Consistent with these pathways, many of the top SVGs are well-established tumor-associated genes. For example, *MUC2*, which contributes to mucus barrier formation, showed higher expression in the epithelial goblet cell compartment^6^; *COL1A1*, encoding fibrillar collagens secreted by cancer-associated fibroblasts, was upregulated in the stromal compartment^23^; and *CEACAM6*, a tumor-associated adhesion molecule, was enriched in malignant glands^8^ (Supplementary Figure 26-27). ”

3. Computational performance is the key point in the manuscript. Since decades computers got faster by increasing parallelism, in particular the number of cores, such that current CPUs have hundreds

of cores. Still, the authors only compare single threaded execution. This leaves the possibility that PreTSA is the only method which cannot scale to many cores, while the other methods do have com-parable performance if a full current CPU is used. In particular, if the competing methods work independently per gene as suggested in Fig 1a they should scale perfectly and gain a factor of 100 in speed if they are allowed to use a CPU with 100 cores. R is chosen as the main programming language for the implementation of PreTSA, which is a very unusual choice if computational per-formance is important as e.g. the support for running on parallel devices has historically been very inefficient - this makes the scenario that PreTSA just does not scale well to many cores likely. An implementation in C or jit-compiled python (e.g. based on jax or pytorch) should be quite straightfor-ward for the PreTSA case (with K=0), scale nicely with multiple cores, and even add support for GPU.

We thank the reviewer for the comment. We fully agree that parallelization can improve the computational efficiency of some algorithms. However, we emphasize that parallelization comes with costs. Although modern CPUs have multiple cores, memory resources are typically limited. Paral-lelization often increases memory usage because multiple processes or threads may duplicate data, create temporary buffers, or hold intermediate results. Given the large size of single-cell and spatial data generated by contemporary sequencing platforms, using many cores can easily exceed the memory capacity typically available to research groups.

To illustrate this, we reran SPARK, SPARK-X, PseudotimeDE, and nnSVG, four packages that support parallelization, on all simulated and real pseudotime and spatial datasets using both 1 core and 10 cores. Other pseudotime or spatial methods were not tested in parallel since they do not support it. The results show that while parallelization substantially reduced computational time, it also greatly increased memory usage, failing to handle datasets requiring over 400 GB of memory that PreTSA can process with less than 100 GB. Most importantly, even with parallelization, these methods remained slower than PreTSA. We did not test larger numbers of cores for two reasons: (i) such runs would exceed the resource limits of our computing cluster, and (ii) increasing cores rarely yields proportional speedup. For example, using 100 CPUs generally results in much less than a 100-fold improvement due to non-parallelizable components and parallelization overhead.

Finally, we note that PreTSA performs computations across all genes simultaneously through efficient matrix operations, which limits the potential benefit of further parallelization. In contrast, parallelization is more useful for algorithms that operate on genes iteratively. While R’s parallelization efficiency may be lower than in some other languages, this is not a major concern for PreTSA. Moreover, it is not unusual for high-performance methods in this field to be implemented in R. For instance, SPARK-X and nnSVG, both designed for scalability and computational efficiency, are also implemented in R.

### We have included the multi-core performance of PseudotimeDE, SPARK, SPARK-X, and nnSVG in Figure 1c, 2j and Supplementary Figure 14, 17, 20, 24, and added the following discussions in the main manuscript:

“ While PseudotimeDE with 10 cores substantially reduced computation time, it also required much larger memory and failed to process a dataset of 600,000 cells using 400 GB of memory.

Note that SPARK, SPARK-X, and nnSVG provide parallelization options. Although parallelization substantially reduces computation time, SPARK and nnSVG still require considerably more time than PreTSA and PreTSA-K. Moreover, their memory usage increases markedly with parallelization, pre-venting them from handling datasets with 100,000 and 1,000,000 spots, respectively, under a 400 GB memory constraint. ”

In response to the reviewer’s suggestion, **we also developed a Python version of PreTSA**, which is available on GitHub at: <https://github.com/haotian-zhuang/PreTSA/tree/main/python>.

4. Aside from computation time also memory requirements are of paramount importance for compu-tational performance. How do the methods compare here?

We thank the reviewer for the comment. We fully agree that memory usage is an important factor to consider when evaluating computational efficiency. In the revised manuscript, we have reported the memory usage of PreTSA, PreTSA-K, and all existing methods (using both single-core and multi-core settings) across all simulation and real datasets. The results show that PreTSA and PreTSA-K are

among the most memory-efficient methods in most scenarios. Thus, PreTSA is efficient in terms of both running time and memory usage.

### We have added Supplementary Figure 4, 5, 8, 10, 13, 14, 17, 20, 24, and the following discussions to the Results section of the manuscript:

“ PreTSA and PreTSA-K also required less memory than the GAM implementations (Supplementary Figure 4).

While PseudotimeDE with 10 cores substantially reduced computation time, it also required much larger memory and failed to process a dataset of 600,000 cells using 400 GB of memory.

whereas PreTSA took only three minutes and required less memory (Figure 1g, Supplementary Figure 8)

whereas PseudotimeDE required 7.3 hours on a single core and 1.4 hours on 10 cores, in addition to using more memory (Supplementary Figure 10).

while PreTSA required only 7 minutes and less memory (Supplementary Figure 13)

Moreover, their memory usage increases markedly with parallelization, preventing them from han-dling datasets with 100,000 and 1,000,000 spots, respectively, under a 400 GB memory constraint (Supplementary Figure 14). ”

5. Is the 100x execution for p-value computation in the PseudotimeDE-scheme included in the cited computation time for PreTSA?

We thank the reviewer for this comment and apologize for the lack of clarity. The reported com-putation time for PreTSA includes the 100 executions required for p-value computation under the PseudotimeDE scheme. In the revision, **we have added the following sentence to the Results section** for further clarification:

“ A similar disparity in computational efficiency was observed between PreTSA and PseudotimeDE in identifying TVGs, with both methods relying on permutation procedures involving 100 repetitions of fitting on subsampled data. ”

6. How does the computational cost of the pseudotime inference compare to the cost of running PreTSA on top of these results?

We thank the reviewer for this comment. In our analyses, trajectory inference was performed with TSCAN, a computationally efficient method that completed in minutes for both datasets for pseudotime analysis. Computational cost varies widely across trajectory methods, with some requiring minutes and others days. Because PreTSA takes a pseudotemporal ordering as input, the runtime of trajectory inference itself is outside the scope of this study. In practice, the total time required to characterize gene expression dynamics from a raw expression matrix should include both trajectory inference and downstream modeling of temporal patterns with PreTSA.

### We have added the following content to the Discussion section of the manuscript:

“ The computational efficiency of trajectory inference methods, which provide the cell ordering required for TVG analysis, can vary greatly^11^. Although beyond the scope of this study, this variability should be considered when evaluating the total time needed to characterize gene expression dynamics from a raw expression matrix. ”

7. Is the multiple testing for finding an optimal K with the BIC in the PreTSA-K model already included in the multiple testing correction?

We thank the reviewer for the comment. The multiple testing correction in our analysis does not need to account for the step of selecting the optimal number of internal knots K using BIC in the PreTSA-K model. The BIC-based selection of K is a model selection procedure, not a hypothesis test, and it does not produce p-values. Therefore, it is not subject to multiple testing correction.

Multiple testing correction is applied only to the p-values from hypothesis tests that are performed after the model has been selected. Since BIC simply helps identify the most appropriate model based

on fit and complexity, it does not fall under the scope of procedures like FDR or Bonferroni correction. There is no need to adjust for the BIC step in the multiple testing framework.

8. As the authors write, the empirical p-value for the TVG determination captures the uncertainty in inferring the pseudotime, but at the cost of having to perform the potentially very expensive pseu-dotime inference 100 times. How does this compare to just using the computed F statistic for an F test, which would be 100x more efficient? As computational efficiency is the key contribution of this manuscript, this should be discussed.

We thank the reviewer for this comment. We would first like to emphasize that accounting for the uncertainty of inferred pseudotime is essential for rigorous identification of TVGs, as comprehensively demonstrated in the PseudotimeDE paper^13^. While the original implementation of PseudotimeDE has limited scalability due to its high computational cost, this is no longer a major concern for PreTSA. In the revised manuscript, we demonstrate that PreTSA can complete 100 permutations within a reasonable time, even on extremely large datasets. Therefore, PreTSA with permutation remains the default and recommended approach for identifying TVGs.

Nonetheless, for users who prioritize computational efficiency, the revised version of the package includes an option to identify TVGs using an F-test. We compared gene rankings based on p-values from the F-test and the permutation procedure and found them to be highly consistent (Supplementary Figure 3), indicating that the F-test yields results similar to those of the permutation-based approach.

### We have added Supplementary Figure 3 and the following content to the main manuscript

to present the comparison between results obtained from the permutation-based procedure and the F-test procedure:

“ To further improve computational efficiency, PreTSA also provides an option to identify TVGs using an F-test, which produces results similar to the permutation procedure (Supplementary Figure 3). However, the p-values from the F-test are not as statistically rigorous as those obtained from permutation. ”

### We have also added descriptions of using the F-test to identify TVGs in the Methods section:

“ PreTSA can also identify TVGs using the F-test to further improve computational efficiency, although statistical rigor is not guaranteed. Assuming fixed pseudotime values, the F-statistic intro-duced above follows an exact F-distribution with (*K* + 3*, n − K −* 4) degrees of freedom under *H*_0_. ”

9. The pseudotime comes often from methods for reconstructing developmental trajectories. These trajectories can have complicated topologies, e.g. branched or non-simply connected topologies. It seems that PreTSA is limited to non-branching line-segment like developmental trajectories. If so this limitation is crucial for practical applications in the pseudotime case.

We thank the reviewer for this comment and apologize for not making this point clear in the original manuscript. It is indeed possible for developmental trajectories to exhibit complex topologies, such as branching structures, where each branch may correspond to a distinct developmental or differentiation process. In such cases, PreTSA can be applied to each branch separately to identify genes that drive the corresponding processes. This approach is commonly adopted by many existing pseudotime analysis methods, including TSCAN and Monocle, which are compared in this study. Therefore, PreTSA can be applied to complex trajectories revealed by trajectory inference methods in the same manner as other widely used TVG analysis approaches.

### We have added the following content to the Conclusions and Methods section of the manuscript for further clarification:

“ While we only demonstrated PreTSA in a single trajectory for TVG analysis, it can also be applied to more complex topologies, such as branching structures, where each branch represents a distinct developmental or differentiation process. In these cases, PreTSA can be applied to each branch separately to uncover genes associated with the corresponding biological transitions. Thus, PreTSA can be effectively extended to complex developmental processes revealed by trajectory inference methods,

in the same way as other widely used time-varying gene analysis approaches. ”

“ Note that for trajectories with multiple branches, PreTSA needs to be applied multiple times, each time to a single branch separately. ”

10. The tensor product ansatz of cubic B splines for the spatial case implicitly restricts the func-tional space covered by the fit compared to full polynomials in x and y coordinates of degree 3: They constitute only separable functions in x and y, i.e. assume f(x,y)=g(x)*h(y)), although in general sep-arability should not hold. In particular, this assumption breaks the rotational symmetry of 2D space into a discrete subgroup: not all directions are equivalent and the model cannot provide the same fit for data rotated by e.g. 45°. This introduces artefacts in the modelling which were not discussed, quantified, or remedied in the current manuscript. A possible artefact is a more axis-aligned result, evidence for which could be the PreTSA result shown in Fig 2g. For the PreTSA case (not the more general PreTSA-K), a solution which keeps the symmetry is very simple: just include the full basis of polynomials of degree 3, instead of the separable subset appearing in the tensor product.

We thank the reviewer for this comment and agree that the current formulation of PreTSA may introduce an artifact where not all directions are modeled equivalently. To evaluate the impact of this potential artifact, we rotated the spatial coordinates of spots in the Visium human heart dataset by 30, 45, 60, and 90 degrees and then applied PreTSA. We compared the fitted gene expression values and gene rankings from the rotated data with those obtained from the original, unrotated dataset. The results (Supplementary Figure 28) show that both fitted gene expression values and gene rankings remain highly consistent, indicating that the artifact has minimal effect on gene expression fitting and SVG identification.

We note that while the limitation could be addressed by including the full basis of polynomials, as suggested by the reviewer, this is only feasible in the case without knots. There is no straightforward way to extend this approach when knots are introduced into the B-spline basis. However, knots are essential for capturing local variation in spatial gene expression patterns. Given that the artifact does not cause significant fitting issues, as shown above, and considering the importance of knots in the model, we have chosen to retain the current formulation of PreTSA.

### We have added Supplementary Figure 28 and the following content to the Conclusions section to describe this analysis:

“ In modeling spatial gene expression, PreTSA employs a tensor product of B-spline bases rather than the full polynomial basis, which may introduce an artifact whereby not all directions are treated equivalently. To assess the impact of this limitation, we rotated the spatial coordinates of spots in the Visium human heart data by various angles and applied PreTSA. The comparison of gene expression values and gene rankings between the original and rotated data showed strong consistency (Supplementary Figure 28), suggesting that this artifact has minimal impact on gene expression fitting and SVG identification. ”

11. Spatial scales are very relevant for the analysis of spatial data to analyze effects of whole tissues (global gradients within a tissue), tissue substructures (e.g., effects of distance to vascular structures), of cell neighbourship level (e.g. cell-cell communication effects) or even intracellular (e.g., reorgani-zation of organellar structures). As going down to finer spatial scales would blow up the number of necessary internal nodes orders of magnitude beyond the proposed maximum of K=5, it seems that PreTSA only applies to the global scale, which is often confounded with tissue handling and/or imag-ing artefacts. This should drastically limit the practical relevance of the method for the spatial use case.

We thank the reviewer for this comment. We agree that spatial data can be analyzed at multiple scales. PreTSA is scale agnostic and can be applied at any analysis scale based on the user’s input, not only at the global scale. The method does not assume a particular tissue size, number of cells, or spatial transcriptomics resolution. Applied to whole tissue sections, PreTSA identifies SVGs that reflect global gradients within the tissue. Applied to selected regions, it reveals finer grained expression patterns within tissue substructures. If whole tissue analyses raise concerns about handling or imaging artifacts, the tissue can be subset to mitigate these effects, and PreTSA can then be applied to the

selected region.

### We have added below content to the Discussion and Methods sections of the manuscript:

“ Spatial data can be analyzed at multiple scales, and PreTSA is scale agnostic, operating effectively from whole tissues to localized subregions based on the user’s input. The method does not assume a particular tissue size, number of cells, or spatial transcriptomics resolution. If global analyses raise concerns about handling or imaging artifacts, the tissue can be subset to mitigate these effects, then PreTSA can be applied within the chosen region, supporting robust multiscale spatial analysis. ”

“ Users can subset the ST data to focus on specific tissue regions, enable multiscale spatial analysis, or mitigate technical issues such as imaging artifacts. ”

12. The model assumes Gaussian distribution of the expression data, which for count data is clearly not the case (as can be seen in Fig 1d). This apparent mismatch should be discussed. That this is important is indicated in the curious diversity of the SPARK-group of benchmarked methods: the most recent method with speed comparable to PreTSA, SPARK-X, was introduced as a non-parametric fix of the earlier parametric SPARK and SPARK-G models which assumed Poisson or Gaussian distribu-tions. That the assumed distribution matters and has a strong impact on the results is also visualized by Figs 2j, S2c, S2d in the current manuscript: The three methods with the most similar results to PreTSA (GAM, SpatialDE, Spark-G) all assume Gaussian distribution.

We thank the reviewer for the comment. We first note that the input to PreTSA is log-transformed, library-size–normalized gene expression data, which are no longer raw counts. We agree that these inputs do not rigorously follow a normal distribution, although their empirical distribution is often close to normal despite the presence of excessive zeros.

To evaluate and compare the performance of PreTSA with other methods for identifying SVGs, including nonparametric approaches such as SPARK-X, we conducted a simulation study using scDe-sign3 to generate realistic spatial transcriptomics data with ground truth SVGs. Accuracy was assessed by the area under the precision–recall curve (AUPRC), and the results show that PreTSA ranks among the top-performing methods.

To assess false positive control, we also performed a null simulation in which the spatial coordinates of cells in a real dataset were randomly permuted. Since no true SVGs are expected, any detected signals are false positives. In this setting, PreTSA identified very few SVGs, demonstrating strong control of false discoveries.

For completeness, we also repeated both the scDesign3 simulation and the null simulation for TVG identification. Again, PreTSA achieved higher AUPRC values and effectively controlled false positives. In summary, these results suggest that although there is some discrepancy between the distribution assumed by the model and the empirical data distribution, PreTSA is still able to accurately identify TVGs and SVGs while controlling false positives. Correctly specifying the data distribution could fur-ther improve the statistical properties of the model, but it would substantially increase computational complexity, as introducing zero inflation eliminates the availability of a closed-form solution. Explor-ing whether comparable computational efficiency to PreTSA can be achieved under a zero-inflated

distribution may be a worthwhile direction for future work.

### We have added Figure 1d, 2d and Supplementary Figure 6, 15 to present the results of the simulation studies, and included the following content in the Conclusions section of the manuscript:

“ Although the normality assumption in PreTSA deviates from the empirical distribution of log-transformed gene expression data due to dropouts^5^, the method nonetheless achieves high accuracy in identifying TVGs and SVGs while effectively controlling false positives. Incorporating more complex distributions, such as zero-inflated models, could improve statistical rigor but would substantially increase computational complexity by eliminating the closed-form solution. Future research should focus on developing approaches that better balance distributional fidelity with computational efficiency. ”

13. Finally, a remark about the presentation: Fig 1b and 1c suggest that the right hand panels are zoomed versions of the left hand panels, which is not the case as GAM and PseudotimeDE are not

represented in the right hand panels. At least the point with the smallest number of cells should still be visible in the zoomed version, as they have smaller computation time than the values of the largest number of cells of PreTSA-K. Also the time axis on the left hand panels is on the linear scale with a scale break and on the right hand panels on a log scale - it would help the reader to put both on a log scale.

We thank the reviewer for the comment and agree that the previous figures could lead to biased interpretation. **We have revised Figure 1b-c, 2b-c** in two ways to improve interpretability. First, the zoomed-in plots now appear as crops of the original plots, ensuring that methods with data points and lines within the axis ranges are visible. Second, both the original and zoomed-in plots are now presented on the same scale to minimize confusion.

## Response to Reviewer 3

The authors present PreTSA, a memory and time efficient method for modeling temporal and spatial gene expression. The results presented in this manuscript demonstrate that PreTSA has a better performance in terms of running time and memory usage at different dataset sizes in comparison with other available methods, while preserving comparable results when detecting Temporal and Spatial Variable Genes. I have a few comments that may benefit the manuscript.

We sincerely thank the reviewer for the constructive feedback. We have carefully addressed all questions and comments in this revision, which we believe has substantially improved the manuscript. Our point-by-point responses to the comments are provided below.

1. Although the correlation and overlapping analysis show a good agreement between PreTSA and previous methods for the detection of TVG and SVG, I believe the manuscript is laking plots that show the direct output from PreTSA when applying it to the mentioned real and simulated datasets (e.g., a UMAP of cells colored using the pseudotime vs a UMAP with cell types)

We thank the reviewer for the comment. In the revised manuscript, we have added additional UMAP plots colored by different cell types for each pseudotime and spatial dataset. For pseudotime datasets, we also included UMAP plots colored by pseudotime values, and for spatial datasets, we included spatial plots colored by cell types. Together, these visualizations illustrate the temporal and spatial distribution of cell types, providing an overview of each dataset.

### These plots have been included as Supplementary Figure 29-33 in the revised manuscript.

2. While Fig. 2d-g suggests an interesting improvement in the detection of spatial patterns, the original expression shows a complex and subtle pattern that makes difficult to conclude the result is accurate. I suggest to include a second example where the original expression offers a cleared distribution.

We thank the reviewer for the comment. In the revised manuscript, we have included additional examples of genes with clearer spatial expression patterns. Specifically, we now show the spatial expression of ACTA1, the top-ranked SVG, which exhibits higher expression in the bottom-right region of the tissue. We also include MYL7, another top SVG, which shows higher expression on the top-right side of the tissue. In both cases, the fitted expression patterns by PreTSA closely resemble the original data.

### We have added Figure 2n-o, Supplementary Figure 18-19, and below contents to the Results section of the manuscript:

“ Notably, *ACTA1* shows elevated expression in the bottom-right region of the tissue, suggesting cardiac remodeling^10^ (Figure 2n), whereas *MYL7* is enriched in the top-right region, likely correspond-ing to atrial cardiomyocytes^12^ (Figure 2o, Supplementary Figure 18-19). ”

3. Fig. 2j shows the overlap proportion between PreTSA and other methods. I wonder how the spatial distribution of gene expression from the genes found using PreTSA but not detected on the other methods looks like. Do you observe particular patterns that explain why they are found using PreTSA but no with the other methods?

We thank the reviewer for the comment. We would first like to clarify that, given the large number of SVG identification methods included in the comparison, a top SVG identified by PreTSA is very likely to also be detected as a top SVG by at least one other method. Specifically, the top 10 SVGs identified by PreTSA also appeared among the top 30 ranked by PreTSA-K, GAM, and SPARK-G. Furthermore, 9 of these 10 genes were ranked within the top 30 by SpatialDE and nnSVG, with the remaining gene ranked within the top 50. Therefore, it is difficult to identify genes that are top-ranked by PreTSA but not recognized by other methods.

Nevertheless, we further examined SPARK and SPARK-X, whose results were less consistent with those of PreTSA. SPARK reported the top 10 PreTSA-identified SVGs among the 167 genes with the

smallest *p*-values but did not provide additional ranking metrics, preventing a direct comparison. By contrast, only 3 of the top 10 PreTSA SVGs were ranked within the top 30 by SPARK-X. Closer inspection revealed that genes such as FOS and EGR1, which display elevated expression across multiple tissue centers (Supplementary Figure 18-19), were ranked 99 and 168, respectively, by SPARK-X. A likely explanation is that the nonparametric modeling strategy used by SPARK-X is less effective at capturing such complex multi-center patterns, resulting in their lower rankings.

4. I suggest including the website link in the Data availability section, currently is only visible when exploring the GitHub repository.

We thank the reviewer for this comment. **We have added the website link to the Data Availability section:**

“ All datasets used in the study are publicly available. The R package PreTSA is publicly available at <https://github.com/haotian-zhuang/PreTSA>. Comprehensive tutorials are available at [https:](https://haotian-zhuang.github.io/PreTSA/)

[//haotian-zhuang.github.io/PreTSA/](https://haotian-zhuang.github.io/PreTSA/). ”

## References

[1] Koichi Araki, Masahiro Morita, Annelise G Bederman, Bogumila T Konieczny, Haydn T Kissick, Nahum Sonenberg, and Rafi Ahmed. Translation is actively regulated during the differentiation of cd8+ effector t cells. *Nature immunology*, 18(9):1046–1057, 2017.
[2] Yong Bai, Xiangyu Guo, Keyin Liu, Bingjie Zheng, Yilin Wei, Yingyue Wang, Wenxi Zhang, Qiuhong Luo, Jianhua Yin, Liang Wu, et al. Spaseg: unsupervised deep learning for multi-task analysis of spatially resolved transcriptomics. *Genome Biology*, 26(1):230, 2025.
[3] Aim´ee Bastidas-Ponce, Sophie Tritschler, Leander Dony, Katharina Scheibner, Marta Tarquis-Medina, Ciro Salinno, Silvia Schirge, Ingo Burtscher, Anika B¨ottcher, Fabian J Theis, et al. Com-prehensive single cell mrna profiling reveals a detailed roadmap for pancreatic endocrinogenesis. *Development*, 146(12):dev173849, 2019.
[4] Yoav Benjamini and Yosef Hochberg. Controlling the false discovery rate: a practical and powerful approach to multiple testing. *Journal of the Royal statistical society: series B (Methodological)*, 57(1):289–300, 1995.
[5] Wenpin Hou, Zhicheng Ji, Hongkai Ji, and Stephanie C Hicks. A systematic evaluation of single-cell rna-sequencing imputation methods. *Genome biology*, 21(1):218, 2020.
[6] Hui-Ping Hsu, Ming-Derg Lai, Jenq-Chang Lee, Meng-Chi Yen, Tzu-Yang Weng, Wei-Ching Chen, Jung-Hua Fang, and Yi-Ling Chen. Mucin 2 silencing promotes colon cancer metastasis through interleukin-6 signaling. *Scientific reports*, 7(1):5823, 2017.
[7] Jian Hu, Xiangjie Li, Kyle Coleman, Amelia Schroeder, Nan Ma, David J Irwin, Edward B Lee, Russell T Shinohara, and Mingyao Li. Spagcn: Integrating gene expression, spatial location and histology to identify spatial domains and spatially variable genes by graph convolutional network. *Nature methods*, 18(11):1342–1351, 2021.
[8] Peter Jantscheff, Luigi Terracciano, Adam Lowy, Katharina Glatz-Krieger, Fritz Grunert, Burkhard Micheel, Jens Bru¨mmer, Urs Laffer, Urs Metzger, Richard Herrmann, et al. Expression of ceacam6 in resectable colorectal cancer: a factor of independent prognostic significance. *Journal of clinical oncology*, 21(19):3638–3646, 2003.
[9] Michelli Faria de Oliveira, Juan Pablo Romero, Meii Chung, Stephen R Williams, Andrew D Gottscho, Anushka Gupta, Susan E Pilipauskas, Seayar Mohabbat, Nandhini Raman, David J Sukovich, et al. High-definition spatial transcriptomic profiling of immune cell populations in colorectal cancer. *Nature Genetics*, pages 1–12, 2025.
[10] Gianina Ravenscroft, Elyshia McNamara, Lisa M Griffiths, John M Papadimitriou, Edna C Harde-man, Anthony J Bakker, Kay E Davies, Nigel G Laing, and Kristen J Nowak. Cardiac *α*-actin over-expression therapy in dominant acta1 disease. *Human molecular genetics*, 22(19):3987–3997, 2013.
[11] Wouter Saelens, Robrecht Cannoodt, Helena Todorov, and Yvan Saeys. A comparison of single-cell trajectory inference methods. *Nature biotechnology*, 37(5):547–554, 2019.
[12] Yoel H Sitbon, Sunil Yadav, Katarzyna Kazmierczak, and Danuta Szczesna-Cordary. Insights into myosin regulatory and essential light chains: a focus on their roles in cardiac and skeletal muscle function, development and disease. *Journal of muscle research and cell motility*, 41(4):313–327, 2020.
[13] Dongyuan Song and Jingyi Jessica Li. Pseudotimede: inference of differential gene expression along cell pseudotime with well-calibrated p-values from single-cell rna sequencing data. *Genome biology*, 22(1):124, 2021.
[14] Dongyuan Song, Qingyang Wang, Guanao Yan, Tianyang Liu, Tianyi Sun, and Jingyi Jessica Li. scdesign3 generates realistic in silico data for multimodal single-cell and spatial omics. *Nature Biotechnology*, 42(2):247–252, 2024.
[15] Shuli Sun, Jixin Liu, Guojun Li, and Bingqiang Liu. Deepgft: identifying spatial domains in spatial transcriptomics of complex and 3d tissue using deep learning and graph fourier transform. *Genome Biology*, 26(1):153, 2025.
[16] Shiva K Tyagarajan and Jean-Marc Fritschy. Gephyrin: a master regulator of neuronal function?

*Nature Reviews Neuroscience*, 15(3):141–156, 2014.

[17] Sandra Verploegen, Jan-Willem J Lammers, Leo Koenderman, and Paul J Coffer. Identification and characterization of cklik, a novel granulocyte ca++/calmodulin-dependent kinase. *Blood, The Journal of the American Society of Hematology*, 96(9):3215–3223, 2000.
[18] Sanja Vickovic, G¨okcen Eraslan, Fredrik Salm´en, Johanna Klughammer, Linnea Stenbeck, Denis Schapiro, Tarmo A¨ ij¨o, Richard Bonneau, Ludvig Bergenstr˚ahle, Jos´e Fernand´ez Navarro, et al. High-definition spatial transcriptomics for in situ tissue profiling. *Nature methods*, 16(10):987–990, 2019.
[19] Sui Wang, Jingbo Yan, Daniel A Anderson, Yanwen Xu, Maneesh C Kanal, Zheng Cao, Christo-pher VE Wright, and Guoqiang Gu. Neurog3 gene dosage regulates allocation of endocrine and exocrine cell fates in the developing mouse pancreas. *Developmental biology*, 339(1):26–37, 2010.
[20] Per Westermark, Arne Andersson, and Gunilla T Westermark. Islet amyloid polypeptide, islet amyloid, and diabetes mellitus. *Physiological reviews*, 91(3):795–826, 2011.
[21] Thomas Wong, Reece Kang, and Kyuson Yun. The multi-faceted immune modulatory role of s100a4 in cancer and chronic inflammatory disease. *Frontiers in Immunology*, 16:1525567, 2025.
[22] Hao Xu, Shuyan Wang, Minghao Fang, Songwen Luo, Chunpeng Chen, Siyuan Wan, Rirui Wang, Meifang Tang, Tian Xue, Bin Li, et al. Spacel: deep learning-based characterization of spatial transcriptome architectures. *Nature Communications*, 14(1):7603, 2023.
[23] Zheying Zhang, Yongxia Wang, Jinghang Zhang, Jiateng Zhong, and Rui Yang. Col1a1 promotes metastasis in colorectal cancer by regulating the wnt/pcp pathway. *Molecular Medicine Reports*, 17(4):5037–5042, 2018.

**Second round of review**

**Reviewer 1**

**Reviewer 2**

In the revised manuscript the authors have added additional datasets and analyses, which significantly improved the quality of the manuscript and addressed many of my concerns. Some remaining/derived comments are below:

The revised manuscript contains more real datasets generated with more recent experimental techniques than the previously included Visium (which is the commercialization of the original "Spatial Transcriptomics"), HDST ("High Definition Spatial Transcriptomics", the academic high resolution version of the original "Spatial Transcriptomics") and Visium HD (the commercial high resolution version of Visium). These methods are all related by common ancenstry and still very similar in key properties apart from spatial resolution, e.g. they are all sequencing-based whole transcriptome approaches. The other unrelated group of current high-resolution spatial transcriptomics methods contains the targeted, single-molecule resolved smFISH-like methods (e.g., MERFISH/Vizgen MERSCOPE, 10x Xenium, and Nanostring CosMX). They have mostly a limited panel of 100s to 1000s of RNAs (except for CosMX with the whole transcriptome panel), and measure many (segmented) single cells per run. Unlike the further developments of the Visium branch of spatial transcriptomics, these single-molecule methods are the current driver in generating large spatial transcriptomics datasets. It would make a lot of sense to also include another example to showcase PreTSAs applicability for users of those platforms as well. This would be much more relevant than demonstrating it on HDST, which to the best of my knowlege is not commercially available and has never been used again after the initial publication of the method.

The revised manuscript includes an assessment of the benefit of using parallelization of competing methods on runtime and found that while other tools partially profit in terms of runtime, they tend to need a lot more memory when run in the paralellized setting. This is indeed a common phenomenon in parallelization. Yet this mostly points towards suboptimal parallelization tools and strategies. Data duplication can be minimal in most cases especially on a shared memory architecture where multiple processes can access the same data. In particular, the most critical task in PreTSA is implemented through "efficient matrix operations", which are the standard example for tasks to be efficiently parallelized across multiple cores without data duplication (the Linpack benchmark for supercomputers is just linear algebra operations and scales to millions of cores).
Depending on the R flavour used, it is possible to access this parallelism by using a threaded MKL (or similar) as backend for matrix operations. It would be interesting to see whether PreTSA on a threaded MKL based R installation becomes faster without sacrificing memory (MKL should achieve maximum performance in single and multi-threaded execution for matrix operations - and R should be able to benefit from that as well without changing a line of R code).
The authors note that high-performing tools in the field are often implemented in R, and cite SPARK-X. While according to the benchmarks in the revised manuscript, SPARK-X is indeed the highest performing tool in terms of runtime and memory consumption, it also does not seem to profit from parallelization, even for "large" datasets where parallelization should boost performance. This adds another question mark on using R for high-performing code, regardless of the field.
The new python version implemented for revision is an interesting development and it would be interesting to see how this performs. However, as always there are a lot of experiments necessary to get peak performance here as well. A few pointers in case the authors want to continue with this python implementation: Depending on the number of right hand sides, solving multiple linear systems can be faster than inverting a matrix; numba jitting code using just matrix multiplications and linalg operations is unlikely to speed things up as the individual operations are efficient already - numba jitting helps more for for-loops and other lower level constructs; to see the libraries which are actually used for parallelism by numpy etc. and to control the degree of parallelism they can access, one can either use environment variables or threadpoolctl - using a threaded MKL is important here just as it is in the R-case.

The revised manuscript contains an assessment of the degree of breaking of continuous rotational symmetry by the tensor product ansatz in Supp. Fig. 28, which shows that the correlation of the fitted gene expression and gene rankings is 1 for 90 degree rotations (corresponding to the conserved subgroup of discrete rotations) and goes down to values of around 0.9 for other angles. According to Figs S1 and S2, a value of around 0.9 in Pearson correlation corresponds to adding 4-5/1-2 internal knots in the TVG/SVG case. According to Fig 2l, a value of 0.9 in Spearman correlation corresponds to swapping PreTSA by GAM. Furthermore this assessment is done just for a single dataset and selection of K. While I agree that the extra artefacts by the loss of rotational symmetry should rarely be dominant, I would recommend to tone down the description of consistency with rotated data in the Conclusions ("strong consistency", "minimal impact"), and also mention the loss of symmetry in the methods section during introduction of the tensor product ansatz.

Similarly, the revised manuscript contains an assessment of the robustness of the results wrt changes of K, presented in Figs S1, S2. The reported consistency of the results with different numbers of K goes down as far as for swapping PreTSA with completely different methods (GAM, SPARK-G, SPARK-X, SpatialDE, Figure 2l). So different choices of K are like using completely different methods. Still the authors call this "robust to the numbers of knots" which does not really seem to be supported by the data. But this motivates very well the automatic K-selection to get a well justified choice for K. It would be interesting in this context to see which values of K PreTSA-K actually chooses - is it mostly K=0?

In response to the spatial scales comment, the authors state that subsetting the input to selected regions could solve the issue that a single PreTSA run only looks at spatial scales close to the spatial input size. But this does not appear to solve the issue for all but the largest scales in current high-resolution methods which all support near cm sized samples and resolution down to µm scale: Take VisiumHD as an example. If one starts with a 6.5mm diameter VisiumHD dataset and one is interested in SVGs on cellular neighbourhood level, say 65µm, one needs to tile the sample in 100*100=10000 subsets, run those individually through PreTSA, and then devise a custom result aggregation to get the SVGs at that length scale. And this is still ignoring all the information down to 2µm which is the resolution of VisiumHD. This suggests that even with subsetting the input data, PreTSA cannot be used in practice to analyse data at scales significantly smaller than the sample size. In this view, I think that the new statements around PreTSAs current ability to "support robust multiscale spatial analysis" are misleading, as small length scales (like a 65µm neighbourhood in a 6.5mm VisiumHD sample) can only be assessed in practice with the current PreTSA version by throwing away a lot of data (all but one bin, i.e. 99.99% in the example).
An example of apparent spatial struture which is just smoothed out by the PreTSA fit (on tissue scale; Fig S19) is that FOS/JUNB hotspot in the center of the Visium heart dataset (Fig S18). In this case by chance an independent large scale spatial variability came to the rescue and still made the genes visible as PreTSA-SVGs and included in the figure, but there are certainly a lot of instances with similarly striking spatial structure only on smaller length scales which are not found by PreTSA - because it is effectively limited to the largest spatial scales. For higher resolution data like VisiumHD more spatial features are just not resolvable in the global scale PreTSA fit (Figs S26, S27). I think that this apparent limitation has to be openly declared in the discussion instead of hidden in user input of a superficially spatial scale agnostic method: while the absolute spatial scales are irrelevant to PreTSA, it does get the information about the relative range of scales in the dataset from pixel size and distance to sample size. And PreTSA chooses to ignore them.
BTW: This opens up a trivial algorithmic improvement to make PreTSA much more performant memory-wise and runtime-wise: You can just bin the input data regardless of the actual amount of pixels into a 100x100 grid or so (and similar bin the cells along pseudotime into 100 bins), average the gene expression per bin, plug that into the current PreTSA, and get very similar SVGs and smoothed gene expression, and achive perfect logarithmic scaling (if the binning is implemented well) in runtime and perfect constant scaling in memory (depending on the procedure to read the data from disk).

And finally a couple of points about the presentation:

Fig S14: Some methods don't have datapoints for all numbers of spots even though they are far from hitting the maximum memory. Are they excluded because they hit the wallclock limit? Regardless the reason, this should be explained in the Figure legend.

Fig S28 uses Pearson correlation for fitted gene values and Spearman correlation for the gene rankings, while Figs. S1, S2 use Pearson for both. Is this a typo or is there a good reason for making the ranking correlations non-comparable between figures? 1i, 2l, S16c, S16e also use Spearman for gene rankings.

What is the difference between Figs 2l and S16e?

Fig 1c right panel (zoom-in) looks nearly identical to the left panel (not zoomed), as they have nearly the same y-range (0-125 VS 0-168). Either zoom in more (like in Fig 1b) or show the same data as in the left panel but put the y-axis on a log scale (for consistency do the same in Fig 1b, and in 2b, 2c)

Fig 1 (except panels a, d, g), 2 (except panels a, d, e), S4, S5, S7, S13, S14: unclear which dataset was used (the legend should contain that information).

Fig S5: It would be good to see the 1 core data for PseudotimeDE as well (for the runs where it finished in time), especially as the main text claims that "While PseudotimeDE with 10 cores substantially reduced computation time, it also required much larger memory". Generally, include the data for 1 core and 10 core where it is available, e.g. in Fig S14. If they are there, but just not visible as they all sit on top of each other on the linear scale, go to log scale and/or choose different marker styles for different methods.

Figs 1b, 1c, 2b, 2c, S4, S5, S13, S14: Maybe the PreTSA default (1 core) should consistently get the solid line, while the other-tools-only feature to run with 10 cores can have the dashed line. might be a matter of taste, but dashed looks like less important while it is currently used for PreTSA.

Also a matter of taste: For consistency one could use the same color scale (range of values) for original and fitted expression to make the data in e.g. Fig 2e,g,h (also S18,19, S22,23, S26,27) better comparable.

**Reviewer 3**

The manuscript has significantly improved. The authors have added more biological examples, benchmarking using multicore options for comparison with methods that offer that option, and provided a deeper methodology description. The authors have answered my previous comments and suggestions. I have one last suggestion:

In the discussion, it’s mentioned that PreTSA can also be applied to more complex topologies, such as branching structures, where each branch represents a distinct developmental or differentiation process. PreTSA can then be applied to each branch separately to uncover genes associated with the corresponding biological transition. It would add good value to the paper if you demonstrate this statement on a real dataset.

**Authors Response**

**Point-by-point responses to the reviewers’ comments:**

## Response to Reviewer 2

In the revised manuscript the authors have added additional datasets and analyses, which significantly improved the quality of the manuscript and addressed many of my concerns. Some remaining/derived comments are below:

We sincerely thank the reviewer for the positive feedback. We have carefully addressed the re-maining comments in this revision, which we believe has further improved the manuscript. Our point-by-point responses to the comments are provided below.

1. The revised manuscript contains more real datasets generated with more recent experimental tech-niques than the previously included Visium (which is the commercialization of the original ”Spatial Transcriptomics”), HDST (”High Definition Spatial Transcriptomics”, the academic high resolution version of the original ”Spatial Transcriptomics”) and Visium HD (the commercial high resolution version of Visium). These methods are all related by common ancenstry and still very similar in key properties apart from spatial resolution, e.g. they are all sequencing-based whole transcriptome approaches. The other unrelated group of current high-resolution spatial transcriptomics methods contains the targeted, single-molecule resolved smFISH-like methods (e.g., MERFISH/Vizgen MER-SCOPE, 10x Xenium, and Nanostring CosMX). They have mostly a limited panel of 100s to 1000s of RNAs (except for CosMX with the whole transcriptome panel), and measure many (segmented) single cells per run. Unlike the further developments of the Visium branch of spatial transcriptomics, these single-molecule methods are the current driver in generating large spatial transcriptomics datasets. It would make a lot of sense to also include another example to showcase PreTSAs applicability for users of those platforms as well. This would be much more relevant than demonstrating it on HDST, which to the best of my knowlege is not commercially available and has never been used again after the initial publication of the method.

We thank the reviewer for the comment. As suggested, in this revision we added a new example using a human lung cancer sample generated with 10x Xenium. The dataset contained 377 genes and 151,677 cells. We performed analyses consistent with those applied to the other spatial datasets, and the results show that PreTSA, PreTSA-K, and SPARK-X were the fastest methods, in agreement with conclusions from the other datasets. In addition, PreTSA-K identified genes whose fitted spatial expression patterns are consistent with known biological features of this sample.

We have added new Figures 7h-j and Supplementary Figure 32-34, and added the below descriptions in the Results section:

“ Finally, we applied PreTSA to identify SVGs in a human lung cancer sample generated using 10x Xenium. Unlike the previous examples based on spot-based platforms, 10x Xenium represents an image-based platform. After filtering, the dataset contained 377 genes and 151,677 cells. PreTSA, PreTSA-K, and SPARK-X each completed the analysis within one minute, whereas other methods either required substantially longer runtimes or failed due to excessive memory usage (Figure 7h, Supple-mentary Figure 32). Notably, many top-ranked SVGs identified by PreTSA-K showed clear spatial localization patterns consistent with known lung cancer tissue architecture (Supplementary Figure 33-34). For example, *EPCAM*, which encodes the epithelial cell adhesion molecule, exhibited higher expression in malignant epithelial regions^1^ (Figure 7i). In contrast, *AGER*, which plays a critical role in pulmonary regulation, was predominantly expressed in the alveolar epithelium^6^ (Figure 7j). ”

2. The revised manuscript includes an assessment of the benefit of using parallelization of competing methods on runtime and found that while other tools partially profit in terms of runtime, they tend to need a lot more memory when run in the paralellized setting. This is indeed a common phenomenon in parallelization. Yet this mostly points towards suboptimal parallelization tools and strategies. Data duplication can be minimal in most cases especially on a shared memory architecture where multiple processes can access the same data. In particular, the most critical task in PreTSA is implemented through ”efficient matrix operations”, which are the standard example for tasks to be efficiently par-allelized across multiple cores without data duplication (the Linpack benchmark for supercomputers is just linear algebra operations and scales to millions of cores).

Depending on the R flavour used, it is possible to access this parallelism by using a threaded MKL (or similar) as backend for matrix operations. It would be interesting to see whether PreTSA on a threaded MKL based R installation becomes faster without sacrificing memory (MKL should achieve maximum performance in single and multi-threaded execution for matrix operations - and R should be able to benefit from that as well without changing a line of R code).

We thank the reviewer for this comment. As suggested, we explored the technical possibility of using a threaded MKL-based R installation to further increase the computational efficiency of PreTSA. To facilitate installation and usage of such an R environment, we consulted the system administrator of the Duke Compute Cluster (DCC), the computing infrastructure used for developing and testing PreTSA. However, the system administrator advised against this approach, noting that it would require recompiling a new R version with the Intel compiler to avoid library version mismatches and other incompatibilities. As we do not have administrative privileges, we were unable to perform this installation on DCC without their support.

We also attempted to compile a threaded MKL-based R installation locally on a MacBook Pro with Apple M2 Pro CPUs, following both the Intel oneAPI Math Kernel Library (oneMKL) guide and the Intel Math Kernel Library (Intel MKL) 2019 installation guide. Unfortunately, in both cases we encountered technical issues potentially arising from incompatibilities between Intel MKL and Apple systems, which we were unable to resolve.

For these reasons, we were unable to install a threaded MKL-based R environment and therefore could not test whether it would provide additional speedup for PreTSA. Nevertheless, we have added a new paragraph in the Discussion section discussing the possibility of using a threaded MKL-based R installation to further improve the computational efficiency of PreTSA for users with the technical capability to implement it:

“ In this study, PreTSA was tested using a standard installation of R. For users with suitable hardware and technical expertise, non-standard R installations may further increase the computational efficiency of PreTSA. For example, using a threaded MKL-based R installation may reduce computation time by more efficiently parallelizing matrix operations. ”

The authors note that high-performing tools in the field are often implemented in R, and cite SPARK-X. While according to the benchmarks in the revised manuscript, SPARK-X is indeed the highest performing tool in terms of runtime and memory consumption, it also does not seem to profit from parallelization, even for ”large” datasets where parallelization should boost performance. This adds another question mark on using R for high-performing code, regardless of the field.

We thank the reviewer for the comment. We acknowledge that R may not be the most suitable pro-gramming language for parallelization or achieving maximum computational efficiency. Our primary reason for choosing R for PreTSA is its compatibility with many software tools developed for single-cell and spatial omics data, many of which are hosted on Bioconductor, arguably one of the largest and most widely used bioinformatics software repositories that supports only R packages. Using R for PreTSA enables users to build seamless analysis pipelines that integrate PreTSA with existing R packages for single-cell analysis. As suggested by the reviewer in the previous revision, we have also created a Python version of PreTSA for users who prefer Python.

In this revision, we have added the following content to the Discussion section to highlight the

possibility of using other programming languages:

“ Moreover, although PreTSA was implemented in R for compatibility with existing packages for single-cell and spatial analysis such as Seurat^4^, Monocle^7^, and TSCAN^5^, Python and other pro-gramming languages may be more suitable for parallelization or achieving maximum computational efficiency. A Python implementation of PreTSA, which shows slightly reduced running time compared to the R version (Supplementary Figure 36), is also provided in the PreTSA software package. ”

The new python version implemented for revision is an interesting development and it would be interesting to see how this performs. However, as always there are a lot of experiments necessary to get peak performance here as well. A few pointers in case the authors want to continue with this python implementation: Depending on the number of right hand sides, solving multiple linear systems can be faster than inverting a matrix; numba jitting code using just matrix multiplications and linalg operations is unlikely to speed things up as the individual operations are efficient already - numba jitting helps more for for-loops and other lower level constructs; to see the libraries which are actually used for parallelism by numpy etc. and to control the degree of parallelism they can access, one can either use environment variables or threadpoolctl - using a threaded MKL is important here just as it is in the R-case.

We thank the reviewer for the comment. As suggested, we have used the linear solver rather than explicitly computing a matrix inverse, and we have removed the Numba JIT compilation in this revi-sion. In addition, we evaluated the running time of the R and Python implementations of PreTSA. The results show that the Python implementation is slightly faster than the R implementation, although the difference is minimal.

We have added a new Supplementary Figure 36 in the revision to demonstrate the running time of the Python and R implementations of PreTSA. We have also added the following content to the Discussion section to remind readers that a Python implementation is available for use:

“ A Python implementation of PreTSA, which shows slightly reduced running time compared to the R version (Supplementary Figure 36), is also provided in the PreTSA software package. ”

3. The revised manuscript contains an assessment of the degree of breaking of continuous rotational symmetry by the tensor product ansatz in Supp. Fig. 28, which shows that the correlation of the fitted gene expression and gene rankings is 1 for 90 degree rotations (corresponding to the conserved subgroup of discrete rotations) and goes down to values of around 0.9 for other angles. According to Figs S1 and S2, a value of around 0.9 in Pearson correlation corresponds to adding 4-5/1-2 internal knots in the TVG/SVG case. According to Fig 2l, a value of 0.9 in Spearman correlation corresponds to swapping PreTSA by GAM. Furthermore this assessment is done just for a single dataset and se-lection of K. While I agree that the extra artefacts by the loss of rotational symmetry should rarely be dominant, I would recommend to tone down the description of consistency with rotated data in the Conclusions (”strong consistency”, ”minimal impact”), and also mention the loss of symmetry in the methods section during introduction of the tensor product ansatz.

We thank the reviewer for the comment. In this round of revision, we extended the assessment to all four spatial datasets, for both PreTSA and PreTSA-K. The results, which are now included in the updated Supplementary Figure 35, suggest that the smallest correlation between fitted expression or gene rankings before and after applying rotation is approximately 0.9 across all cases.

As suggested by the reviewer, we have now toned down the description of consistency in the revised manuscript:

“ The comparison of fitted gene expression values and gene rankings between the original and rotated data showed generally high consistency (Supplementary Figure 35), suggesting that this artifact has a mild impact on gene expression fitting and SVG identification. ”

We have also added the following content to the Methods section when introducing the tensor product formulation:

“ Note that this formulation breaks the rotational symmetry of the 2D space into a discrete sub-

group, meaning that not all directions are treated equivalently and the model cannot guarantee identical fits for data rotated by, for example, 45°. See the Discussion section for a more detailed discussion. ”

4. Similarly, the revised manuscript contains an assessment of the robustness of the results wrt changes of K, presented in Figs S1, S2. The reported consistency of the results with different numbers of K goes down as far as for swapping PreTSA with completely different methods (GAM, SPARK-G, SPARK-X, SpatialDE, Figure 2l). So different choices of K are like using completely different methods. Still the authors call this ”robust to the numbers of knots” which does not really seem to be supported by the data. But this motivates very well the automatic K-selection to get a well justified choice for K. It would be interesting in this context to see which values of K PreTSA-K actually chooses - is it mostly K=0?

We thank the reviewer for the comment. In the revised manuscript, we have removed the claim of being “robust to the number of knots”. As suggested by the reviewer, we have added a new Supplementary Figure 3 to summarize the number of cases in which PreTSA-K selects different values of K. As expected, in most cases PreTSA-K selects K = 0.

The relevant content now reads as follows:

“ Users have the option to select a flexible mode, PreTSA-K, which automatically determines the number of knots in the B-spline basis (Methods). Supplementary Figures 1–2 show how different choices for the number of knots affect gene expression fitting. Supplementary Figure 3 shows the number of cases in which PreTSA-K selects different values of K across all datasets in this study. ”

5. In response to the spatial scales comment, the authors state that subsetting the input to selected regions could solve the issue that a single PreTSA run only looks at spatial scales close to the spatial input size. But this does not appear to solve the issue for all but the largest scales in current high-resolution methods which all support near cm sized samples and resolution down to µm scale: Take VisiumHD as an example. If one starts with a 6.5mm diameter VisiumHD dataset and one is interested in SVGs on cellular neighbourhood level, say 65µm, one needs to tile the sample in 100*100=10000 subsets, run those individually through PreTSA, and then devise a custom result aggregation to get the SVGs at that length scale. And this is still ignoring all the information down to 2µm which is the resolution of VisiumHD. This suggests that even with subsetting the input data, PreTSA cannot be used in practice to analyse data at scales significantly smaller than the sample size. In this view, I think that the new statements around PreTSAs current ability to ”support robust multiscale spatial analysis” are misleading, as small length scales (like a 65µm neighbourhood in a 6.5mm VisiumHD sample) can only be assessed in practice with the current PreTSA version by throwing away a lot of data (all but one bin, i.e. 99.99% in the example). An example of apparent spatial struture which is just smoothed out by the PreTSA fit (on tissue scale; Fig S19) is that FOS/JUNB hotspot in the center of the Visium heart dataset (Fig S18). In this case by chance an independent large scale spatial variability came to the rescue and still made the genes visible as PreTSA-SVGs and included in the figure, but there are certainly a lot of instances with similarly striking spatial structure only on smaller length scales which are not found by PreTSA - because it is effectively limited to the largest spatial scales. For higher resolution data like VisiumHD more spatial features are just not resolvable in the global scale PreTSA fit (Figs S26, S27). I think that this apparent limitation has to be openly declared in the discussion instead of hidden in user input of a superficially spatial scale agnostic method: while the absolute spatial scales are irrelevant to PreTSA, it does get the information about the relative range of scales in the dataset from pixel size and distance to sample size. And PreTSA chooses to ignore them.

We thank the reviewer for this thoughtful and concrete comment. We agree that, when applied once over an entire sample, PreTSA behaves as a domain-level smoother that emphasizes variation at or above a nontrivial fraction of the analysis domain, which can attenuate features at micrometer to tens-of-micrometers scales in centimeter-scale fields of view. Our prior wording that PreTSA “supports robust multiscale spatial analysis” was overly broad. We have revised the text to clarify that PreTSA is agnostic to absolute spatial units, yet its effective sensitivity is governed by the spatial extent of the user-defined domain. We now explicitly acknowledge that a single global fit is not intended to resolve

very fine spatial structure in high-resolution data. The revised paragraph removes the potentially misleading phrasing, states the limitation plainly, and positions PreTSA as a method that captures broad spatial trends while indicating that resolving much smaller scales requires localized analyses beyond a single global application.

We have revised the corresponding paragraph in the Discussion section of the manuscript:

“ Spatial data can be analyzed at multiple scales, and PreTSA is agnostic to absolute units, operating over a user defined analysis domain. When applied once to an entire sample, PreTSA primarily captures tissue scale trends, which can attenuate or obscure fine scale variation at micrometer to tens of micrometers. Although the method does not assume a specific tissue size, cell number, or spatial transcriptomics resolution, detection of features at scales much smaller than the analysis domain is beyond the scope of a single global fit. Subsetting the tissue and applying PreTSA within a localized region might mitigate global artifacts and enable focused analyses, however this approach does not fully eliminate the inherent limitation regarding very small scale features. ”

6. BTW: This opens up a trivial algorithmic improvement to make PreTSA much more performant memory-wise and runtime-wise: You can just bin the input data regardless of the actual amount of pixels into a 100x100 grid or so (and similar bin the cells along pseudotime into 100 bins), average the gene expression per bin, plug that into the current PreTSA, and get very similar SVGs and smoothed gene expression, and achive perfect logarithmic scaling (if the binning is implemented well) in runtime and perfect constant scaling in memory (depending on the procedure to read the data from disk).

We thank the reviewer for this suggestion. In this round of revision, we have added an option in the PreTSA function to perform data binning on the input temporal or spatial data before fitting the model. We compared running time, memory usage, and gene ranking results on the Visium HD human colon cancer dataset using the original PreTSA model and the binned version of PreTSA. The results show that both runtime and memory usage decreased substantially with binning, as expected. The overall gene ranking results from the binned data remained consistent with those from the original dataset (Pearson correlation = 0.95). However, we note that while the binning strategy further improves computational efficiency, it may reduce the spatial resolution provided by spatial profiling platforms. Given that PreTSA can already complete full-resolution analyses within a reasonable timeframe, we have made this binning function optional and recommend its use primarily when runtime or memory demands on the original data become prohibitive.

We have added new Supplementary Figure 37 and 38, and the following description in the Discussion section of the manuscript:

“ Finally, PreTSA provides an optional binning strategy that averages gene expression values across cells within small spatial and temporal bins before fitting the model (Methods). This approach further reduces running time and memory usage (Supplementary Figure 37) while producing fitted gene rankings similar to those obtained without binning (Supplementary Figure 38), although it may reduce the spatial resolution of the original data. Thus, we recommend its use primarily when runtime or memory demands on the original data become prohibitive. ”

And finally a couple of points about the presentation:

We thank the reviewer for the comments on the presentation. We have addressed these points accordingly. We should note that the revised manuscript has been reformatted as a “Methods” article, and most of the figures and supplementary figures have been reorganized or relabeled. Nevertheless, the changes suggested by the reviewer have been preserved during the reorganization.

7. Fig S14: Some methods don’t have datapoints for all numbers of spots even though they are far from hitting the maximum memory. Are they excluded because they hit the wallclock limit? Regardless the reason, this should be explained in the Figure legend.

We thank the reviewer for pointing this out. Some data points are excluded from the memory-usage

plots because their computational time exceeded one week, so we were unable to obtain their actual memory usage. We have now added this information to the figure legends of all related figures.

8. Fig S28 uses Pearson correlation for fitted gene values and Spearman correlation for the gene rank-ings, while Figs. S1, S2 use Pearson for both. Is this a typo or is there a good reason for making the ranking correlations non-comparable between figures? 1i, 2l, S16c, S16e also use Spearman for gene rankings.

We thank the reviewer for the comment. We would like to clarify that when applied to rank data, Pearson and Spearman correlations yield identical results. For consistency, we have now used Pearson correlation throughout the manuscript.

9. What is the difference between Figs 2l and S16e?

The difference between the two figures is that one presented results from PreTSA, and the other presented results from PreTSA-K.

10. Fig 1c right panel (zoom-in) looks nearly identical to the left panel (not zoomed), as they have nearly the same y-range (0-125 VS 0-168). Either zoom in more (like in Fig 1b) or show the same data as in the left panel but put the y-axis on a log scale (for consistency do the same in Fig 1b, and in 2b, 2c)

We thank the reviewer for the comment. We have increased the zoom level in the right panel to provide a clearer view of the differences.

11. Fig 1 (except panels a, d, g), 2 (except panels a, d, e), S4, S5, S7, S13, S14: unclear which dataset was used (the legend should contain that information).

We thank the reviewer for the comment. We have now clearly indicated the dataset used in each figure in the figure legends.

12. Fig S5: It would be good to see the 1 core data for PseudotimeDE as well (for the runs where it finished in time), especially as the main text claims that ”While PseudotimeDE with 10 cores sub-stantially reduced computation time, it also required much larger memory”. Generally, include the data for 1 core and 10 core where it is available, e.g. in Fig S14. If they are there, but just not visible as they all sit on top of each other on the linear scale, go to log scale and/or choose different marker styles for different methods.

We thank the reviewer for the comment. We would like to clarify that the results for PseudotimeDE using one core were already included in the previous version of the manuscript but were difficult to see because they overlapped with results from other methods. In this revision, we have added log-scale panels so that all points can be seen.

13. Figs 1b, 1c, 2b, 2c, S4, S5, S13, S14: Maybe the PreTSA default (1 core) should consistently get the solid line, while the other-tools-only feature to run with 10 cores can have the dashed line. might be a matter of taste, but dashed looks like less important while it is currently used for PreTSA.

We thank the reviewer for the comment. As suggested by the reviewer, we have revised the line styles so that single-core runs use solid lines and multi-core runs use dashed lines for all methods, including PreTSA.

14. Also a matter of taste: For consistency one could use the same color scale (range of values) for original and fitted expression to make the data in e.g. Fig 2e,g,h (also S18,19, S22,23, S26,27) better comparable.

We thank the reviewer for the comment. We experimented with using the same color scale across plots, but the spatial expression patterns become difficult to discern in some cases. Thus, we chose to retain separate color scales to achieve clearer visualization.

## Response to Reviewer 3

The manuscript has significantly improved. The authors have added more biological examples, bench-marking using multicore options for comparison with methods that offer that option, and provided a deeper methodology description. The authors have answered my previous comments and suggestions. I have one last suggestion:

We sincerely thank the reviewer for the positive feedback. We have carefully addressed the final suggestion in this revision, which we believe has further improved the manuscript. Our point-by-point responses to the comments are provided below.

1. In the discussion, it’s mentioned that PreTSA can also be applied to more complex topologies, such as branching structures, where each branch represents a distinct developmental or differentiation process. PreTSA can then be applied to each branch separately to uncover genes associated with the corresponding biological transition. It would add good value to the paper if you demonstrate this statement on a real dataset.

We thank the reviewer for the comment. In this revision, we demonstrate how PreTSA can be applied to a dataset with multiple branches from a mouse pancreas endocrine development study. The dataset contains four endocrine developmental lineages. PreTSA was applied to each lineage separately, and fitted gene expression patterns were visualized for marker genes of each lineage. The results suggest that PreTSA can identify lineage-specific temporal gene expression patterns.

The results are shown in the new Figure 4, and we have added the following content to the Results section of the revised manuscript:

“ To demonstrate that PreTSA can be applied to complex topologies, including branching structures, we applied it to study endocrine development in the mouse pancreas^2^. The dataset contains four endocrine lineages, alpha, beta, delta, and epsilon cell differentiation (Figure 4a). In the beta cell lineage, which contains the largest number of cells among the four, PreTSA and PreTSA-K completed the analysis in 8 and 18 minutes, respectively, whereas PseudotimeDE required 7.3 hours on a single core and 1.4 hours on 10 cores, while also using more memory (Figure 4b, Supplementary Figure 11). Consistent with known endocrine development, GO terms enriched among the TVGs identified by PreTSA-K include insulin metabolic process and signal peptide processing (Supplementary Figure 12–15). The top TVGs identified by PreTSA-K include both shared genes and branch-specific markers (Figure 4c–e, Supplementary Figure 16). For example, *Neurog3* initially increases as the master transcription factor initiating endocrine commitment, but subsequently decreases once its transient role is fulfilled as cells progress into the pre-endocrine state^2^^,^ ^8^ (Figure 4c). *Chga*, which is involved in hormone processing, increases during endocrine differentiation and then slightly decreases as endocrine cells mature^2^ (Figure 4d). In addition, canonical markers for each endocrine cell type exhibit increasing trends within their respective branches^3^ (Figure 4e). ”

## References

[1] PA Baeuerle and Olivier Gires. Epcam (cd326) finding its role in cancer. *British journal of cancer*, 96(3):417–423, 2007.
[2] Aim´ee Bastidas-Ponce, Sophie Tritschler, Leander Dony, Katharina Scheibner, Marta Tarquis-Medina, Ciro Salinno, Silvia Schirge, Ingo Burtscher, Anika B¨ottcher, Fabian J Theis, et al. Com-prehensive single cell mrna profiling reveals a detailed roadmap for pancreatic endocrinogenesis. *Development*, 146(12):dev173849, 2019.
[3] Lauren E Byrnes, Daniel M Wong, Meena Subramaniam, Nathaniel P Meyer, Caroline L Gilchrist, Sarah M Knox, Aaron D Tward, Chun J Ye, and Julie B Sneddon. Lineage dynamics of murine pancreatic development at single-cell resolution. *Nature communications*, 9(1):3922, 2018.
[4] Yuhan Hao, Stephanie Hao, Erica Andersen-Nissen, William M Mauck, Shiwei Zheng, Andrew Butler, Maddie J Lee, Aaron J Wilk, Charlotte Darby, Michael Zager, et al. Integrated analysis of multimodal single-cell data. *Cell*, 184(13):3573–3587, 2021.
[5] Zhicheng Ji and Hongkai Ji. Tscan: Pseudo-time reconstruction and evaluation in single-cell rna-seq analysis. *Nucleic acids research*, 44(13):e117–e117, 2016.
[6] Markus A Queisser, Fotini M Kouri, Melanie Konigshoff, Malgorzata Wygrecka, Uwe Schubert, Oliver Eickelberg, and Klaus T Preissner. Loss of rage in pulmonary fibrosis: molecular relations to functional changes in pulmonary cell types. *American journal of respiratory cell and molecular biology*, 39(3):337–345, 2008.
[7] Cole Trapnell, Davide Cacchiarelli, Jonna Grimsby, Prapti Pokharel, Shuqiang Li, Michael Morse, Niall J Lennon, Kenneth J Livak, Tarjei S Mikkelsen, and John L Rinn. The dynamics and regulators of cell fate decisions are revealed by pseudotemporal ordering of single cells. *Nature biotechnology*, 32(4):381–386, 2014.
[8] Sui Wang, Jingbo Yan, Daniel A Anderson, Yanwen Xu, Maneesh C Kanal, Zheng Cao, Christo-pher VE Wright, and Guoqiang Gu. Neurog3 gene dosage regulates allocation of endocrine and exocrine cell fates in the developing mouse pancreas. *Developmental biology*, 339(1):26–37, 2010.

**Third round of review**

**Reviewer 2**

In the current revision the authors have addressed most of my comments, added a dataset of a different class of spatial transcriptomics methods and even followed up in code on the recommendations to further improve the performance of the method. This is commendable. In the process of revision the authors improved the paper a lot.

A few last comments:

1. Presentation of the new Xenium dataset in Figure 7i and j: The fitted expression looks squished in y-direction. This might be due to using the same number of knots in x and y direction, regardless of the actual physical aspect ratio of the sample? It might be worth to add the possibility to (automatically) select a number of internal knots separately for x and y direction which aligns with the physical aspect ratio or warn the user (and recoomend cropping the sample?) when samples with aspect ratio far from 1 are used.

2. About installing a threaded MKL-based R: Installation and compilation issues can be nicely circumvented as non-admin user by using a package manager like conda. It is incredibly simple, see e.g. https://stackoverflow.com/questions/58834940/conda-install-r-essentials-with-mkl or ask the AI of your choice. On newer Macbooks with Apple Silicon, MKL does indeed not work. On a compute cluster (which is most often still x86-based these days) this should not be an issue.

3. The analysis of the automatically chosen number of internal knots K is very interesting. It shows that for the cases which do not contain much information apart from the largest spatial scales, K is chosen to be 0 (as there is nothing to be gained from higher spatial resolution of the fit there), but is often nonzero for the datasets which do contain a lot of structure at finer spatial scales. On the one hand this shows that the automatic K selection is working reasonably well, but on the other it shows also that the spatial resolution of PreTSA for these high resolution datasets is limited by K, maybe strongly as the distribution over K did not show any signs of decay at the maximum tested K=5. This is expected and consistent and ties into the discussion about spatial scales.

Could you add in the figure legend for Figure S3, a proportion of which cases this is? Is it over replicates, genes, or something else?

Last remarks about presentation (probably the copy editor would have caught this, as well):

4. Supplementary Figure references are broken in two cases: Line 44 refers to "Supplementary Figures 12" instead of "Supplementary Figures 1 and 2" and in Line 96 the reference is to "Supplementary Figure 1215" instead of "Supplementary Figures 12-15"

5. Line 281: in the pdf version the "°" of "45°" became a "r" with a "ˇ" on top...

**Reviewer 3**

The manuscript has significantly improved. The authors have answered my previous suggestion showing that PreTSA can be applied to complex topologies, including branching structures, using a real dataset. I’m satisfied with the contents of this manuscript and I consider it ready for acceptance.

A small comment, there seems to be a dash missing in line 96, when referencing the Supplementary figure number “(Supplementary Figure 1215).” And a comma missing in line 97 saying “Figure 4ce”

**Response to Reviewers**

## Response to Reviewer 2

In the current revision the authors have addressed most of my comments, added a dataset of a differ-ent class of spatial transcriptomics methods and even followed up in code on the recommendations to further improve the performance of the method. This is commendable. In the process of revision the authors improved the paper a lot. A few last comments:

We sincerely thank the reviewer for the positive feedback. We have carefully addressed the re-maining comments in this revision, which we believe has further improved the manuscript. Our point-by-point responses to the comments are provided below.

1. Presentation of the new Xenium dataset in Figure 7i and j: The fitted expression looks squished in y-direction. This might be due to using the same number of knots in x and y direction, regardless of the actual physical aspect ratio of the sample? It might be worth to add the possibility to (automatically) select a number of internal knots separately for x and y direction which aligns with the physical aspect ratio or warn the user (and recoomend cropping the sample?) when samples with aspect ratio far from 1 are used.

We thank the reviewer for this comment. We have added an option in the software that allows users to select the number of internal knots separately for the x and y directions. In addition, we now provide a message to alert users when samples with aspect ratios far from 1 are analyzed, recommending either selecting different numbers of knots for the two directions or cropping the sample when appropriate.

2. About installing a threaded MKL-based R: Installation and compilation issues can be nicely cir-cumvented as non-admin user by using a package manager like conda. It is incredibly simple, see e.g. [https://nam11.safelinks.protection.outlook.com/?url=https%3A%2F%2Fstackoverflow.com%](https://nam11.safelinks.protection.outlook.com/?url=https%3A%2F%2Fstackoverflow.com%2Fquestions%2F58834940%2Fconda-install-r-essentials-with-mkl&data=05%7C02%7Czhicheng.ji%40duke.edu%7C8c13be6c66e74198935908de5e8064a9%7Ccb72c54e4a314d9eb14a1ea36dfac94c%7C0%7C0%7C639052102702347427%7CUnknown%7CTWFpbGZsb3d8eyJFbXB0eU1hcGkiOnRydWUsIlYiOiIwLjAuMDAwMCIsIlAiOiJXaW4zMiIsIkFOIjoiTWFpbCIsIldUIjoyfQ%3D%3D%7C0%7C%7C%7C&sdata=HKRsn0PyBdTTw%2BmkUOqxYwCt8d0wui7EZwkh2RVdQ7g%3D&reserved=0) [2Fquestions%2F58834940%2Fconda-install-r-essentials-with-mkl&data=05%7C02%7Czhiche](https://nam11.safelinks.protection.outlook.com/?url=https%3A%2F%2Fstackoverflow.com%2Fquestions%2F58834940%2Fconda-install-r-essentials-with-mkl&data=05%7C02%7Czhicheng.ji%40duke.edu%7C8c13be6c66e74198935908de5e8064a9%7Ccb72c54e4a314d9eb14a1ea36dfac94c%7C0%7C0%7C639052102702347427%7CUnknown%7CTWFpbGZsb3d8eyJFbXB0eU1hcGkiOnRydWUsIlYiOiIwLjAuMDAwMCIsIlAiOiJXaW4zMiIsIkFOIjoiTWFpbCIsIldUIjoyfQ%3D%3D%7C0%7C%7C%7C&sdata=HKRsn0PyBdTTw%2BmkUOqxYwCt8d0wui7EZwkh2RVdQ7g%3D&reserved=0) [ng.ji%40duke.edu%7C8c13be6c66e74198935908de5e8064a9%7Ccb72c54e4a314d9eb14a1ea36dfac9](https://nam11.safelinks.protection.outlook.com/?url=https%3A%2F%2Fstackoverflow.com%2Fquestions%2F58834940%2Fconda-install-r-essentials-with-mkl&data=05%7C02%7Czhicheng.ji%40duke.edu%7C8c13be6c66e74198935908de5e8064a9%7Ccb72c54e4a314d9eb14a1ea36dfac94c%7C0%7C0%7C639052102702347427%7CUnknown%7CTWFpbGZsb3d8eyJFbXB0eU1hcGkiOnRydWUsIlYiOiIwLjAuMDAwMCIsIlAiOiJXaW4zMiIsIkFOIjoiTWFpbCIsIldUIjoyfQ%3D%3D%7C0%7C%7C%7C&sdata=HKRsn0PyBdTTw%2BmkUOqxYwCt8d0wui7EZwkh2RVdQ7g%3D&reserved=0) [4c%7C0%7C0%7C639052102702347427%7CUnknown%7CTWFpbGZsb3d8eyJFbXB0eU1hcGkiOnRydWUsIlYi](https://nam11.safelinks.protection.outlook.com/?url=https%3A%2F%2Fstackoverflow.com%2Fquestions%2F58834940%2Fconda-install-r-essentials-with-mkl&data=05%7C02%7Czhicheng.ji%40duke.edu%7C8c13be6c66e74198935908de5e8064a9%7Ccb72c54e4a314d9eb14a1ea36dfac94c%7C0%7C0%7C639052102702347427%7CUnknown%7CTWFpbGZsb3d8eyJFbXB0eU1hcGkiOnRydWUsIlYiOiIwLjAuMDAwMCIsIlAiOiJXaW4zMiIsIkFOIjoiTWFpbCIsIldUIjoyfQ%3D%3D%7C0%7C%7C%7C&sdata=HKRsn0PyBdTTw%2BmkUOqxYwCt8d0wui7EZwkh2RVdQ7g%3D&reserved=0) [OiIwLjAuMDAwMCIsIlAiOiJXaW4zMiIsIkFOIjoiTWFpbCIsIldUIjoyfQ%3D%3D%7C0%7C%7C%7C&sdata=](https://nam11.safelinks.protection.outlook.com/?url=https%3A%2F%2Fstackoverflow.com%2Fquestions%2F58834940%2Fconda-install-r-essentials-with-mkl&data=05%7C02%7Czhicheng.ji%40duke.edu%7C8c13be6c66e74198935908de5e8064a9%7Ccb72c54e4a314d9eb14a1ea36dfac94c%7C0%7C0%7C639052102702347427%7CUnknown%7CTWFpbGZsb3d8eyJFbXB0eU1hcGkiOnRydWUsIlYiOiIwLjAuMDAwMCIsIlAiOiJXaW4zMiIsIkFOIjoiTWFpbCIsIldUIjoyfQ%3D%3D%7C0%7C%7C%7C&sdata=HKRsn0PyBdTTw%2BmkUOqxYwCt8d0wui7EZwkh2RVdQ7g%3D&reserved=0) [HKRsn0PyBdTTw%2BmkUOqxYwCt8d0wui7EZwkh2RVdQ7g%3D&reserved=0](https://nam11.safelinks.protection.outlook.com/?url=https%3A%2F%2Fstackoverflow.com%2Fquestions%2F58834940%2Fconda-install-r-essentials-with-mkl&data=05%7C02%7Czhicheng.ji%40duke.edu%7C8c13be6c66e74198935908de5e8064a9%7Ccb72c54e4a314d9eb14a1ea36dfac94c%7C0%7C0%7C639052102702347427%7CUnknown%7CTWFpbGZsb3d8eyJFbXB0eU1hcGkiOnRydWUsIlYiOiIwLjAuMDAwMCIsIlAiOiJXaW4zMiIsIkFOIjoiTWFpbCIsIldUIjoyfQ%3D%3D%7C0%7C%7C%7C&sdata=HKRsn0PyBdTTw%2BmkUOqxYwCt8d0wui7EZwkh2RVdQ7g%3D&reserved=0) or ask the AI of your choice. On newer Macbooks with Apple Silicon, MKL does indeed not work. On a compute cluster (which is most often still x86-based these days) this should not be an issue.

We thank the reviewer for this comment. We have added information to the Discussion section noting that threaded MKL-based R installations can be obtained using a package manager such as conda. In addition, we have added this information to the GitHub tutorials.

The relevant content now reads as follows:

“ For users with suitable hardware and technical expertise, non-standard R installations may fur-ther increase the computational efficiency of PreTSA. For example, using a threaded MKL-based R installation, such as one installed via a package manager like conda, may reduce computation time by more efficiently parallelizing matrix operations. ”

3. The analysis of the automatically chosen number of internal knots K is very interesting. It shows that for the cases which do not contain much information apart from the largest spatial scales, K is chosen to be 0 (as there is nothing to be gained from higher spatial resolution of the fit there), but is often nonzero for the datasets which do contain a lot of structure at finer spatial scales. On the one hand this shows that the automatic K selection is working reasonably well, but on the other it shows also that the spatial resolution of PreTSA for these high resolution datasets is limited by K, maybe strongly as the distribution over K did not show any signs of decay at the maximum tested K=5. This is expected and consistent and ties into the discussion about spatial scales. Could you add in the figure legend for Figure S3, a proportion of which cases this is? Is it over replicates, genes, or something else?

We thank the reviewer for the positive feedback. We have clarified in the figure legend for Figure S3 that the reported proportions correspond to the proportion of genes for which different numbers of internal knots were selected.

Last remarks about presentation (probably the copy editor would have caught this, as well):

We thank the reviewer for the comments on the presentation. We have addressed these points accordingly.

4. Supplementary Figure references are broken in two cases: Line 44 refers to ”Supplementary Figures 12” instead of ”Supplementary Figures 1 and 2” and in Line 96 the reference is to ”Supplementary Figure 1215” instead of ”Supplementary Figures 12-15”

We thank the reviewer for pointing this out. To avoid rendering issues, we have standardized all dash usage to plain hyphens (-) throughout the manuscript.

5. Line 281: in the pdf version the ”°” of ”45°” became a ”r” with a ”ˇ” on top...

We thank the reviewer for pointing this out. We have replaced it with a LaTeX-native representation (45*^◦^*) to ensure correct rendering.

## Response to Reviewer 3

The manuscript has significantly improved. The authors have answered my previous suggestion show-ing that PreTSA can be applied to complex topologies, including branching structures, using a real dataset. I’m satisfied with the contents of this manuscript and I consider it ready for acceptance.

We sincerely thank the reviewer for the positive feedback. We have carefully addressed the final comment in this revision, which we believe has further improved the manuscript. Our point-by-point responses to the comments are provided below.

1. A small comment, there seems to be a dash missing in line 96, when referencing the Supplementary figure number “(Supplementary Figure 1215).” And a comma missing in line 97 saying “Figure 4ce”

We thank the reviewer for pointing this out. To avoid rendering issues, we have standardized all dash usage to plain hyphens (-) throughout the manuscript.
